# CRISPR‐based editing of the ω‐ and γ‐gliadin gene clusters reduces wheat immunoreactivity without affecting grain protein quality

**DOI:** 10.1111/pbi.14231

**Published:** 2023-11-17

**Authors:** Zitong Yu, Ural Yunusbaev, Allan Fritz, Michael Tilley, Alina Akhunova, Harold Trick, Eduard Akhunov

**Affiliations:** ^1^ Wheat Genetic Resources Center Kansas State University Manhattan KS USA; ^2^ Department of Plant Pathology Kansas State University Manhattan KS USA; ^3^ Department of Agronomy Kansas State University Manhattan KS USA; ^4^ USDA‐ARS Grain Quality and Structure Research Unit Manhattan KS USA; ^5^ Integrated Genomic Facility Kansas State University Manhattan KS USA

**Keywords:** wheat immunotoxicity, gliadin gene clusters, gene editing

## Abstract

Wheat immunotoxicity is associated with abnormal reaction to gluten‐derived peptides. Attempts to reduce immunotoxicity using breeding and biotechnology often affect dough quality. Here, the multiplexed CRISPR‐Cas9 editing of cultivar Fielder was used to modify gluten‐encoding genes, specifically focusing on ω‐ and γ‐gliadin gene copies, which were identified to be abundant in immunoreactive peptides based on the analysis of wheat genomes assembled using the long‐read sequencing technologies. The whole‐genome sequencing of an edited line showed mutation or deletion of nearly all ω‐gliadin and half of the γ‐gliadin gene copies and confirmed the lack of editing in the α/β‐gliadin genes. The estimated 75% and 64% reduction in ω‐ and γ‐gliadin content, respectively, had no negative impact on the end‐use quality characteristics of grain protein and dough. A 47‐fold immunoreactivity reduction compared to a non‐edited line was demonstrated using antibodies against immunotoxic peptides. Our results indicate that the targeted CRISPR‐based modification of the ω‐ and γ‐gliadin gene copies determined to be abundant in immunoreactive peptides by analysing high‐quality genome assemblies is an effective mean for reducing immunotoxicity of wheat cultivars while minimizing the impact of editing on protein quality.

## Introduction

Wheat gluten defines the breadmaking properties of dough and underlies immune reaction to wheat‐based products (Shewry and Tatham, 2016). The efforts to reduce wheat immunotoxicity often negatively affected dough quality (Gil‐Humanes *et al*., 2010, 2014). The question remains whether it is feasible to selectively modify copies or parts of gluten‐encoding genes to substantially reduce wheat immunotoxicity without affecting the physicochemical properties of dough important for breadmaking.

The gluten network/gluten macropolymer (GMP) formed by disulphide bonds (DBs) between the cysteine residues of glutenins and gliadins defines the quality of dough (Shewry and Tatham, 1997). The inter‐molecular DBs between high molecular weight glutenin subunits (HMW‐GS) extend gluten network, whereas intra‐molecular DBs formed by gliadins terminate glutenin polymerization (Shewry and Halford, 2002). The impact of ω‐gliadins, which lack cysteine residues, on the gluten network is expected to be lower than that of α/β‐ and γ‐gliadins, which could form three and four intramolecular DBs, respectively (Visschers and de Jongh, 2005; Wieser, 2007). This is consistent with improved dough mixing time and tolerance observed in the RNAi transgenic lines with the ω1,2‐gliadin expression suppressed (Altenbach *et al*., 2019). Considering that ω‐gliadins represent only a small fraction of all gliadins, their suppression will likely have a limited negative impact on dough quality.

Celiac disease, food allergy and wheat‐dependent exercise‐induced anaphylaxis (WDEIA) are associated with a reaction to gluten induced by the oral intake of wheat gluten components resistant to enzymatic degradation. As a result, the repetitive peptides rich in proline (Pro, P) and glutamine (Gln, Q) accumulate in the small intestine (Scherf *et al*., 2016). Among gluten fractions, gliadins are the major carriers of toxic epitopes. The genes encoding ω‐ and γ‐gliadins are tightly clustered at three loci *Gli‐A1*, *Gli‐B1* and *Gli‐D1* on chromosomes 1A, 1B and 1D, respectively. The genes encoding α/β‐gliadins are located on the short arm of chromosome 6, named *Gli‐A2*, *Gli‐B2* and *Gli‐D2* (Branlard *et al*., 2001). Celiac disease and food allergy are caused by all three gliadin subtypes; while the WDEIA is associated with ω5‐gliadins encoded by the genes located at *Gli‐B1* loci (Shewry and Tatham, 2016). The ω1,2‐gliadins encoded by the genes located at *Gli‐A1* and *Gli‐D1* loci contain multiple peptides enriched in repeats containing the highly immunoreactive QQPFP motif. Food allergy‐related toxic epitopes are prominent in all gliadin subtypes, with the highest number present in ω‐gliadins, followed by γ‐ and α/β‐gliadins. Peptides including PQQPFP, QPQQPFP, and QQFPQQQ motives are frequently linked with food allergy. The toxic epitopes including QQFPQQQ motif associated with WDEIA are most frequent in ω5‐gliadins located on chromosome 1B (Juhász *et al*., 2018).

A reduction of gluten toxicity was often accompanied by a decrease in protein quality. The RNAi lines showing an 80% reduction in γ‐gliadin expression and reduced reaction against the R5 monoclonal antibody (mAb) showed reduced dough strength (Gil‐Humanes *et al*., 2008, 2012). Likewise, a 62%–67% reduction in immunoreactivity to the R5 and G12 mAbs achieved by the CRISPR‐Cas9 editing of 35 of 45 α‐gliadin genes was accompanied by a decrease in SDS sedimentation volume and reduced dough quality (Sánchez‐León *et al*., 2018). However, the RNAi suppression of ω1,2‐gliadins significantly reduced reactivity of wheat flour proteins to serum IgG and IgA antibodies and improved both mixing time and tolerance in transgenic lines (Altenbach *et al*., 2019). Similarly, the CRISPR‐based editing of some γ‐gliadin loci on chromosomes 1B and 1D showed that their editing could reduce immunoreactivity measured using R5 antibodies without affecting end‐use quality (Liu *et al*., 2023). These studies suggest that some gliadin gene copies could be suppressed without substantial impact on protein quality and indicate that ω‐gliadins and likely γ‐gliadins could be more promising targets for CRISPR‐based editing than α/β‐gliadins.

Accurate annotation of multigene families in complex wheat genome is critical for their successful editing. The recently released genome assembly of the transformation‐amenable cultivar Fielder (Sato *et al*., 2021) generated using HiFi reads provides a unique opportunity for identifying gliadin genes enriched for immunoreactive peptides and designing guides for their targeted editing. Here, we analysed the distribution of 11 immunoreactive peptides binding to R5 mAb and six peptides binding to G12 mAb (Schopf and Scherf, 2018) across the three gliadin gene families annotated in four high‐quality genome assemblies of cultivars Fielder (Sato *et al*., 2021), Kariega (Athiyannan *et al*., 2022), Chinese Spring and LongReach Lancer (Walkowiak *et al*., 2020). Since most ω‐gliadins lack cysteine residues to form disulphide crosslinks in the gluten network, and the proportion of ω‐ and γ‐gliadins in gluten is lower than that of α/β‐gliadins, we expect that CRISPR‐Cas9‐mediated editing of ω‐ and γ‐gliadin gene clusters should reduce gliadin toxicity and maintain protein quality. The multiplex editing was performed in cv. Fielder using gRNAs targeting all ω‐ and a subset of γ‐gliadin gene copies. The whole‐genome sequencing and comparative analyses of the edited and non‐edited lines derived from the progeny of the same T_0_ transgenic plant revealed large‐ and small‐scale deletions in gliadin gene clusters or individual genes in the edited line. The analyses of gliadin protein content, SDS‐extractable and ‐unextractable polymeric protein profiles, and viscoelastic properties of dough demonstrated a significant reduction in ω‐ and γ‐gliadin protein content and lack of impact on protein quality in the edited line. The monoclonal antibodies showed a 47‐fold reduction in immunoreactivity of gluten in edited lines compared to non‐edited transgenic lines. These results indicate that improved genome assemblies and annotation facilitate the identification of gliadin gene copies with higher immunogenic potential making feasible targeted editing of complex gene clusters in wheat and provide opportunities for reducing its immunotoxicity without affecting end‐use quality traits.

## Results

### Distribution of immunogenic peptides across gliadin genes in cv. Fielder genome

To investigate the distribution of immunogenic peptides across gliadin gene clusters, we have annotated the cv. Fielder genome (see Materials and methods). We have identified five, eight and five copies of ω‐gliadin genes in chromosomes 1A, 1B and 1D, respectively. Translation of these ω‐gliadin genes suggests that two, two and five copies on 1A, 1B and 1D, respectively, are functional. Out of seven, eight and nine copies of the γ‐gliadin genes in chromosomes 1A, 1B and 1D, respectively, five, seven and nine copies had uninterrupted an open reading frame. For α/β‐gliadin genes, there are 11, 27 and 14 copies on chromosomes 6A, 6B and 6D, respectively, with 10, 15 and 9 copies being functional.

**Figure 1:**
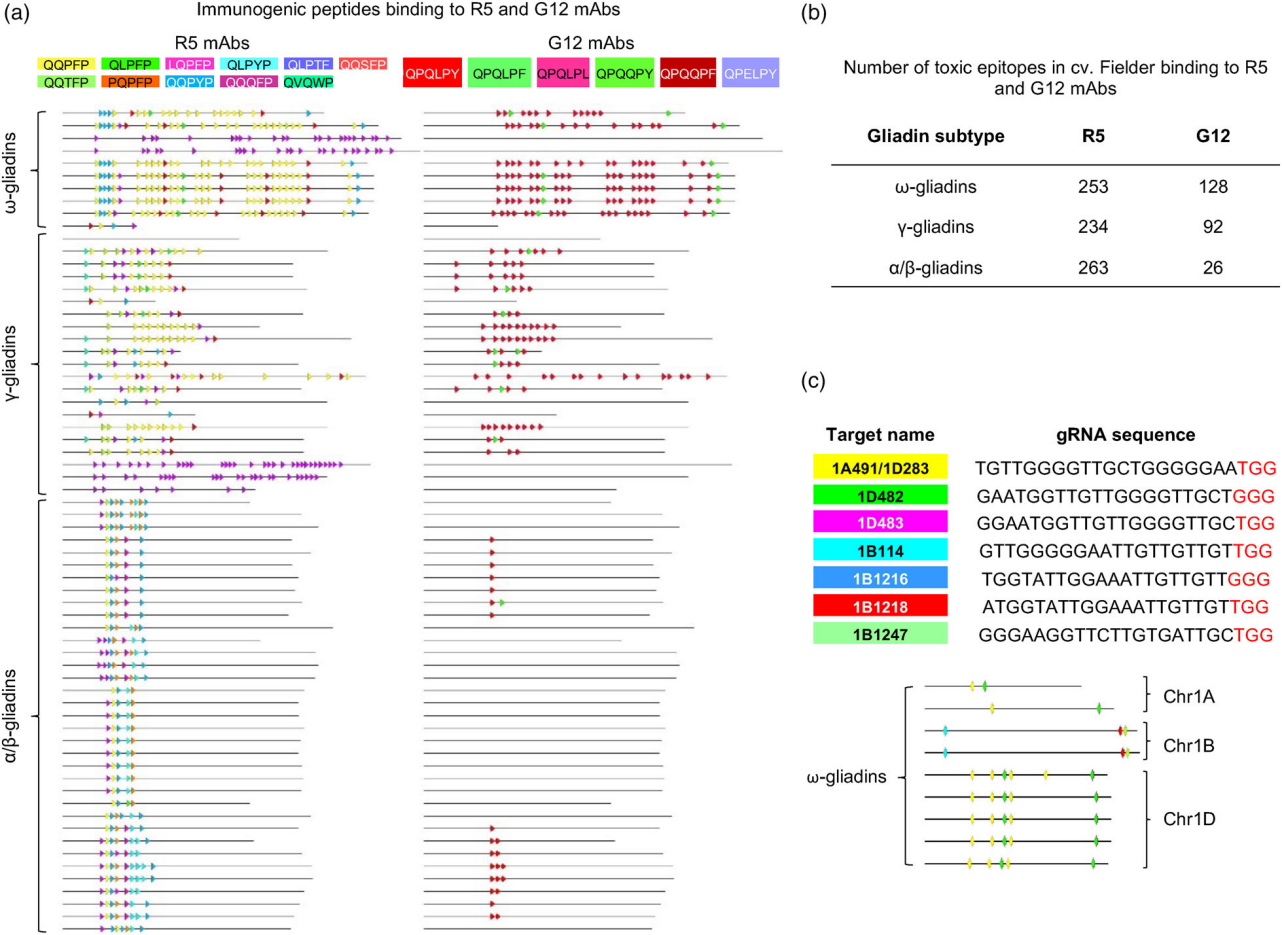
Distribution of toxic epitopes binding to the R5 and G12 mAbs across gliadin genes in cultivar Fielder. (a) The amino acid motifs of the 11 toxic epitopes binding to R5 mAb and the six toxic epitopes binding to G12 mAb: the 17 motifs were indicated by different background colours. The distributions of the 11 toxic epitopes binding to R5 mAb (left) and the six toxic epitopes binding to G12 mAb (right) across all three gliadin subtypes of cultivar Fielder: the different toxic epitopes were indicated by the arrows with corresponding colour shown in a; the distributions of the 17 toxic epitopes binding to R5 and G12 mAbs across all three gliadin subtypes of cultivars Kariega, Chinese Spring and LongReach Lancer were shown in Figure S1. (b) The number of toxic epitopes binding to R5 and G12 mAbs in each gliadin subtype of cultivar Fielder; the number of peptides binding to R5 and G12 mAbs from all gliadin subtypes of cultivars Kariega, Chinese Spring and LongReach Lancer is shown in Table S1. (c) Seven gRNAs designed for gliadin gene editing: three gRNAs target ω‐gliadins on chromosomes 1A and 1D and four gRNAs target ω‐gliadins on chromosome 1B. The PAM site NGG was indicated by the red font; the distribution of seven gRNAs across nine functional copies of the ω‐gliadin genes including two on chromosome 1A, two on chromosome 1B and five on chromosome 1D. The colour coding for gRNA target sites corresponds to colours shown in c. The gRNA‐targeted regions within the ω‐ and γ‐gliadin genes with 0 to 3 mismatches are shown in Table S2.

The R5 mAb raised against rye secalin binds to 11 gliadin toxic epitopes including QQPFP, QLPFP, LQPFP, QLPYP, QLPTF, QQSFP, QQTFP, PQPFP, QQPYP, QQQFP, QVQWP; the G12 mAb raised against the 33‐mer gliadin peptide can interact with six toxic epitopes including QPQLPY, QPQLPF, QPQLPL, QPQQPY, QPQQPF, QPELPY (Schopf and Scherf, 2018; Figure 1a). We analysed the distribution of 11 immunoreactive peptides binding to R5 mAb and six peptides binding to G12 mAb across the three gliadin gene families annotated in four high‐quality assemblies of cultivars Fielder (Sato *et al*., 2021), Kariega (Athiyannan *et al*., 2022), Chinese Spring and LongReach Lancer (Walkowiak *et al*., 2020) (Figure 1a; Figure S1). The number of R5‐ and G12‐binding peptides in ω‐ / γ‐gliadins was 1.1–1.8 and 4.7–9.8 times higher, respectively, than that in α/β‐gliadins, with 487 vs. 263 and 220 vs. 26 in Fielder, 358 vs. 245 and 136 vs. 22 in Kariega, 194 vs. 185 and 84 vs. 18 in Chinese Spring, and 198 vs. 156 and 78 vs. 8 in LongReach Lancer (Figure 1b; Table S1). These results suggest that more substantial reduction in the R5 and G12 immunoreactivity could be achieved by editing the ω‐ and γ‐gliadin genes than by editing the α/β‐gliadin genes.

### CRISPR‐Cas9‐mediated editing of the ω‐ and γ‐gliadin genes in cv. Fielder

Due to a higher number of R5‐ and G12‐binding peptides in ω‐gliadins compared to γ‐gliadins, design of guide RNAs (gRNAs) was primarily focused on the ω‐gliadin genes. Among the ω‐gliadin gRNAs, the preference was given to those that can also target some of the γ‐gliadin gene copies. This strategy provides an opportunity to completely remove the ω‐gliadin genes, which have a lower impact on end‐use quality than the γ‐gliadin genes (Altenbach *et al*., 2019; Gil‐Humanes *et al*., 2008, 2012), and also to reduce the number of γ‐gliadin gene copies sharing similar targets with the ω‐gliadin. A total of three gRNAs were designed to target ω‐gliadin loci on chromosomes 1A and 1D, including gRNAs 1A491/1D283, 1D482 and 1D483; and four gRNAs were designed to target loci on chromosome 1B, including gRNAs 1B114, 1B1216, 1B1218 and 1B1247 (Figure 1c). These gRNAs had from 0 to 3 mismatches in the targeted repetitive regions within the ω‐ and γ‐gliadin genes (Table S2). Each gRNA was subcloned into a separate Csy4‐zCas9 vector, combined in equimolar proportions into a single pool and co‐transformed into cv. Fielder.

A total of 435 T_1_ plants were obtained from 32 tillers of eight transgenic Cas9‐positive T_0_ plants. The derivatives of the T_0_ transgenic regenerant 387 have been primarily used in our study. The T_0_ plant 387 produced 4 tillers, which were labelled as 387‐1, 387‐2, 387‐3 and 387‐4. The PCR analysis showed that tillers 387‐1 and 387‐3 were Cas9 positive and each had an identical set of four gRNA constructs. The tillers 387‐2 and 387‐4 were negative for Cas9 and gRNA. The 14 T_1_ generation plant produced from the Cas9‐positive tiller 387‐1 were all Cas9‐negative. Out of seven T_1_ generation plants produced from tiller 387‐3, only one was Cas9 positive. Similar non‐mendelian inheritance of transgenes in the progeny of T_0_ plants has been previously observed in rice and wheat (Hamada *et al*., 2017; Xu *et al*., 2015).

Three PCR primer pairs were used to screen plants for the presence of gene edits in the nine functional copies of the ω‐gliadin genes (Tables S3 and S4). Due to the complexity and high levels of similarity, we experienced difficulties with designing specific primers for the γ‐gliadin gene copies and opted to use whole‐genome sequencing to characterize gene edits in this gene family (see whole‐genome sequencing results below). PCR screening detected fragment deletions in the ω‐gliadin genes located on chromosomes 1A and 1D of all seven T_1_ plants derived from T_0_ line 387‐3, including plants 387‐3‐1, 387‐3‐2, 387‐3‐3, 387‐3‐4, 387‐3‐5, 387‐3‐6, and 387‐3‐7 (Figure S2), while no fragment deletions were found in the gene copies on chromosome 1B. The next‐generation sequencing (NGS) showed that T_0_ line 387‐3 was transformed with four gRNAs, including 1A491/1D283, 1B114, 1B1247, and 1D482. The T_1_ progenies of 387‐3 were negative for Cas9 sequence, except for one plant (387‐3‐7). The lack of a PCR product of the same size as that obtained for the control suggests that the 387‐3‐6 line has the highest number of edited ω‐gliadin gene copies (Figure S2). The PCR analysis of the T_2_ population including 183 lines derived from 387 to 3‐6 revealed that all lines are fixed for fragment deletions (Figure S3), indicating that the T_1_ line 387‐3‐6 is homozygous for the edited variants of the ω‐gliadin genes. Further analyses of editing events were conducted in the T_1_ line 387‐3‐6.

The initial characterization of editing events in line 387‐3‐6 was performed by the NGS of PCR amplicons generated for the ω‐gliadin genes. The fragment deletions ranging from 18 to 111‐bp were detected in one, two and five copies of ω‐gliadin genes on chromosomes 1A, 1B and 1D, respectively (Figure S4; Table S5). Nearly all reads mapping to these gliadin gene loci carried mutated variants confirming that line 387‐3‐6 is homozygous for the edited alleles of the ω‐gliadin genes. Because designing PCR primers for amplifying all gene copies in the ω‐ and γ‐gliadin gene clusters was not feasible due to their repetitive nature, more detailed analysis of on‐ and off‐target editing events was conducted by whole genome sequencing of the 387‐3‐6 line and the non‐edited line 387‐1‐8 derived from the same T_0_ plant. The TruSeq DNA PCR‐free libraries were prepared for both lines and sequenced at ~10× genome coverage using Illumina technology (2 × 150‐bp).

First, by mapping reads to the Cas9‐gRNA constructs we confirmed that both lines, 387‐3‐6 and 387‐1‐8 are free of the transgenic constructs used for wheat transformation. Then, the reads from both lines were mapped to the Fielder genome generated using the long‐read PacBio sequencing data (Sato *et al*., 2021). The Fielder genome annotation identified nine and 22 functional ω‐ and γ‐gliadin gene copies, respectively. The genome‐wide variant calling performed by comparing the edited and non‐edited lines in the regions outside of the gliadin genes identified only 1743 sites differentiating 387‐3‐6 from 387 to 1‐8, with only 29 sites overlapping within the gene coding regions, indicating that both lines have nearly identical genetic backgrounds.

**Figure 2:**
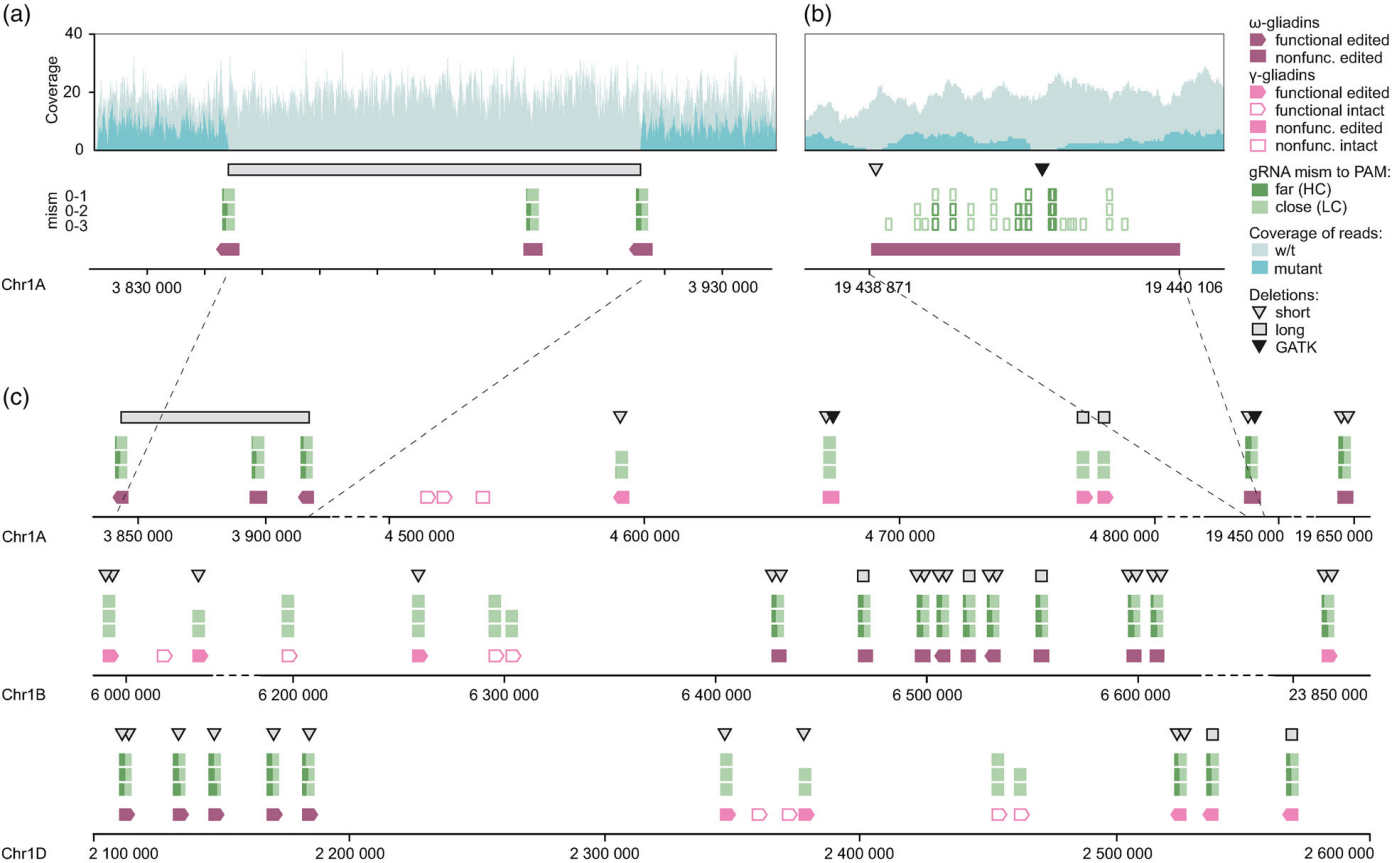
Summary of gene editing events identified by whole genome sequencing in the ω‐ and γ‐gliadin gene clusters. (a, b) The distribution of the depth of read coverage, the types of editing events, and gRNA target sites with 0, 1, 2 or 3 mismatches to the gRNA protospacers for the ω‐gliadin genes with the deletion of a cluster of three genes (a) and short deletions in the coding region (b). (c) Distribution of gRNA target sites and different types of gene editing events among the ω‐ and γ‐gliadin genes on chromosomes 1A, 1B and 1D.

**Figure 3:**
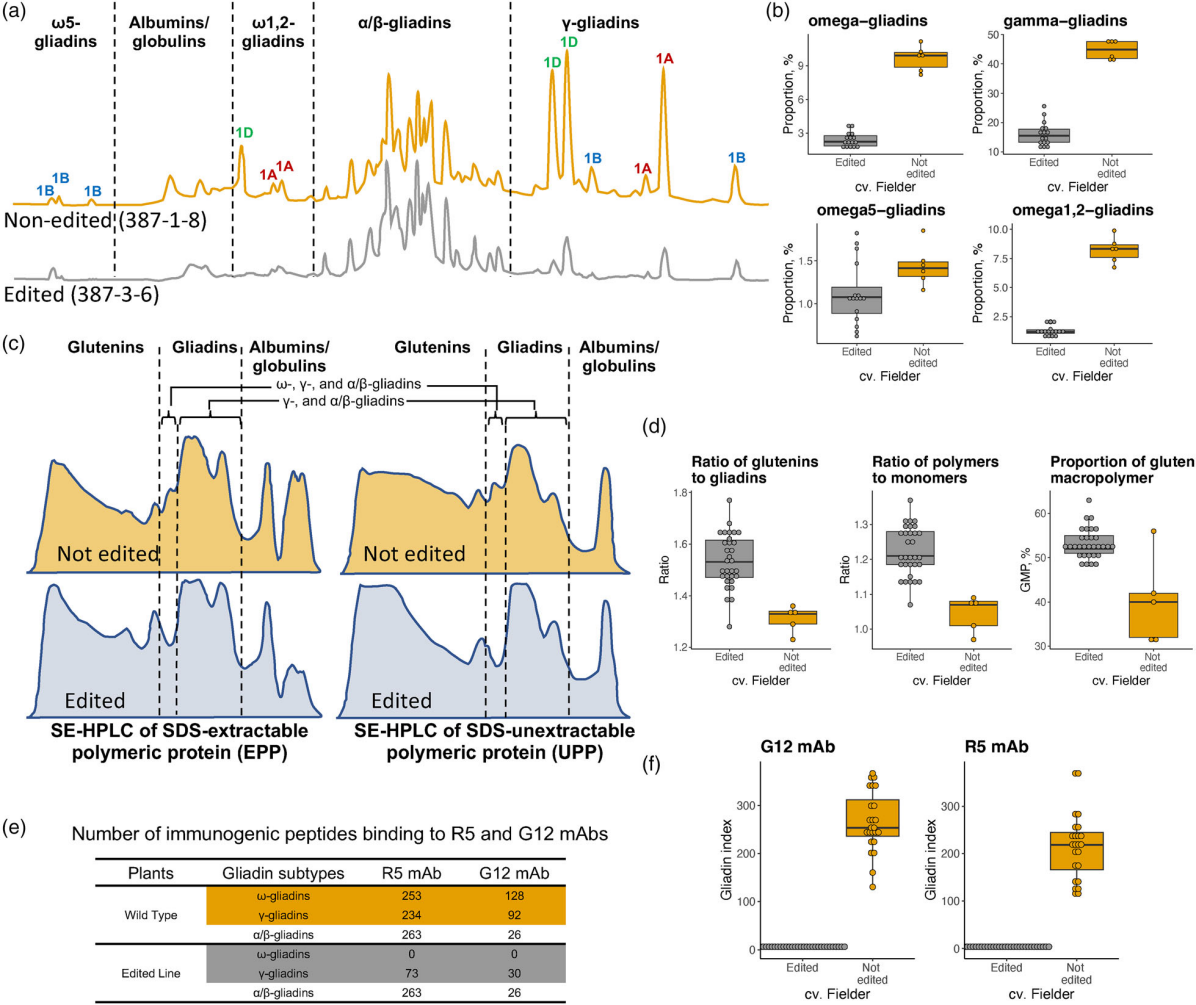
The impact of gene editing on gliadin content, properties of grain protein and immunoreactivity. (a) RP‐HPLC‐based gliadin profiles of a non‐edited transgenic line and a T_2_ plant derived from the edited line 387‐3‐6. Based on the hydrophobicity of each component (five areas separated by the black dotted lines) were assigned to ω5‐gliadins, albumins/globulins, ω1,2‐gliadins, α/β‐gliadins and γ‐gliadins. The peaks encoded by the genes located on chromosomes 1A, 1B and 1D are labelled using red, blue and green fonts. (b) Boxplots show the proportion of ω‐ and γ‐gliadin subtypes in the gliadin extracts from the non‐edited transgenic lines and edited lines. The individual datapoints for six non‐edited transgenic lines and 16 randomly selected edited lines are shown. (c) SE‐HPLC‐based SDS‐extractable (left) and SDS‐unextractable (right) polymeric protein profiles of the non‐edited transgenic lines and two randomly selected T_2_ plants derived from the edited line 387‐3‐6. Based on the molecular weight of each component, three areas separated by black dotted lines were assigned to glutenins, gliadins and albumins/globulins. (d) Comparisons of glu/gli ratio, poly/mono ratio and GMP % between the non‐edited transgenic lines and the edited lines. The datapoints are shown for five non‐edited transgenic lines and 62 randomly selected edited lines derived from T_1_ plant 387‐3‐6. (e) Comparison of the number of immunogenic peptides binding to the R5 and G12 mAbs in cv. Fielder and gene‐edited line 387‐3‐6. The number of toxic epitopes in the gene‐edited line is estimated using the intact coding regions of gliadin genes identified by whole genome sequencing. (f) Comparison of immunoreactivity between the non‐edited transgenic lines and the edited line measured using R5 and G12 mAbs. The datapoints are shown for 24 non‐edited transgenic lines and 30 randomly selected edited lines derived from T_1_ plant 387‐3‐6. Gliadin index corresponds to gliadins × 10^4^ ppm. In the figure, boxplots show the mean value and interquartile ranges (IQR); the line inside the box indicates the mean value; the end of the top line was the third quartile (Q3) + 1.5 × IQR; the end of the bottom line was the first quartile (Q1) − 1.5 × IQR.

The identification of the edited ω‐ and γ‐gliadin gene copies (Tables S6 and S7) was based on the changes in the relative depth of read coverage between the edited and non‐edited lines, gaps in the alignments of assembled gliadin genes, and the presence of short indels at the gRNA binding sites (see Materials and methods). We detected one large‐scale deletion on chromosomes 1A that resulted in the loss of two functional ω‐gliadin genes (Figure 2), indicating that targeted removal of entire gliadin gene clusters would be feasible in wheat using the gene‐editing approach. The smaller scale deletions affecting either portion of the genes flanked by the gRNA binding sites or the regions at the gRNA binding sites were detected in the remaining functional copies of the ω‐gliadin genes and 12 functional copies of the γ‐gliadin genes (Figure 2). These results are consistent with the observed reduction of ω‐ and γ‐gliadin content in flour protein extracts (Figure 3b). The loss of gliadin genes due to editing resulted in the removal of 55.2% and 77.2% of immunotoxic peptides binding to R5 and G12 mAbs identified in the Fielder genome (Figure 3e). The analysis of potential off‐target editing sites that have up to three mismatches within the designed gRNAs revealed a lack of Cas9‐induced mutations outside of the gliadin genes, indicating that the developed line does not carry undesirable modifications in the genome.

### The impact of gene editing on gliadin content and properties of grain protein

Reverse‐phase high‐performance liquid chromatography (RP‐HPLC) technology was used to compare changes in the amount of each gliadin subtype in the progeny of the edited and non‐edited lines. First, we compared the RP‐HPLC protein profiles between a non‐edited transgenic line and wild‐type cultivar Fielder. The profiles generated for both lines had the identical number of peaks (Figure S5a), indicating that the tissue culture used for the regeneration of transgenic plants did not induce significant changes in the gliadin protein profiles. Therefore, for further analyses, we used non‐edited transgenic lines as controls instead of wild‐type cultivar Fielder. The RP‐HPLC protein profiles were compared between the progeny of the edited plants, including lines 387‐3‐6, 387‐3‐1, 387‐3‐2, 387‐3‐3, 387‐3‐4, 387‐3‐5, and 387‐3‐7, and the non‐edited transgenic lines derived from lines 387‐1‐8. Compared to the non‐edited controls, the edited line 387‐3‐6 showed the highest levels of reduction in all gliadin subtypes, except for ω5‐gliadins, which showed the higher level of reduction in line 387‐3‐1 (Table S8). Afterwards, we compared the amount of ω‐ and γ‐gliadins between the T_2_ plants of edited line 387‐3‐6 and the non‐edited control (Figure 3a). The analysis revealed a statistically significant decrease in ω‐ and γ‐gliadins in the edited line 387‐3‐6. The total amount of ω‐gliadins was decreased by 75.3% (*t*‐test; *P*‐value = 9.14E‐08; Figure 3b), with ω1,2‐gliadins and ω5‐gliadins decreased by 84.6% and 22.3%, respectively (*t*‐test; *P*‐value = 8.67E‐06 and 2.84E‐02; Figure 3b). The amount of γ‐gliadin protein fraction was decreased by 63.9% (*t*‐test; *P*‐value = 2.10E‐09; Figure 3b). These results confirm that the proportion of ω‐ and γ‐gliadins in line 387‐3‐6 is substantially reduced by the CRISPR‐Cas9 mediated gene editing.

To assess the impact of gene editing on the breadmaking properties of dough, we have analysed the SDS‐extractable polymeric proteins (EPP) and SDS‐unextractable polymeric proteins (UPP) using size‐exclusion HPLC (SE‐HPLC; Ohm *et al*., 2009a, 2009b). The protein profiles generated by SE‐HPLC for EPP (Figure S5b) and UPP (Figure S5c) for a non‐edited transgenic line and wild‐type cultivar Fielder were similar, again confirming that regeneration of transgenic plants through tissue culture did not impact gliadin composition. Hence, non‐edited transgenic lines were used as controls for assessing the impact of gene editing on the SE‐HPLC protein profiles.

Compared to non‐edited transgenic lines, the first peak corresponding to gliadins in the profiles of EPP and UPP was missing in edited line 387‐3‐6 (Figure 3c). The SE‐HPLC data was used to calculate three parameters of protein extracts that strongly correlate with breadmaking quality: the ratio of glutenins to gliadins (glu/gli), the ratio of polymeric to monomeric proteins (poly/mono) and the percentage of GMP (GMP %). The statistically significant increases in all three investigated parameters were observed in edited lines relative to non‐edited transgenic lines (Figure 3d). The glu/gli and poly/mono ratios increased from 1.31 to 1.53 (*t*‐test; *P*‐value = 1.68E‐05) and from 1.05 to 1.22 (*t*‐test; *P*‐value = 2.48E‐04), respectively. There was a statistically significant increase in GMP % from 40.1% to 53.2% (*t*‐test; *P*‐value = 4.10E‐02). These results suggest that a decrease in the ω‐ and γ‐gliadin content in the developed gene‐edited line facilitates gluten polymerization and improves the parameters of flour protein extracts correlating with breadmaking quality. This conclusion is supported by the results of dough quality tests performed using Mixograph and Micro‐Farinograph (Table S9).

The dough quality tests on Mixograph and Micro‐Farinograph were conducted using white flour prepared from the non‐edited transgenic lines and edited line 387‐3‐6. The Mixograph analysis showed a substantial increase in the peak time, the time required for dough development after adding water to flour, from 2.5 to 6.0 min (Figure S6). There was only a slight, statistically non‐significant decrease in mixing tolerance score from 3.3 in the non‐edited transgenic line to 2.7 in the edited line, indicating that gliadin content reduction in the edited line had little impact on the dough's consistency during mixing. The Micro‐Farinograph analyses showed a substantial increase in the stability time from 2.5 to 9.4 min, indicating the dough made from the flour of the edited line could maintain the maximum consistency for a longer time than dough made from the non‐edited transgenic line. Although other parameters in the Micro‐Farinograph analyses did not show statistically significant differences between the non‐edited and edited lines, they clearly reveal a trend towards improved gluten strength in the edited line. Overall, the results of the Mixograph and Micro‐Farinograph analyses are consistent with the increase in the proportion of GMP in the edited line (Figure 3d) and indicate that the novel gliadin gene alleles generated using the CRISPR technology have the potential to improve end‐use quality traits in wheat.

### The comparison of immunoreactivity between the non‐edited and edited lines

The mAbs R5 and G12 raised against secalins and gliadins are highly predictive of wheat immunotoxicity for gluten‐sensitive patients (Morón *et al*., 2008; Valdés *et al*., 2003). We have used 24 non‐edited lines derived from the Cas9‐negative tillers of transgenic plant 387 and 30 edited lines derived from 387 to 3‐6 to assess the immunoreactivity of gliadin extracts against both the R5 and G12 mAbs. There was a 47‐fold reduction (97.9%) in the average immunoreactivity of gliadins against R5 mAb in the edited lines (4.58 × 10^4^ ppm) compared to that in the controls (214.83 × 10^4^ ppm) (*t*‐test; *P*‐value = 3.44E‐13; Figure 3e,f).

The immunoreactivity against G12 mAb was reduced from 266.65 × 10^4^ ppm in control lines to 6.29 × 10^4^ ppm in the edited lines, which corresponds to a 42‐fold reduction (97.6%) (*t*‐test; *P*‐value = 3.68E‐16; Figure 3e,f). So, here the results show that the immunoreactivity of gene edited line has a substantial reduction around 97% at up to 42‐fold compared to non‐edited transgenic line. Using Total Gluten assay R7041 (Lacorn *et al*., 2019), which can detect nearly all gluten proteins in wheat (gliadins, HMW‐ and LMW‐GS), we detected approximately 30% immunoreactivity reduction in the edited lines (*t*‐test; *P*‐value = 9.81E‐7) compared to non‐edited transgenic line. This result suggests that the edited immunotoxic peptides from the gliadin genes in line 387‐3‐6 account for about a third of total gluten immunoreactivity detectable by the R7041 assay.

## Discussion

Due to the complexity of the wheat genome, targeted editing of multigene families in cultivars that have significant structural differences from the available reference genomes represents a significant challenge. Our study demonstrates that new genomic resources could be combined with a multiplex editing approach for engineering multigene clusters in the wheat genome. The high‐quality genome assemblies, including the genome of transformation amenable cv. Fielder, generated using long‐read sequencing technologies (Athiyannan *et al*., 2022; Sato *et al*., 2021; Walkowiak *et al*., 2020; Zhu *et al*., 2021) provided accurate annotation of the highly repetitive gliadin gene families. These resources helped to construct a map of immunotoxic peptide motif distribution across all three gliadin subtypes in the Fielder genome and facilitated designing gRNAs for targeted modification of highly immunoreactive ω‐and γ‐gliadin gene copies, without affecting the α/β‐gliadins genes.

Previously, characterization of mutations in the gliadin genes was performed by targeted capture (Jouanin *et al*., 2019) or targeted amplification (Sánchez‐León *et al*., 2018). While these approaches proved to be effective for detecting the presence of edited alleles, they had reduced ability to discern which of the specific gene copies was modified and to accurately assess off‐target editing activities. These issues have been exacerbated by the lack of reference genomes for wheat cultivars subjected to CRISPR‐based editing. Our study took advantage of the recently released Fielder genome (Sato *et al*., 2021) and applied comparative whole genome‐sequencing of the edited wheat line and a non‐edited transgenic line to create a map of editing events across the gliadin gene copies. The whole‐genome analysis revealed the lack of Cas9‐containing transgenes and the off‐target editing events elsewhere in the genome. The absence of the CRISPR‐induced mutations in the α/β‐gliadins genes indicates that the designed gRNAs were highly effective in targeting only ω‐and γ‐gliadin gene copies. The whole‐genome sequencing facilitated the detection of a broad range of CRISPR‐induced mutation types ranging from small‐ to mid‐scale deletions within individual genes to a large‐scale deletion affecting several gene copies. A large‐scale deletion on chromosomes 1A led to the loss of two functional ω‐gliadin genes, indicating that targeted removal of large multigene clusters is feasible in wheat (Jouanin *et al*., 2020) by designing gRNA guides flanking large‐gene clusters. This result indicates that CRISPR editing is a flexible tool for engineering gluten gene loci and could be used for their removal and replacement with either natural or synthetic variants of genes exhibiting lower immunoreactivity and improved quality. Overall, a significant drop in the cost of next‐generation sequencing provides an opportunity to sequence whole genomes of edited wheat lines and characterize the full range of CRISPR‐induced variation across the entire genome.

In the previous study, it was shown that CRISPR‐Cas9 editing of cv. Bobwhite by preferentially targeting α/β‐gliadin genes results in an increased proportion of glutenin relative to gliadins (Sánchez‐León *et al*., 2018). However, this was accompanied by a significant reduction in the amount of GMP measured using SDS sedimentation approach (Sánchez‐León *et al*., 2018), suggestive of reduced breadmaking quality. It is likely that the copies of the α/β‐gliadin genes edited in that study played an important role in forming the gluten network. By developing a novel gene‐edited line that retains all α/β‐gliadin gene copies intact, we had an opportunity to assess the impact of modified ω‐ and γ‐gliadin genes on the end‐use quality characteristics of flour. Contrary to results obtained for lines with the edited α/β‐gliadin genes, the percentage of GMP, which positively correlates with breadmaking quality, was significantly increased in edited line 387‐3‐6 compared to non‐edited transgenic lines. Substantial increase in the ratio of polymer to monomer in line 387‐3‐6 also indicates that the deletion of ω‐ and γ‐gliadin gene copies likely had a minor negative impact on gluten polymerization. The results of dough quality testing performed using Mixograph and Micro‐Farinograph showed improved dough strength and consistency in the edited line compared to non‐edited transgenic lines. These results suggest that targeted editing of most of the ω‐gliadin genes and a fraction of the γ‐gliadin genes in cv. Fielder had a positive impact on the properties of wheat gluten defining the breadmaking quality of wheat flour.

The R5 and G12 mAbs raised against immunoreactive peptides from secalins and gliadins are predictive of wheat immunotoxicity for gluten‐sensitive patients (Morón *et al*., 2008; Valdés *et al*., 2003). The gluten immunoreactivity to G12 and R5 mAbs in our gene‐edited lines was similar to that previously reported for gene‐edited cultivar Bobwhite (Sánchez‐León *et al*., 2018). However, the reduction of immunoreactivity in our study compared to the non‐edited Fielder line was much more substantial (up to 47‐fold) than that reported for wild‐type and edited Bobwhite (2.6‐fold). This difference is mainly associated with the introgression of 1R chromosomal segment from rye into chromosome 1B of cultivar Bobwhite. This introgression resulted in the loss of gliadin‐ and glutenin‐encoding genes located on chromosome arm 1BS (Table S10), leading to lower immunoreactivity of cv. Bobwhite compared to cv. Fielder. The level of immunoreactivity reduction to G12 and R5 mAbs observed in our study was proportional to the number of immunoreactive peptides lost due to editing ω‐and γ‐gliadin gene clusters in cv. Fielder, suggests that these mAbs primarily recognize epitopes located in these two gliadin gene families. The edited gliadin gene copies accounted for ~30% of total gluten immunoreactivity detectable using a mix of antibodies (Lacorn *et al*., 2019) capable of detecting most gluten proteins. While this level of reduction in immunoreactivity does not qualify the developed line to be safe for gluten‐sensitive individuals, our study significantly improves our understanding of the impact that different gliadin gene families have on immunogenic and breadmaking characteristics of wheat flour and offers potential strategies for their improvement.

Arguably, one of the potential benefits of reducing the immunotoxicity of wheat, which will likely never reach the level to be safe for patients with strong response, is diversification of wheat‐based product options that are more tolerable for people with mild gluten‐related issues, or those who are concerned about developing sensitivity to gluten and would like to reduce its intake. However, the development of hypoimmunoreactive varieties using conventional breeding is complicated due to the lack of natural gluten gene loci in wheat and its wild relatives that carry only low‐toxicity epitopes. The gene editing technologies combined with new genomic resources provide unique opportunities for targeted modification of gluten genes enriched for immunoreactive peptides. The improved protein quality and reduced immunotoxicity of the edited wheat line developed here suggest that it could be directly incorporated into breeding programs as a source of new gliadin gene alleles. In addition, our study demonstrates that gene editing could be also applied to remove large gliadin gene clusters, which is an important step towards modifying natural gluten gene loci by replacing high‐toxicity with low‐toxicity alleles. In coming years, we might expect significant advances towards developing new wheat lines with engineered gluten gene clusters that have lower risk to human health and are characterized by good end‐use quality.

## Materials and methods

### Identification of gliadin genes in the genome of cultivar Fielder

A total of 17 ω‐, 361 α/β‐ and 261 γ‐gliadin genes cloned from *T. urartu* (2*n* = 14, AA), *T. monococcum* (2*n* = 14, AA), *Aegilops tauschii* (2*n* = 14, DD), *T. turgidum* (2*n* = 28, AABB) and *T. aestivum* (2*n* = 42, AABBDD) were downloaded from the NCBI database. These 639 sequences were compared with the Fielder genome (Sato *et al*., 2021) using the BLASTN program. In addition, we extrapolated gene models from the RefSeq v.2.1 of cv. Chinese Spring assembly using the Liftoff program (Shumate and Salzberg, 2021). For ω‐gliadin genes, this analysis identified five, eight and five gene copies on chromosomes 1A, 1B and 1D, respectively. The translated ω‐gliadin amino acid sequences showed that cv. Fielder has two functional and three pseudogene copies on chromosome 1A, two functional copies and six pseudogenes on chromosome 1B, and five functional copies on chromosome 1D. For α/β‐gliadin genes, there were, respectively, 11, 27 and 14 copies on chromosomes 6A, 6B and 6D. The translated amino acid sequences suggest that 10 of 11 copies, 15 of 27 copies and nine of 14 copies are functional in chromosomes 6A, 6B and 6D, respectively. For γ‐gliadin genes, we detected seven, seven and nine copies on chromosomes 1A, 1B and 1D, respectively. The translated amino acid sequences suggest that five of seven copies are functional on both chromosomes 1A and 1B, and eight of nine copies are functional on chromosome 1D (Table S6).

### CRISPR‐Cas9 mediated ω‐ and γ‐gliadin gene editing in Fielder

The sequence of nine functional ω‐gliadin genes was analysed using the web‐tool CRISPOR (http://crispor.tefor.net/; Concordet and Haeussler, 2018). The gRNAs targeting the repetitive regions within both the individual genes and across different gene copies were selected for the synthesis. Each gRNA was synthesized as two complementary oligonucleotides with four nucleotide overhangs at both termini by Integrated DNA Technologies (IDT, Coralville, IA). The oligonucleotides were annealed and subcloned into the Csy4‐zCas9 vector plasmid (pCas9Pvcsg) using the Golden Gate reaction with restriction endonuclease BsaI‐HF v2 (New England BioLabs, Ipswich, MA, catalogue #R3733L). The expression of Csy4, gRNA and zCas9 in plasmid pCas9Pvcsg is, respectively, driven by 35S CaMV promoter from Cauliflower Mosaic Virus, PvUbi1 promoter from switchgrass (*Panicum virgatum* L.) and ubiquitin promoter from maize (*Zea mays*). The insert of each gRNA fragment was validated by Sanger Sequencing using BigDye™ Terminator v3.1 Cycle Sequencing Kit (ThermoFisher Scientific, catalogue #4337455).

### Biolistic transformation, plant growth and screening of mutants for the fragment deletions

Seven gRNA constructs were pooled at equimolar proportions and co‐transformed with *bar*‐gene vector into the immature embryos of Fielder through biolistic transformation (Saintenac *et al*., 2013). Genomic DNA (gDNA) was isolated from the leaves of each tiller of the transgenic plants to test for the presence of Cas9 and gRNA fragments, as well as identify the mutants with targeted fragment deletions. Briefly, leaf tissues were collected and homogenized in 400 μL of TPS buffer (100 mM Tris–HCl, 10 mM EDTA, 1 M KCl, pH8.0) using TissueLyser II (Qiagen, Beverly MA, USA), followed by incubation for 20 min at 75°C. After centrifugation, 130 μL of the supernatant was mixed with 130 μL of 100% isopropanol and incubated for 30 min at room temperature. DNA was precipitated, rinsed with 70% ethanol and dissolved in 150 μL of double distilled water (ddH_2_O). Plants were grown in a 1 L square pot filled with three parts of soil mixture including soil, peat moss, perlites and CaSO_4_ at a volume ratio of 20:20:10:1, covered by one part of SunGro soil, and arranged according to the completely randomized design in the greenhouse of Kansas State University under 18 h light and 6 h dark with day and night temperature set to 25 and 20°C, respectively (Wang *et al*., 2018a).

The presence of Cas9 and gRNA fragments in T_0_ plants was validated by PCR amplification using primers listed in Table S3. The primer pair 1A1DmiseqF‐1&1A1DmiseqR‐4 are designed to amplify 791‐bp fragment from FD1A_omega1, 872‐bp fragment from FD1A_omega3, 830‐bp fragment from FD1D_omega1, and 854‐bp fragment from other four gene copies in chromosome 1D. The amplified fragments include two targeted regions in FD1A_omega1 and FD1A_omega3, seven regions in FD1D_omega1, and six regions in the other four gene copies of chromosome 1D. The primer pairs FD1B114miseqF‐3&FD1B1216miseqR‐1 and FD1B114miseqF‐3&FD1B1216miseqR‐2 are designed to amplify 1303‐bp fragments from FD1B_omega3 and 1322‐bp from FD1B_omega5, respectively (Table S4).

The PCR reaction was performed using Taq DNA Polymerase (Bullseye Taq DNA Polymerase, 1000 Units, MidSci, SKU number: BETAQ‐1000) with a reaction volume of 15 μL including 1.5 μL 10× Taq buffer, 0.8 μL 2 mM dNTP, 0.9 μL 25 mM MgCl_2,_ 0.2 μL Taq polymerase, 1 μL 5 pmol primer mix, 1 μL gDNA template and 9.6 μL ddH_2_O. The dropdown program was used for PCR setting including the initial denaturation at 94°C for 4 min, 5 cycles of 94°C for 15 s, 65°C for 30 s and 72°C for 20 s, 5 cycles of 94°C for 15 s, 60°C for 30 s and 72°C for 20 s, followed by 20 cycles of 94°C for 15 s, 55°C for 30 s and 72°C for 20 s, ended with 72°C for 5 min. The amplified products were checked by running the 2% agarose gel. The fragment deletion events of T_1_ plants were examined by PCR amplification using the primers listed in Table S3. The PCR reaction was performed using NEBNext^®^ High‐Fidelity 2× PCR Master Mix (New England BioLabs, catalogue #M0541) with a reaction volume of 10 ul including 5 μL 2× PCR Master Mix, 1 μL 5 pmol primer mix, 1 μL DNA template and 3 μL ddH2O. The dropdown program was used for PCR setting including the initial denaturation at 98°C for 2 min, 5 cycles of 98°C for 10 s, 65°C for 30 s and 72°C for 40 s or 1 min, 5 cycles of 98°C for 10 s, 60°C for 30 s and 72°C for 40 s or 1 min, 20 cycles of 98°C for 10 s, 55°C for 30 s and 72°C for 40 s or 1 min, ended with 72°C for 5 min. The amplified products were checked by running the 1.5% agarose gel.

### Next‐generation sequencing of PCR amplicons

Next‐generation sequencing (NGS) of PCR amplicons was also used for detecting the gene editing events in the ω‐gliadin genes (Wang *et al*., 2018b). The genomic regions harbouring the targeted regions were amplified using primer pairs 1A1DmiseqF‐1&1A1DmiseqR‐1 and 1BmiseqF‐2&1BmiseqR‐2 carrying the tails for the second round of PCR. The second round of PCR was used to finalize the reconstruction of Illumina Truseq adaptors and add barcodes for multiplexed NGS analysis of multiple genomic regions (Table S3). All reads passing quality control were aligned against their corresponding reference sequence from wild‐type Fielder genome. The editing events were visualized using Unipro UGENE v38.1 (Okonechnikov *et al*., 2012).

### Characterization of a gene‐edited and non‐edited transgenic lines by whole‐genome sequencing

Genomic libraries for Illumina sequencing were constructed from ~2 μg of genomic DNA using the PCR‐free Illumina protocol at the K‐State Integrated Genomics Facility (IGF). The libraries were subjected to size selection using the Pippin Prep system (Sage Scientific) to enrich for 400‐600 bp fragments. The 2 × 150‐bp Illumina reads generated for edited line 387‐3‐6 (539 409 307 read pairs) and non‐edited transgenic line 387‐1‐8 (1 223 003 373 read pairs) have been mapped to the Fielder genome using BWA‐MEM with the default settings. The analyses of the depth of read coverage were performed using two alignment files: (1) a file that includes all mapped reads, and (2) a file that includes reads filtered to remove those that have more than one mismatch, mapping quality below 20 and overlapping length more than 80 bp. The depth of read coverage per base in both genomes was calculated using samtools. The genome sequence data for the edited line is accessible at NCBI BioProject PRJNA1039537.

The variant calling procedure followed the best practices suggested for the GATK v.4.0 software with the default settings and was performed using the alignment file with the uniquely mapped reads. The 2 389 466 raw variants identified by GATK were filtered to remove false positives using the following parameters: variants should be homozygous and covered by reads with mapping quality >20 at the depths >4 reads and <15 reads. In total, 1723 SNPs were identified that differentiated the edited line from the non‐edited transgenic line. Only 29 of these variants were located within the gene coding regions, indicating that both sequenced lines have nearly identical genetic backgrounds.

Before analysing gene editing events, we have identified potential sites targeted by gRNAs within the gliadin‐encoding genes. For this purpose, the target sites for the designed gRNAs were located within the Fielder genome using ‘seqkit locate’ command with up to three mismatches allowed. The gRNA target sites were classified into two groups: high confidence (HC) and low confidence (LC). The HC group included target sites that had no mismatches with the gRNAs or had two or fewer mismatches within a region more than 10‐bp away from the PAM. The LC group included sites not included in high confidence set.

We expected that the presence of multiple gRNA target sites designed to the repetitive motifs within the gliadin genes would result in small‐ and large‐scale deletions either within the gene cluster or within individual genes. These deletions could be detected either based on the drop of read coverage at the gRNA binding sites if editing affected only small portion of the gene or based on the drop of read coverage within the coding region if editing resulted in the deletion of an entire gene or substantial part of the gene. To detect the edited gliadin gene copies, we have used the ratio of the depth of read coverage within 50‐bp windows in the genomes of edited line 387‐3‐6 and non‐edited transgenic line 387‐1‐8. The ratio was calculated for every gene to build genome‐wide distribution. The 95th percentile of coverage ratio calculated for 50 bp windows was 4.78 in a dataset based on all reads mapped using default settings, and 5.55 in the filtered dataset (see above for description of both datasets).

In addition, we have analysed aligned reads for the presence of small‐scale deletions in the read alignments near the PAM at the gRNA binding sites. To validate these deletions, we have also re‐assembled reads aligned to the gliadin genes and aligned assembled contigs to the Fielder genome. This approach allowed us to detect gene edits even in the cases when no significant drop in read coverage was observed. By combining information from GATK, indel analysis and depth of read coverage, we have identified gene editing events within the gliadin gene clusters.

We have also analysed lines 387‐3‐6 for the presence of off‐target gene editing events. For this analysis, we have used sites outside of the gliadin genes aligning to gRNAs with 1, 2 or 3 mismatches. Then we checked for overlap of these sites with the indels detected by GATK.

### Extraction of gliadins, SDS‐extractable and SDS‐unextractable polymeric proteins

Gliadins were isolated from the half seeds with endosperm from the edited lines, non‐edited transgenic lines, and wild‐type Fielder. The half seeds were ground into wholemeal flour using TissueLyser II, followed by adding 1 mL of 70% ethanol (Yu *et al*., 2021). After shaking at room temperature for 1 h and centrifuging at 15 000 *g* for 15 min, the supernatant containing soluble gliadin fraction was collected.

SDS‐extractable and SDS‐unextractable polymeric proteins were also extracted from the half seeds with endosperm. Two half seeds were ground together using TissueLyser II to obtain 30 mg wholemeal flour for each sample, followed by adding 1.5 mL of 0.05 M phosphate‐buffered saline buffer (PBS, pH 6.9) with 0.05% sodium dodecyl sulphate (SDS; Sigma‐Aldrich). After shaking at room temperature for 1 h, the supernatant was collected as SDS‐extractable polymeric protein (EPP) fraction by centrifuging at 15 000 *g* for 15 min. Another 0.6 mL of 0.05 M PBS extraction buffer with 0.05% SDS was added to the pellets for isolating SDS‐unextractable polymeric proteins (UPP). The pellets were suspended in the extraction buffer, followed by sonication using a probe sonication instrument at 10 W for 3 min. After shaking for 30 min, the supernatant was collected as UPP by centrifuge at 15 000 *g* for 15 min (Batey *et al*., 1991).

### Characterization of gliadins, SDS‐extractable and SDS‐unextractable polymeric proteins using high‐performance liquid chromatograph

The characterization of gliadins was performed by reverse‐phase high‐performance liquid chromatograph (RP‐HPLC) using an Agilent 1290 Infinity II LC system (Agilent Technologies, http://www.agilent.com). Twenty microliters of the gliadin extracts were injected into a C18 reversed‐phase Zorbax 300 StableBond column (4.6 × 250 mm, 5 μm, 300 Å, Agilent Technologies), maintained at 60°C. The eluents were ultrapure water (solvent A) and acetonitrile (ACN, LC–MS Grade, Thermo Scientific, Catalogue #A955‐4, solvent B), each containing 0.06% Trifluoroacetic Acid (TFA, LC–MS Grade; Thermo Scientific, Catalogue #85183). The flow rate was set at 1 mL/min. Gliadins was characterized by a linear gradient from 21% to 48% of solvent B in 55 min and detected by UV absorbance at 214 nm. After each run, the column was balanced for 15 min.

The characterization of EPP and UPP was performed by size‐exclusion high‐performance liquid chromatograph (SE‐HPLC) using an Agilent 1290 Infinity II LC system (Agilent Technologies, http://www.agilent.com). Twenty microliters of the EPP and UPP extracts were separately injected into a Bio SEC‐5 column (4.6 × 300 mm, 500 Å; Agilent Technologies), maintained at 25°C. The eluents were ultrapure water (solvent A) and ACN (LC–MS grade, solvent B), each containing 0.1% TFA (LC–MS grade). The flow rate was set at 0.35 mL min^−1^. The EPP and UPP were characterized using a constant gradient with 50% of solvent A and 50% of solvent B in 20 min and detected by UV absorbance at 214 nm.

### Quantification of gliadin subtypes, components of SDS‐extractable and SDS‐unextractable polymeric proteins

Chromatograms were managed by OpenLAB CDS ChemStation Edition for LC & LC/MS Systems v2.18.18 (Agilent Technologies). Based on hydrophobicity, three obvious boundary areas corresponding to ω‐gliadins, α/β‐gliadins and γ‐gliadins could be identified in the gliadin chromatogram. There were three separated areas within ω‐gliadins, sequentially assigned as ω5‐gliadins, albumins/globulins and ω1,2‐gliadins. The amount of each gliadin subtype was calculated as the area under the chromatogram trace of each component. Thus, the percentage of ω5‐, ω1,2‐, ω‐ and γ‐gliadins were calculated as the area under each component, respectively, divided by the total area of gliadins without the area under albumins/globulins.

The peaks in the chromatograms of both SDS‐extractable and SDS‐unextractable polymeric proteins corresponding to glutenins, gliadins, and albumins/globulins are shown in Figure 3c. Calculation of the amount of each component was performed based on the area‐under‐the‐peak method. Thus, the ratio of glutenins to gliadins was calculated as the glutenins in both EPP and UPP divided by the gliadins in both EPP and UPP; the ratio of polymeric to monomeric proteins was calculated as the glutenins in both EPP and UPP divided by the gliadins and albumins/globulins in both EPP and UPP; the percentage of GMP was calculated by the glutenins in UPP divided by the glutenins in both EPP and UPP.

### Dough quality tests

The dough quality tests on Mixograph and Micro‐Farinograph were conducted using 10 g of white flour from the non‐edited transgenic lines and edited line 387‐3‐6. The seeds were tempered before milling by a Quadrumat Junior experimental flour mill following the AACCI Method. The flour protein and moisture content (14%) were measured by near‐infrared reflectance analyses. The test of dough mixing properties was conducted by Mixograph according to the AACCI Method using 10 g white flour from edited line and non‐edited line (Altenbach *et al*., 2019). The test of maximum resistance of dough to extension and maximum extensibility was conducted by Micro‐Farinograph with the use of 10 g white flour from edited and non‐edited lines (Londono *et al*., 2014). Each test was performed in three biological replications.

### Enzyme‐linked immunosorbent assay

The Enzyme‐Linked Immunosorbent Assay (ELISA) was conducted using the G12 mAb kit (AgraQuant^®^ Gluten G12^®^, 10 001 994; Romer Labs, Newark, DE), the R5 mAb kit (RIDASCREEN^®^ Gliadin, Art. No. R7001; R‐Biopharm, Darmstadt, Germany) and the R‐BioPharm Total Gluten kit (RIDASCREEN^®^ Art. No. R7401). This assay contains a mixture of four mAbs: mAb R5 as well as mAbs to the HMW‐GS, LMW GSs and rye seaclins (Lacorn *et al*., 2019). The sample preparation and measurements were conducted following the manufacturer's instructions for the kits.

### Comparison of wheat 1BS and rye 1RS

A comparison in the numbers of toxic epitopes binding to R5 and G12 mAbs between gliadins in wheat 1BS and secalins in rye 1RS was made, shown as 132 vs. 95 for 1BS vs. 1RS in R5 and 54 vs. 59 for 1BS vs. 1RS in G12. Moreover, there were no orthologs of low molecular weight glutenin subunit (LMW‐GS) found in 1RS (Li *et al*., 2021). The LMW‐GS encoding genes are a cluster of genes which are tightly linked with gliadin gene loci in the short arm of chromosome 1A, 1B and 1D. The sequence similarity between LMW‐GS and gliadin gene is mainly evident in the parts that encode the repetitive domains of protein, which is the toxic epitope composed of PxQ motifs (D'Ovidio and Masci, 2004). Therefore, in comparison with Fielder, the wild‐type Bobwhite with 1BL/1RS translocation used in Sánchez‐León *et al*. (2018) is already a low gluten toxicity cultivar.

### Statistical analysis

The boxplot was used to visualize the differences in the investigated parameters between non‐edited transgenic lines and edited lines. The mean value, interquartile ranges (IQR), minimal value and the maximum value were indicated in the boxplots. The end of the top line and the bottom line of the box were the third quartile (Q3) + 1.5 × IQR and the first quartile (Q1) − 1.5 × IQR, respectively. The two‐sided *t*‐test was carried out to assess the statistical significance of the differences in the investigated parameters between non‐edited transgenic lines and edited lines.

## Author contributions

ZY—designed and conducted gene editing experiments, identified gene‐edited plants, designed and conducted experiments to characterize flour quality using HPLC, contributed to drafting the manuscript; UY—analysis of gene editing events using whole genome sequencing data; MT—conducted analyses of the immunoreactivity of the flour protein extracts; AF—contributed resources and tools for the HPLC analysis of flour quality traits; AA—generation of amplicon and whole genome next‐generation sequencing data for detecting gene editing events; HT—generation of transgenic plants for gene editing; EA—conceived idea, designed gene editing experiments, analysed and interpreted results, supervised the project and wrote the manuscript.

## Conflict of interest

The authors declared that they do not have conflict of interests.

## Supporting information


**Figure S1** The distributions of the 17 toxic epitopes including 11 binding to R5 mAb and six binding to G12 mAb across all three gliadin subtypes of cultivars Kariega, Chinese Spring and LongReach Lancer.
**Figure S2** PCR‐based screening of fragment deletions in five functional ω‐gliadin gene copies in T_1_ generation plants.
**Figure S3** PCR‐based screening of fragment deletions in five functional ω‐gliadin gene copies in T_2_ generation plants.
**Figure S4** NGS‐based detection for fragment deletions in the ω‐gliadin genes of edited line 387‐3‐6 using PCR amplicons.
**Figure S5** Protein profiles of the non‐edited transgenic line and cultivar Fielder.
**Figure S6** Mixograph curves of dough developed from the flours of non‐edited transgenic line and edited line 387‐3‐6.
**Table S1** Number of R5 and G12 mAbs binding toxic epitopes detected within the gliadin‐encoding genes from four wheat cultivars including Fielder, Kariega, Chinese Spring and LongReach Lancer.
**Table S2** Potential gRNA target sites within the gliadin genes (external Excel file).
**Table S6** Summary of gene editing events detected by whole genome sequencing in the ω‐ and γ‐gliadin gene clusters and the coordinates of gliadin genes in published Fielder genome (external Excel file).
**Table S7** The ratio of read coverage calculated by dividing the depth of read coverage in non‐edited line 387‐1‐8 to the depth of read coverage of edited line 387‐3‐6. The depth of read coverage was calculated in the 50 bp windows within each gliadin gene models (External file).
**Table S8** Raw data for generating figures to show the impacts of gene editing on the content of each gliadin subtype, parameters of protein extracts correlated with grain protein quality for breadmaking and immunoreactivity in Figure 3, and the table to show the impacts of gene editing on dough quality in Table S9 (external Excel file).
**Table S3** List of PCR primers used in the study.
**Table S4** Primers for PCR‐based screening of fragment deletions.
**Table S5** NGS‐based detection for editing events by PCR amplicon sequencing.
**Table S9** The comparison of the dough quality between the non‐edited transgenic lines and the edited line (387‐3‐6).
**Table S10** The differences in the functional storage protein gene copy number between wheat 1BS and Rye 1RS.
