# Supplementary material for: CRISPR‐based editing of the ω‐ and γ‐gliadin gene clusters reduces wheat immunoreactivity without affecting grain protein quality
Source: Plant Biotechnol J. 2023 Nov 17;22(4):892–903. doi: 10.1111/pbi.14231 (PMC10955484; doi:10.1111/pbi.14231)
Supplement: Supplementary file 1 — Figure S1 The distributions of the 17 toxic epitopes including 11 binding to R5 mAb and six binding to G12 mAb across all three gliadin subtypes of cultivars Kariega, Chinese Spring and LongReach Lancer. Figure S2 PCR‐based screening of fragment deletions in five functional ω‐gliadin gene copies in T1 generation plants. Figure S3 PCR‐based screening of fragment deletions in five functional ω‐gliadin gene copies in T2 generation plants. Figure S4 NGS‐based detection for fragment deletions in the ω‐gliadin genes of edited line 387‐3‐6 using PCR amplicons. Figure S5 Protein profiles of the non‐edited transgenic line and cultivar Fielder. Figure S6 Mixograph curves of dough developed from the flours of non‐edited transgenic line and edited line 387‐3‐6. Table S1 Number of R5 and G12 mAbs binding toxic epitopes detected within the gliadin‐encoding genes from four wheat cultivars including Fielder, Kariega, Chinese Spring and LongReach Lancer. Table S2 Potential gRNA target sites within the gliadin genes (external Excel file). Table S6 Summary of gene editing events detected by whole genome sequencing in the ω‐ and γ‐gliadin gene clusters and the coordinates of gliadin genes in published Fielder genome (external Excel file). Table S7 The ratio of read coverage calculated by dividing the depth of read coverage in non‐edited line 387‐1‐8 to the depth of read coverage of edited line 387‐3‐6. The depth of read coverage was calculated in the 50 bp windows within each gliadin gene models (External file). Table S8 Raw data for generating figures to show the impacts of gene editing on the content of each gliadin subtype, parameters of protein extracts correlated with grain protein quality for breadmaking and immunoreactivity in Figure 3, and the table to show the impacts of gene editing on dough quality in Table S9 (external Excel file). Table S3 List of PCR primers used in the study. Table S4 Primers for PCR‐based screening of fragment deletions. Table S5 NGS‐based detection for [file PBI-22-892-s001.zip › pbi14231-sup-0003-TableS7.pdf]

Supplementary Table 7. Ratio* of the w/t and 387-3-6-102 mutant reads mean depth of coverage per 50 bp windows within the ω- and γ-gliadin genes

CS2.1/NCBI gene ID	Gene feature	Chrom	Start	End	All reads			Filtered** reads		
					w/t	mut	ratio	w/t	mut	ratio
TraesCS1A03G0019200	FD1A_gamma1_functional_Intact	Chr1A	4517087	4517137	23.22	13.47	1.72	21.49	9.08	2.37
TraesCS1A03G0019200	FD1A_gamma1_functional_Intact	Chr1A	4517137	4517187	18.20	13.16	1.38	17.37	8.82	1.97
TraesCS1A03G0019200	FD1A_gamma1_functional_Intact	Chr1A	4517187	4517237	16.86	10.45	1.61	15.02	6.75	2.23
TraesCS1A03G0019200	FD1A_gamma1_functional_Intact	Chr1A	4517237	4517287	18.18	13.51	1.35	16.18	9.90	1.63
TraesCS1A03G0019200	FD1A_gamma1_functional_Intact	Chr1A	4517287	4517337	20.45	14.92	1.37	19.20	10.18	1.89
TraesCS1A03G0019200	FD1A_gamma1_functional_Intact	Chr1A	4517337	4517387	23.84	12.25	1.95	22.49	8.76	2.57
TraesCS1A03G0019200	FD1A_gamma2_functional_Intact	Chr1A	4517366	4517416	22.08	8.33	2.65	20.33	5.86	3.47
TraesCS1A03G0019200	FD1A_gamma2_functional_Intact	Chr1A	4517416	4517466	20.08	5.51	3.64	18.08	3.88	4.66
TraesCS1A03G0019200	FD1A_gamma2_functional_Intact	Chr1A	4517466	4517516	18.82	10.04	1.88	17.43	8.04	2.17
TraesCS1A03G0019200	FD1A_gamma2_functional_Intact	Chr1A	4517516	4517566	19.61	16.24	1.21	19.35	13.75	1.41
TraesCS1A03G0019200	FD1A_gamma2_functional_Intact	Chr1A	4517566	4517616	18.69	20.55	0.91	18.69	18.65	1.00
TraesCS1A03G0019200	FD1A_gamma2_functional_Intact	Chr1A	4517616	4517666	20.16	18.73	1.08	20.16	15.73	1.28
TraesCS1A03G0019200	FD1A_gamma2_functional_Intact	Chr1A	4517666	4517716	23.53	15.67	1.50	23.53	13.08	1.80
TraesCS1A03G0019200	FD1A_gamma2_functional_Intact	Chr1A	4517716	4517766	29.18	14.14	2.06	28.86	10.73	2.69
TraesCS1A03G0019200	FD1A_gamma2_functional_Intact	Chr1A	4517766	4517816	32.45	16.39	1.98	32.00	14.49	2.21
TraesCS1A03G0019200	FD1A_gamma2_functional_Intact	Chr1A	4517816	4517866	25.67	15.22	1.69	25.67	14.78	1.74
TraesCS1A03G0019200	FD1A_gamma2_functional_Intact	Chr1A	4517866	4517916	22.49	13.96	1.61	22.49	13.96	1.61
TraesCS1A03G0019200	FD1A_gamma2_functional_Intact	Chr1A	4517916	4517966	18.73	10.10	1.85	18.73	10.10	1.85
TraesCS1A03G0019200	FD1A_gamma2_functional_Intact	Chr1A	4517966	4518016	23.16	6.86	3.37	23.16	6.86	3.37
TraesCS1A03G0019200	FD1A_gamma2_functional_Intact	Chr1A	4518016	4518066	23.45	6.14	3.82	23.45	5.57	4.21
TraesCS1A03G0019300	FD1A_gamma3_partial_Intact	Chr1A	4535793	4535843	15.63	7.27	2.15	15.63	5.27	2.96
TraesCS1A03G0019300	FD1A_gamma3_partial_Intact	Chr1A	4535843	4535893	13.88	6.69	2.08	13.88	4.39	3.16
TraesCS1A03G0019300	FD1A_gamma3_partial_Intact	Chr1A	4535893	4535943	18.10	8.43	2.15	17.51	6.69	2.62
TraesCS1A03G0019300	FD1A_gamma3_partial_Intact	Chr1A	4535943	4535993	21.04	9.41	2.24	20.04	8.41	2.38
TraesCS1A03G0019300	FD1A_gamma3_partial_Intact	Chr1A	4535993	4536043	20.73	11.80	1.76	18.76	10.86	1.73
TraesCS1A03G0019300	FD1A_gamma3_partial_Intact	Chr1A	4536043	4536093	18.02	10.45	1.72	16.61	8.59	1.93
TraesCS1A03G0019300	FD1A_gamma3_partial_Intact	Chr1A	4536093	4536143	17.43	12.84	1.36	16.43	9.84	1.67
TraesCS1A03G0019300	FD1A_gamma3_partial_Intact	Chr1A	4536143	4536193	20.08	11.78	1.70	20.02	8.27	2.42
TraesCS1A03G0019300	FD1A_gamma3_partial_Intact	Chr1A	4536193	4536243	21.24	15.12	1.40	21.24	13.18	1.61
TraesCS1A03G0019300	FD1A_gamma3_partial_Intact	Chr1A	4536243	4536293	19.25	14.22	1.35	19.25	13.27	1.45
TraesCS1A03G0019300	FD1A_gamma3_partial_Intact	Chr1A	4536293	4536343	17.08	13.69	1.25	17.08	12.96	1.32
TraesCS1A03G0019300	FD1A_gamma3_partial_Intact	Chr1A	4536343	4536393	15.12	14.14	1.07	15.12	12.14	1.25
TraesCS1A03G0019300	FD1A_gamma3_partial_Intact	Chr1A	4536393	4536443	16.04	16.25	0.99	16.04	12.82	1.25
TraesCS1A03G0019300	FD1A_gamma3_partial_Intact	Chr1A	4536443	4536493	17.10	16.98	1.01	17.10	13.92	1.23
TraesCS1A03G0019300	FD1A_gamma3_partial_Intact	Chr1A	4536493	4536543	17.63	9.78	1.80	17.63	7.78	2.26
TraesCS1A03G0019300	FD1A_gamma3_partial_Intact	Chr1A	4536543	4536593	16.51	6.16	2.68	16.51	5.84	2.83
TraesCS1A03G0019300	FD1A_gamma3_partial_Intact	Chr1A	4536593	4536643	14.14	6.84	2.07	14.14	6.12	2.31
TraesCS1A03G0019300	FD1A_gamma3_partial_Intact	Chr1A	4536643	4536693	13.96	10.31	1.35	13.96	9.31	1.50
TraesCS1A03G0019600	FD1A_gamma4_functional_Deletion	Chr1A	4590580	4590630	21.31	12.90	1.65	19.31	11.90	1.62
TraesCS1A03G0019600	FD1A_gamma4_functional_Deletion	Chr1A	4590630	4590680	25.67	11.67	2.20	24.04	10.67	2.25
TraesCS1A03G0019600	FD1A_gamma4_functional_Deletion	Chr1A	4590680	4590730	23.61	9.08	2.60	23.10	8.33	2.77
TraesCS1A03G0019600	FD1A_gamma4_functional_Deletion	Chr1A	4590730	4590780	19.37	9.63	2.01	19.25	8.63	2.23
TraesCS1A03G0019600	FD1A_gamma4_functional_Deletion	Chr1A	4590780	4590830	16.61	14.45	1.15	15.61	13.45	1.16
TraesCS1A03G0019600	FD1A_gamma4_functional_Deletion	Chr1A	4590830	4590880	21.53	14.67	1.47	19.82	14.31	1.38
TraesCS1A03G0019600	FD1A_gamma4_functional_Deletion	Chr1A	4590880	4590930	27.33	13.41	2.04	24.51	12.96	1.89

TraesCS1A03G0019600	FD1A_gamma4_functional_Deletion	Chr1A	4590930	4590980	22.06	10.22	2.16	20.06	9.22	2.18
TraesCS1A03G0019600	FD1A_gamma4_functional_Deletion	Chr1A	4590980	4591030	15.88	8.63	1.84	14.24	6.86	2.07
TraesCS1A03G0019600	FD1A_gamma4_functional_Deletion	Chr1A	4591030	4591080	14.10	6.27	2.25	12.67	4.53	2.80
TraesCS1A03G0019600	FD1A_gamma4_functional_Deletion	Chr1A	4591080	4591130	15.86	8.45	1.88	14.84	5.59	2.66
TraesCS1A03G0019600	FD1A_gamma4_functional_Deletion	Chr1A	4591130	4591180	20.16	9.25	2.18	18.16	4.82	3.76
TraesCS1A03G0019600	FD1A_gamma4_functional_Deletion	Chr1A	4591180	4591230	18.31	9.65	1.90	16.98	4.43	3.83
TraesCS1A03G0019600	FD1A_gamma4_functional_Deletion	Chr1A	4591230	4591280	17.45	8.49	2.06	15.18	3.86	3.93
TraesCS1A03G0019600	FD1A_gamma4_functional_Deletion	Chr1A	4591280	4591330	20.73	6.35	3.26	17.16	3.41	5.03
TraesCS1A03G0019600	FD1A_gamma4_functional_Deletion	Chr1A	4591330	4591380	19.27	4.47	4.31	15.80	2.47	6.40
TraesCS1A03G0019600	FD1A_gamma4_functional_Deletion	Chr1A	4591380	4591430	23.73	2.39	9.92	20.71	1.25	16.50
TraesCS1A03G0019600	FD1A_gamma4_functional_Deletion	Chr1A	4591430	4591480	19.35	5.08	3.81	17.04	4.08	4.18
TraesCS1A03G0019600	FD1A_gamma4_functional_Deletion	Chr1A	4591480	4591530	19.88	6.82	2.91	18.84	6.25	3.01
TraesCS1A03G0019600	FD1A_gamma4_functional_Deletion	Chr1A	4591530	4591580	16.10	10.10	1.59	11.73	8.63	1.36
TraesCS1A03G0019800	FD1A_gamma5_pseudogene_Deletion	Chr1A	4672139	4672189	20.22	10.45	1.93	20.22	10.45	1.93
TraesCS1A03G0019800	FD1A_gamma5_pseudogene_Deletion	Chr1A	4672189	4672239	20.86	10.37	2.01	19.90	9.82	2.03
TraesCS1A03G0019800	FD1A_gamma5_pseudogene_Deletion	Chr1A	4672239	4672289	19.22	9.94	1.93	17.22	7.25	2.37
TraesCS1A03G0019800	FD1A_gamma5_pseudogene_Deletion	Chr1A	4672289	4672339	16.63	8.02	2.07	13.84	4.10	3.38
TraesCS1A03G0019800	FD1A_gamma5_pseudogene_Deletion	Chr1A	4672339	4672389	13.98	6.04	2.31	10.88	2.22	4.91
TraesCS1A03G0019800	FD1A_gamma5_pseudogene_Deletion	Chr1A	4672389	4672439	12.16	5.10	2.38	10.16	1.47	6.91
TraesCS1A03G0019800	FD1A_gamma5_pseudogene_Deletion	Chr1A	4672439	4672489	15.02	5.63	2.67	13.80	1.90	7.26
TraesCS1A03G0019800	FD1A_gamma5_pseudogene_Deletion	Chr1A	4672489	4672539	19.47	7.47	2.61	18.67	1.78	10.46
TraesCS1A03G0019800	FD1A_gamma5_pseudogene_Deletion	Chr1A	4672539	4672589	21.78	7.37	2.95	21.18	3.02	7.01
TraesCS1A03G0019800	FD1A_gamma5_pseudogene_Deletion	Chr1A	4672589	4672639	21.25	7.37	2.88	20.84	4.51	4.62
TraesCS1A03G0019800	FD1A_gamma5_pseudogene_Deletion	Chr1A	4672639	4672689	17.43	7.92	2.20	16.43	6.31	2.60
TraesCS1A03G0019800	FD1A_gamma5_pseudogene_Deletion	Chr1A	4672689	4672739	16.92	7.45	2.27	15.92	6.37	2.50
TraesCS1A03G0019800	FD1A_gamma5_pseudogene_Deletion	Chr1A	4672739	4672789	16.86	7.00	2.41	16.10	6.14	2.62
TraesCS1A03G0019800	FD1A_gamma5_pseudogene_Deletion	Chr1A	4672789	4672839	21.20	9.63	2.20	20.20	9.63	2.10
TraesCS1A03G0019800	FD1A_gamma5_pseudogene_Deletion	Chr1A	4672839	4672889	24.63	10.31	2.39	23.63	10.31	2.29
TraesCS1A03G0020500	FD1A_gamma6_functional_Deletion	Chr1A	4771825	4771875	19.00	9.29	2.04	0.00	0.00	0.00
TraesCS1A03G0020500	FD1A_gamma6_functional_Deletion	Chr1A	4771875	4771925	17.86	7.61	2.35	0.00	0.00	0.00
TraesCS1A03G0020500	FD1A_gamma6_functional_Deletion	Chr1A	4771925	4771975	16.41	10.47	1.57	0.00	0.00	0.00
TraesCS1A03G0020500	FD1A_gamma6_functional_Deletion	Chr1A	4771975	4772025	14.59	11.45	1.27	0.00	0.00	0.00
TraesCS1A03G0020500	FD1A_gamma6_functional_Deletion	Chr1A	4772025	4772075	15.27	10.78	1.42	0.00	0.00	0.00
TraesCS1A03G0020500	FD1A_gamma6_functional_Deletion	Chr1A	4772075	4772125	14.96	10.00	1.50	0.00	0.00	0.00
TraesCS1A03G0020500	FD1A_gamma6_functional_Deletion	Chr1A	4772125	4772175	20.96	8.49	2.47	1.35	0.08	17.25
TraesCS1A03G0020500	FD1A_gamma6_functional_Deletion	Chr1A	4772175	4772225	25.45	11.78	2.16	3.63	1.00	3.63
TraesCS1A03G0020500	FD1A_gamma6_functional_Deletion	Chr1A	4772225	4772275	25.45	13.33	1.91	5.27	1.02	5.17
TraesCS1A03G0020500	FD1A_gamma6_functional_Deletion	Chr1A	4772275	4772325	22.22	13.82	1.61	5.71	2.82	2.02
TraesCS1A03G0020500	FD1A_gamma6_functional_Deletion	Chr1A	4772325	4772375	22.59	11.43	1.98	6.29	4.88	1.29
TraesCS1A03G0020500	FD1A_gamma6_functional_Deletion	Chr1A	4772375	4772425	25.24	9.08	2.78	9.22	5.75	1.60
TraesCS1A03G0020500	FD1A_gamma6_functional_Deletion	Chr1A	4772425	4772475	23.94	9.75	2.46	10.29	5.76	1.79
TraesCS1A03G0020500	FD1A_gamma6_functional_Deletion	Chr1A	4772475	4772525	20.57	6.51	3.16	13.51	4.41	3.06
TraesCS1A03G0020500	FD1A_gamma6_functional_Deletion	Chr1A	4772525	4772575	20.27	7.71	2.63	17.37	5.84	2.97
TraesCS1A03G0020500	FD1A_gamma6_functional_Deletion	Chr1A	4772575	4772625	22.90	6.92	3.31	22.22	5.92	3.75
TraesCS1A03G0020500	FD1A_gamma6_functional_Deletion	Chr1A	4772625	4772675	21.63	7.45	2.90	20.76	6.53	3.18
TraesCS1A03G0020500	FD1A_gamma6_functional_Deletion	Chr1A	4772675	4772725	18.71	7.41	2.52	17.71	6.80	2.60
TraesCS1A03G0020400	FD1A_gamma7_functional_Deletion	Chr1A	4779545	4779595	11.80	5.92	1.99	0.00	0.00	0.00
TraesCS1A03G0020400	FD1A_gamma7_functional_Deletion	Chr1A	4779595	4779645	13.82	5.96	2.32	0.00	0.00	0.00
TraesCS1A03G0020400	FD1A_gamma7_functional_Deletion	Chr1A	4779645	4779695	16.61	6.29	2.64	0.00	0.00	0.00
TraesCS1A03G0020400	FD1A_gamma7_functional_Deletion	Chr1A	4779695	4779745	18.90	10.04	1.88	0.00	0.00	0.00
TraesCS1A03G0020400	FD1A_gamma7_functional_Deletion	Chr1A	4779745	4779795	21.63	9.41	2.30	0.86	0.00	0.86

TraesCS1A03G0020400	FD1A_gamma7_functional_Deletion	Chr1A	4779795	4779845	22.59	10.47	2.16	1.10	0.00	1.10
TraesCS1A03G0020400	FD1A_gamma7_functional_Deletion	Chr1A	4779845	4779895	21.96	9.20	2.39	2.90	0.00	2.90
TraesCS1A03G0020400	FD1A_gamma7_functional_Deletion	Chr1A	4779895	4779945	14.82	11.22	1.32	2.69	0.00	2.69
TraesCS1A03G0020400	FD1A_gamma7_functional_Deletion	Chr1A	4779945	4779995	15.80	9.86	1.60	4.92	1.33	3.69
TraesCS1A03G0020400	FD1A_gamma7_functional_Deletion	Chr1A	4779995	4780045	14.29	10.00	1.43	5.10	3.55	1.44
TraesCS1A03G0020400	FD1A_gamma7_functional_Deletion	Chr1A	4780045	4780095	17.94	14.41	1.24	6.37	7.80	0.82
TraesCS1A03G0020400	FD1A_gamma7_functional_Deletion	Chr1A	4780095	4780145	19.22	15.73	1.22	8.45	8.33	1.01
TraesCS1A03G0020400	FD1A_gamma7_functional_Deletion	Chr1A	4780145	4780195	19.98	14.63	1.37	11.47	6.55	1.75
TraesCS1A03G0020400	FD1A_gamma7_functional_Deletion	Chr1A	4780195	4780245	21.57	9.04	2.39	18.24	5.43	3.36
TraesCS1A03G0020400	FD1A_gamma7_functional_Deletion	Chr1A	4780245	4780295	20.49	11.29	1.81	19.55	9.14	2.14
TraesCS1A03G0020400	FD1A_gamma7_functional_Deletion	Chr1A	4780295	4780345	22.22	12.67	1.75	21.73	11.67	1.86
TraesCS1A03G0020400	FD1A_gamma7_functional_Deletion	Chr1A	4780345	4780395	20.31	12.47	1.63	19.31	11.47	1.68
TraesCS1A03G0020400	FD1A_gamma7_functional_Deletion	Chr1A	4780395	4780445	20.69	10.33	2.00	19.69	9.96	1.98
TraesCS1A03G0015200	FD1A_omega1_functional_Deletion	Chr1A	3842760	3842810	14.71	7.37	1.99	13.71	5.55	2.47
TraesCS1A03G0015200	FD1A_omega1_functional_Deletion	Chr1A	3842810	3842860	11.08	7.04	1.57	10.29	5.04	2.04
TraesCS1A03G0015200	FD1A_omega1_functional_Deletion	Chr1A	3842860	3842910	14.47	6.84	2.11	14.47	5.08	2.85
TraesCS1A03G0015200	FD1A_omega1_functional_Deletion	Chr1A	3842910	3842960	16.43	3.16	5.20	16.33	3.08	5.31
TraesCS1A03G0015200	FD1A_omega1_functional_Deletion	Chr1A	3842960	3843010	18.82	1.57	12.00	17.82	1.57	11.36
TraesCS1A03G0015200	FD1A_omega1_functional_Deletion	Chr1A	3843010	3843060	17.18	3.61	4.76	14.96	3.61	4.15
TraesCS1A03G0015200	FD1A_omega1_functional_Deletion	Chr1A	3843060	3843110	15.65	6.57	2.38	12.75	6.25	2.04
TraesCS1A03G0015200	FD1A_omega1_functional_Deletion	Chr1A	3843110	3843160	16.63	5.24	3.18	14.35	4.24	3.39
TraesCS1A03G0015200	FD1A_omega1_functional_Deletion	Chr1A	3843160	3843210	18.65	2.61	7.15	17.35	2.16	8.05
TraesCS1A03G0015200	FD1A_omega1_functional_Deletion	Chr1A	3843210	3843260	16.90	0.39	43.10	16.20	0.39	41.30
TraesCS1A03G0015200	FD1A_omega1_functional_Deletion	Chr1A	3843260	3843310	18.61	0.00	18.61	16.53	0.00	16.53
TraesCS1A03G0015200	FD1A_omega1_functional_Deletion	Chr1A	3843310	3843360	18.35	0.00	18.35	15.59	0.00	15.59
TraesCS1A03G0015200	FD1A_omega1_functional_Deletion	Chr1A	3843360	3843410	22.14	0.00	22.14	19.57	0.00	19.57
TraesCS1A03G0015200	FD1A_omega1_functional_Deletion	Chr1A	3843410	3843460	29.86	0.00	29.86	26.98	0.00	26.98
TraesCS1A03G0015200	FD1A_omega1_functional_Deletion	Chr1A	3843460	3843510	32.88	0.00	32.88	30.65	0.00	30.65
TraesCS1A03G0015200	FD1A_omega1_functional_Deletion	Chr1A	3843510	3843560	37.06	0.00	37.06	35.76	0.00	35.76
TraesCS1A03G0015200	FD1A_omega1_functional_Deletion	Chr1A	3843560	3843610	30.10	0.00	30.10	29.55	0.00	29.55
TraesCS1A03G0015200	FD1A_omega1_functional_Deletion	Chr1A	3843610	3843660	29.67	0.00	29.67	27.20	0.00	27.20
TraesCS1A03G0015200	FD1A_omega1_functional_Deletion	Chr1A	3843660	3843710	25.41	0.00	25.41	22.41	0.00	22.41
TraesCS1A03G0015200	FD1A_omega1_functional_Deletion	Chr1A	3843710	3843760	30.57	0.00	30.57	27.22	0.00	27.22
-	FD1A_omega2_pseudogene_Deletion	Chr1A	3896327	3896377	16.49	0.00	16.49	14.49	0.00	14.49
-	FD1A_omega2_pseudogene_Deletion	Chr1A	3896377	3896427	16.51	0.00	16.51	14.69	0.00	14.69
-	FD1A_omega2_pseudogene_Deletion	Chr1A	3896427	3896477	17.86	0.00	17.86	16.63	0.00	16.63
-	FD1A_omega2_pseudogene_Deletion	Chr1A	3896477	3896527	16.73	0.00	16.73	15.73	0.00	15.73
-	FD1A_omega2_pseudogene_Deletion	Chr1A	3896527	3896577	18.57	0.00	18.57	15.90	0.00	15.90
-	FD1A_omega2_pseudogene_Deletion	Chr1A	3896577	3896627	16.53	0.24	70.25	13.45	0.00	13.45
-	FD1A_omega2_pseudogene_Deletion	Chr1A	3896627	3896677	16.94	0.39	43.20	12.22	0.00	12.22
-	FD1A_omega2_pseudogene_Deletion	Chr1A	3896677	3896727	14.73	0.00	14.73	10.12	0.00	10.12
-	FD1A_omega2_pseudogene_Deletion	Chr1A	3896727	3896777	16.90	0.00	16.90	12.69	0.00	12.69
-	FD1A_omega2_pseudogene_Deletion	Chr1A	3896777	3896827	17.67	0.00	17.67	12.49	0.00	12.49
-	FD1A_omega2_pseudogene_Deletion	Chr1A	3896827	3896877	17.92	0.00	17.92	11.84	0.00	11.84
-	FD1A_omega2_pseudogene_Deletion	Chr1A	3896877	3896927	15.25	0.00	15.25	11.63	0.00	11.63
-	FD1A_omega2_pseudogene_Deletion	Chr1A	3896927	3896977	17.86	0.00	17.86	15.55	0.00	15.55
-	FD1A_omega2_pseudogene_Deletion	Chr1A	3896977	3897027	16.98	0.00	16.98	13.69	0.00	13.69
-	FD1A_omega2_pseudogene_Deletion	Chr1A	3897027	3897077	23.84	0.00	23.84	19.00	0.00	19.00
-	FD1A_omega2_pseudogene_Deletion	Chr1A	3897077	3897127	24.80	0.00	24.80	19.67	0.00	19.67
-	FD1A_omega2_pseudogene_Deletion	Chr1A	3897127	3897177	26.59	0.00	26.59	23.20	0.00	23.20
-	FD1A_omega2_pseudogene_Deletion	Chr1A	3897177	3897227	23.33	0.00	23.33	21.39	0.00	21.39

-	FD1A_omega2_pseudogene_Deletion	Chr1A	3897227	3897277	23.80	0.00	23.80	21.12	0.00	21.12
-	FD1A_omega2_pseudogene_Deletion	Chr1A	3897277	3897327	27.10	0.00	27.10	24.27	0.00	24.27
-	FD1A_omega2_pseudogene_Deletion	Chr1A	3897327	3897377	26.82	0.00	26.82	23.22	0.00	23.22
-	FD1A_omega2_pseudogene_Deletion	Chr1A	3897377	3897427	24.69	0.00	24.69	21.65	0.00	21.65
-	FD1A_omega2_pseudogene_Deletion	Chr1A	3897427	3897477	22.82	0.00	22.82	20.82	0.00	20.82
TraesCS1A03G0015300	FD1A_omega3_functional_Deletion	Chr1A	3915317	3915367	25.55	0.00	25.55	24.88	0.00	24.88
TraesCS1A03G0015300	FD1A_omega3_functional_Deletion	Chr1A	3915367	3915417	23.55	0.00	23.55	22.55	0.00	22.55
TraesCS1A03G0015300	FD1A_omega3_functional_Deletion	Chr1A	3915417	3915467	30.73	0.00	30.73	27.67	0.00	27.67
TraesCS1A03G0015300	FD1A_omega3_functional_Deletion	Chr1A	3915467	3915517	36.27	0.00	36.27	34.10	0.00	34.10
TraesCS1A03G0015300	FD1A_omega3_functional_Deletion	Chr1A	3915517	3915567	31.67	0.45	70.22	30.67	0.00	30.67
TraesCS1A03G0015300	FD1A_omega3_functional_Deletion	Chr1A	3915567	3915617	21.31	0.00	21.31	20.84	0.00	20.84
TraesCS1A03G0015300	FD1A_omega3_functional_Deletion	Chr1A	3915617	3915667	15.00	0.00	15.00	12.73	0.00	12.73
TraesCS1A03G0015300	FD1A_omega3_functional_Deletion	Chr1A	3915667	3915717	15.53	0.00	15.53	12.59	0.00	12.59
TraesCS1A03G0015300	FD1A_omega3_functional_Deletion	Chr1A	3915717	3915767	23.80	0.00	23.80	21.67	0.00	21.67
TraesCS1A03G0015300	FD1A_omega3_functional_Deletion	Chr1A	3915767	3915817	22.04	0.00	22.04	21.04	0.00	21.04
TraesCS1A03G0015300	FD1A_omega3_functional_Deletion	Chr1A	3915817	3915867	12.94	0.00	12.94	11.94	0.00	11.94
TraesCS1A03G0015300	FD1A_omega3_functional_Deletion	Chr1A	3915867	3915917	9.80	0.00	9.80	8.41	0.00	8.41
TraesCS1A03G0015300	FD1A_omega3_functional_Deletion	Chr1A	3915917	3915967	11.69	0.00	11.69	8.78	0.00	8.78
TraesCS1A03G0015300	FD1A_omega3_functional_Deletion	Chr1A	3915967	3916017	11.06	0.00	11.06	9.14	0.00	9.14
TraesCS1A03G0015300	FD1A_omega3_functional_Deletion	Chr1A	3916017	3916067	12.00	0.00	12.00	11.04	0.00	11.04
TraesCS1A03G0015300	FD1A_omega3_functional_Deletion	Chr1A	3916067	3916117	12.18	5.22	2.33	10.31	3.08	3.35
TraesCS1A03G0015300	FD1A_omega3_functional_Deletion	Chr1A	3916117	3916167	11.27	6.92	1.63	9.27	5.86	1.58
TraesCS1A03G0015300	FD1A_omega3_functional_Deletion	Chr1A	3916167	3916217	7.02	8.18	0.86	5.90	7.47	0.79
TraesCS1A03G0015300	FD1A_omega3_functional_Deletion	Chr1A	3916217	3916267	10.37	6.24	1.66	9.37	5.35	1.75
TraesCS1A03G0015300	FD1A_omega3_functional_Deletion	Chr1A	3916267	3916317	10.88	10.20	1.07	9.71	8.20	1.18
TraesCS1A03G0015300	FD1A_omega3_functional_Deletion	Chr1A	3916317	3916367	12.45	13.59	0.92	10.55	10.80	0.98
TraesCS1A03G0015300	FD1A_omega3_functional_Deletion	Chr1A	3916367	3916417	16.16	14.31	1.13	15.10	12.04	1.25
TraesCS1A03G0015300	FD1A_omega3_functional_Deletion	Chr1A	3916417	3916467	19.90	14.78	1.35	18.06	12.78	1.41
TraesCS1A03G0015300	FD1A_omega3_functional_Deletion	Chr1A	3916467	3916517	23.20	14.18	1.64	22.78	12.24	1.86
TraesCS1A03G0074600	FD1A_omega4_partial_Deletion	Chr1A	19438871	19438921	24.78	2.22	11.19	16.33	0.12	138.83
TraesCS1A03G0074600	FD1A_omega4_partial_Deletion	Chr1A	19438921	19438971	22.78	6.06	3.76	20.10	2.41	8.33
TraesCS1A03G0074600	FD1A_omega4_partial_Deletion	Chr1A	19438971	19439021	21.78	8.43	2.58	21.02	5.43	3.87
TraesCS1A03G0074600	FD1A_omega4_partial_Deletion	Chr1A	19439021	19439071	18.63	9.14	2.04	18.18	7.33	2.48
TraesCS1A03G0074600	FD1A_omega4_partial_Deletion	Chr1A	19439071	19439121	21.39	6.12	3.50	19.20	6.10	3.15
TraesCS1A03G0074600	FD1A_omega4_partial_Deletion	Chr1A	19439121	19439171	24.80	6.59	3.76	22.24	6.47	3.44
TraesCS1A03G0074600	FD1A_omega4_partial_Deletion	Chr1A	19439171	19439221	28.96	8.27	3.50	23.92	6.63	3.61
TraesCS1A03G0074600	FD1A_omega4_partial_Deletion	Chr1A	19439221	19439271	24.29	9.08	2.68	21.08	7.04	2.99
TraesCS1A03G0074600	FD1A_omega4_partial_Deletion	Chr1A	19439271	19439321	24.20	6.78	3.57	19.45	3.84	5.06
TraesCS1A03G0074600	FD1A_omega4_partial_Deletion	Chr1A	19439321	19439371	23.55	5.88	4.00	19.49	3.27	5.95
TraesCS1A03G0074600	FD1A_omega4_partial_Deletion	Chr1A	19439371	19439421	27.75	5.75	4.83	24.78	3.88	6.38
TraesCS1A03G0074600	FD1A_omega4_partial_Deletion	Chr1A	19439421	19439471	23.43	7.25	3.23	20.53	5.25	3.91
TraesCS1A03G0074600	FD1A_omega4_partial_Deletion	Chr1A	19439471	19439521	21.76	4.59	4.74	18.65	3.47	5.37
TraesCS1A03G0074600	FD1A_omega4_partial_Deletion	Chr1A	19439521	19439571	21.98	0.16	140.12	19.18	0.00	19.18
TraesCS1A03G0074600	FD1A_omega4_partial_Deletion	Chr1A	19439571	19439621	28.49	2.63	10.84	24.69	0.00	24.69
TraesCS1A03G0074600	FD1A_omega4_partial_Deletion	Chr1A	19439621	19439671	30.67	5.25	5.84	27.20	1.39	19.54
TraesCS1A03G0074600	FD1A_omega4_partial_Deletion	Chr1A	19439671	19439721	29.29	5.65	5.19	26.43	3.00	8.81
TraesCS1A03G0074600	FD1A_omega4_partial_Deletion	Chr1A	19439721	19439771	27.33	5.43	5.03	24.88	2.96	8.40
TraesCS1A03G0074600	FD1A_omega4_partial_Deletion	Chr1A	19439771	19439821	25.45	5.49	4.64	22.61	3.18	7.12
TraesCS1A03G0074600	FD1A_omega4_partial_Deletion	Chr1A	19439821	19439871	24.16	5.92	4.08	21.37	3.00	7.12
TraesCS1A03G0074600	FD1A_omega4_partial_Deletion	Chr1A	19439871	19439921	26.98	7.31	3.69	23.98	4.53	5.29
TraesCS1A03G0074600	FD1A_omega4_partial_Deletion	Chr1A	19439921	19439971	26.16	4.76	5.49	24.57	3.73	6.59

TraesCS1A03G0074600	FD1A_omega4_partial_Deletion	Chr1A	19439971	19440021	24.98	4.00	6.25	23.96	4.00	5.99
TraesCS1A03G0074600	FD1A_omega4_partial_Deletion	Chr1A	19440021	19440071	21.75	4.96	4.38	19.82	4.96	4.00
TraesCS1A03G0074600	FD1A_omega4_partial_Deletion	Chr1A	19440071	19440121	22.82	8.59	2.66	20.96	7.78	2.69
TraesCS1A03G0075700	FD1A_omega5_partial_Deletion	Chr1A	19645651	19645701	15.73	9.76	1.61	14.10	7.55	1.87
TraesCS1A03G0075700	FD1A_omega5_partial_Deletion	Chr1A	19645701	19645751	18.41	8.37	2.20	16.86	6.43	2.62
TraesCS1A03G0075700	FD1A_omega5_partial_Deletion	Chr1A	19645751	19645801	14.78	8.65	1.71	14.33	7.10	2.02
TraesCS1A03G0075700	FD1A_omega5_partial_Deletion	Chr1A	19645801	19645851	12.43	6.25	1.99	12.33	4.25	2.90
TraesCS1A03G0075700	FD1A_omega5_partial_Deletion	Chr1A	19645851	19645901	11.35	4.61	2.46	9.90	2.00	4.95
TraesCS1A03G0075700	FD1A_omega5_partial_Deletion	Chr1A	19645901	19645951	16.94	2.51	6.75	14.25	1.33	10.69
TraesCS1A03G0075700	FD1A_omega5_partial_Deletion	Chr1A	19645951	19646001	23.92	3.90	6.13	20.33	2.90	7.01
TraesCS1A03G0075700	FD1A_omega5_partial_Deletion	Chr1A	19646001	19646051	25.98	8.02	3.24	22.31	6.78	3.29
TraesCS1A03G0075700	FD1A_omega5_partial_Deletion	Chr1A	19646051	19646101	20.43	9.06	2.26	18.10	8.06	2.25
TraesCS1A03G0075700	FD1A_omega5_partial_Deletion	Chr1A	19646101	19646151	16.04	9.53	1.68	13.98	8.61	1.62
TraesCS1A03G0075700	FD1A_omega5_partial_Deletion	Chr1A	19646151	19646201	16.10	6.39	2.52	13.90	6.39	2.17
TraesCS1A03G0075700	FD1A_omega5_partial_Deletion	Chr1A	19646201	19646251	19.67	7.59	2.59	17.57	7.59	2.32
TraesCS1A03G0075700	FD1A_omega5_partial_Deletion	Chr1A	19646251	19646301	25.67	6.69	3.84	23.41	5.86	3.99
TraesCS1A03G0075700	FD1A_omega5_partial_Deletion	Chr1A	19646301	19646351	25.16	5.76	4.36	20.88	4.51	4.63
TraesCS1A03G0075700	FD1A_omega5_partial_Deletion	Chr1A	19646351	19646401	24.43	6.27	3.89	18.86	2.00	9.43
TraesCS1A03G0075700	FD1A_omega5_partial_Deletion	Chr1A	19646401	19646451	18.37	6.63	2.77	13.12	1.82	7.19
TraesCS1A03G0075700	FD1A_omega5_partial_Deletion	Chr1A	19646451	19646501	18.51	9.82	1.88	14.94	5.31	2.81
TraesCS1A03G0075700	FD1A_omega5_partial_Deletion	Chr1A	19646501	19646551	20.57	9.71	2.12	14.92	8.57	1.74
TraesCS1A03G0075700	FD1A_omega5_partial_Deletion	Chr1A	19646551	19646601	22.78	9.73	2.34	16.45	9.27	1.77
TraesCS1A03G0075700	FD1A_omega5_partial_Deletion	Chr1A	19646601	19646651	23.80	13.10	1.82	18.84	10.04	1.88
TraesCS1A03G0075700	FD1A_omega5_partial_Deletion	Chr1A	19646651	19646701	21.88	13.84	1.58	21.49	9.02	2.38
TraesCS1A03G0075700	FD1A_omega5_partial_Deletion	Chr1A	19646701	19646751	17.37	16.69	1.04	17.37	11.08	1.57
TraesCS1B03G0019400	FD1B_gamma1_functional_Deletion	Chr1B	5991451	5991501	20.63	8.12	2.54	20.55	7.04	2.92
TraesCS1B03G0019400	FD1B_gamma1_functional_Deletion	Chr1B	5991501	5991551	13.82	6.20	2.23	13.82	5.88	2.35
TraesCS1B03G0019400	FD1B_gamma1_functional_Deletion	Chr1B	5991551	5991601	11.22	5.29	2.12	11.22	4.06	2.76
TraesCS1B03G0019400	FD1B_gamma1_functional_Deletion	Chr1B	5991601	5991651	14.96	5.67	2.64	14.65	1.49	9.83
TraesCS1B03G0019400	FD1B_gamma1_functional_Deletion	Chr1B	5991651	5991701	23.24	5.84	3.98	20.39	1.31	15.52
TraesCS1B03G0019400	FD1B_gamma1_functional_Deletion	Chr1B	5991701	5991751	20.41	7.25	2.81	18.20	1.75	10.43
TraesCS1B03G0019400	FD1B_gamma1_functional_Deletion	Chr1B	5991751	5991801	12.69	5.84	2.17	12.47	2.80	4.45
TraesCS1B03G0019400	FD1B_gamma1_functional_Deletion	Chr1B	5991801	5991851	12.16	5.61	2.17	11.84	3.02	3.92
TraesCS1B03G0019400	FD1B_gamma1_functional_Deletion	Chr1B	5991851	5991901	18.16	5.25	3.46	17.16	4.24	4.05
TraesCS1B03G0019400	FD1B_gamma1_functional_Deletion	Chr1B	5991901	5991951	24.92	7.59	3.28	23.92	6.18	3.87
TraesCS1B03G0019400	FD1B_gamma1_functional_Deletion	Chr1B	5991951	5992001	20.90	12.31	1.70	20.22	11.14	1.82
TraesCS1B03G0019400	FD1B_gamma1_functional_Deletion	Chr1B	5992001	5992051	18.31	12.20	1.50	18.31	11.20	1.64
TraesCS1B03G0019400	FD1B_gamma1_functional_Deletion	Chr1B	5992051	5992101	19.80	9.75	2.03	19.80	9.45	2.10
TraesCS1B03G0019400	FD1B_gamma1_functional_Deletion	Chr1B	5992101	5992151	28.98	8.27	3.50	28.98	8.27	3.50
TraesCS1B03G0019400	FD1B_gamma1_functional_Deletion	Chr1B	5992151	5992201	32.45	11.25	2.88	32.22	11.25	2.86
TraesCS1B03G0019400	FD1B_gamma1_functional_Deletion	Chr1B	5992201	5992251	33.78	15.22	2.22	32.78	15.22	2.15
TraesCS1B03G0019400	FD1B_gamma1_functional_Deletion	Chr1B	5992251	5992301	26.24	13.18	1.99	25.24	13.04	1.94
TraesCS1B03G0019400	FD1B_gamma1_functional_Deletion	Chr1B	5992301	5992351	20.61	12.12	1.70	20.16	10.14	1.99
TraesCS1B03G0019400	FD1B_gamma1_functional_Deletion	Chr1B	5992351	5992401	18.02	12.41	1.45	18.02	10.39	1.73
TraesCS1B03G0019800	FD1B_gamma2_functional_Intact	Chr1B	6017467	6017517	30.75	12.98	2.37	29.08	11.98	2.43
TraesCS1B03G0019800	FD1B_gamma2_functional_Intact	Chr1B	6017517	6017567	24.69	12.22	2.02	22.69	10.31	2.20
TraesCS1B03G0019800	FD1B_gamma2_functional_Intact	Chr1B	6017567	6017617	19.76	11.06	1.79	18.35	9.18	2.00
TraesCS1B03G0019800	FD1B_gamma2_functional_Intact	Chr1B	6017617	6017667	14.47	12.84	1.13	14.12	11.16	1.27
TraesCS1B03G0019800	FD1B_gamma2_functional_Intact	Chr1B	6017667	6017717	14.65	11.94	1.23	14.65	9.61	1.52
TraesCS1B03G0019800	FD1B_gamma2_functional_Intact	Chr1B	6017717	6017767	17.55	11.82	1.48	16.94	8.82	1.92
TraesCS1B03G0019800	FD1B_gamma2_functional_Intact	Chr1B	6017767	6017817	15.92	9.04	1.76	14.84	6.02	2.47

TraesCS1B03G0019700	FD1B_gamma3_functional_Intact	Chr1B	6033648	6033698	15.04	8.67	1.74	14.98	7.67	1.95
TraesCS1B03G0019700	FD1B_gamma3_functional_Intact	Chr1B	6033698	6033748	13.04	7.94	1.64	12.04	6.49	1.85
TraesCS1B03G0019700	FD1B_gamma3_functional_Intact	Chr1B	6033748	6033798	15.14	5.76	2.63	13.25	3.84	3.45
TraesCS1B03G0019700	FD1B_gamma3_functional_Intact	Chr1B	6033798	6033848	17.71	6.29	2.81	15.84	1.82	8.69
TraesCS1B03G0019700	FD1B_gamma3_functional_Intact	Chr1B	6033848	6033898	15.65	8.02	1.95	13.43	2.98	4.51
TraesCS1B03G0019700	FD1B_gamma3_functional_Intact	Chr1B	6033898	6033948	13.63	9.27	1.47	10.49	4.20	2.50
TraesCS1B03G0019700	FD1B_gamma3_functional_Intact	Chr1B	6033948	6033998	10.59	7.73	1.37	7.59	4.71	1.61
TraesCS1B03G0019700	FD1B_gamma3_functional_Intact	Chr1B	6033998	6034048	11.24	8.37	1.34	8.84	6.29	1.40
TraesCS1B03G0019700	FD1B_gamma3_functional_Intact	Chr1B	6034048	6034098	11.84	10.39	1.14	10.84	7.76	1.40
TraesCS1B03G0019700	FD1B_gamma3_functional_Intact	Chr1B	6034098	6034148	15.88	11.49	1.38	14.88	9.25	1.61
TraesCS1B03G0019700	FD1B_gamma3_functional_Intact	Chr1B	6034148	6034198	18.61	10.98	1.69	18.22	9.25	1.97
TraesCS1B03G0019700	FD1B_gamma3_functional_Intact	Chr1B	6034198	6034248	19.98	12.00	1.67	19.73	10.14	1.95
TraesCS1B03G0019700	FD1B_gamma3_functional_Intact	Chr1B	6034248	6034298	19.49	13.84	1.41	18.49	11.27	1.64
TraesCS1B03G0019700	FD1B_gamma3_functional_Intact	Chr1B	6034298	6034348	23.31	12.10	1.93	22.31	10.14	2.20
TraesCS1B03G0019700	FD1B_gamma3_functional_Intact	Chr1B	6034348	6034398	22.63	8.80	2.57	21.90	7.82	2.80
TraesCS1B03G0019700	FD1B_gamma3_functional_Intact	Chr1B	6034398	6034448	21.31	6.39	3.33	21.31	5.90	3.61
TraesCS1B03G0019700	FD1B_gamma3_functional_Intact	Chr1B	6034448	6034498	16.94	6.39	2.65	16.94	4.24	4.00
TraesCS1B03G0019700	FD1B_gamma3_functional_Intact	Chr1B	6034498	6034548	14.49	6.49	2.23	14.49	3.49	4.15
TraesCS1B03G0019900	FD1B_gamma4_functional_Intact	Chr1B	6197006	6197056	30.82	5.55	5.55	28.20	4.63	6.09
TraesCS1B03G0019900	FD1B_gamma4_functional_Intact	Chr1B	6197056	6197106	25.22	10.75	2.35	22.22	6.94	3.20
TraesCS1B03G0019900	FD1B_gamma4_functional_Intact	Chr1B	6197106	6197156	23.43	10.57	2.22	20.43	5.98	3.42
TraesCS1B03G0019900	FD1B_gamma4_functional_Intact	Chr1B	6197156	6197206	19.55	11.61	1.68	19.14	7.41	2.58
TraesCS1B03G0019900	FD1B_gamma4_functional_Intact	Chr1B	6197206	6197256	20.08	10.82	1.86	18.65	8.90	2.09
TraesCS1B03G0019900	FD1B_gamma4_functional_Intact	Chr1B	6197256	6197306	17.71	16.29	1.09	16.12	13.12	1.23
TraesCS1B03G0019900	FD1B_gamma4_functional_Intact	Chr1B	6197306	6197356	15.80	19.06	0.83	13.20	13.16	1.00
TraesCS1B03G0019900	FD1B_gamma4_functional_Intact	Chr1B	6197356	6197406	18.55	16.84	1.10	15.39	11.94	1.29
TraesCS1B03G0019900	FD1B_gamma4_functional_Intact	Chr1B	6197406	6197456	18.98	11.65	1.63	15.35	9.90	1.55
TraesCS1B03G0019900	FD1B_gamma4_functional_Intact	Chr1B	6197456	6197506	17.98	7.29	2.47	15.73	6.29	2.50
TraesCS1B03G0019900	FD1B_gamma4_functional_Intact	Chr1B	6197506	6197556	15.10	5.18	2.92	13.35	4.57	2.92
TraesCS1B03G0019900	FD1B_gamma4_functional_Intact	Chr1B	6197556	6197606	14.82	8.33	1.78	14.43	7.33	1.97
TraesCS1B03G0019900	FD1B_gamma4_functional_Intact	Chr1B	6197606	6197656	15.92	10.43	1.53	15.92	9.43	1.69
TraesCS1B03G0019900	FD1B_gamma4_functional_Intact	Chr1B	6197656	6197706	18.02	9.80	1.84	17.88	9.35	1.91
TraesCS1B03G0019900	FD1B_gamma4_functional_Intact	Chr1B	6197706	6197756	22.41	5.63	3.98	21.41	5.63	3.80
TraesCS1B03G0019900	FD1B_gamma4_functional_Intact	Chr1B	6197756	6197806	24.20	5.20	4.66	23.20	5.20	4.46
TraesCS1B03G0019900	FD1B_gamma4_functional_Intact	Chr1B	6197806	6197856	26.67	5.67	4.71	26.20	5.29	4.95
TraesCS1B03G0019900	FD1B_gamma4_functional_Intact	Chr1B	6197856	6197906	24.61	7.53	3.27	24.25	6.53	3.71
TraesCS1B03G0019900	FD1B_gamma4_functional_Intact	Chr1B	6197906	6197956	23.94	6.82	3.51	22.78	5.67	4.02
TraesCS1B03G0019900	FD1B_gamma4_functional_Intact	Chr1B	6197956	6198006	20.84	7.37	2.83	18.84	5.98	3.15
TraesCS1B03G0019900	FD1B_gamma4_functional_Intact	Chr1B	6198006	6198056	19.16	7.88	2.43	17.00	5.88	2.89
TraesCS1B03G0019900	FD1B_gamma4_functional_Intact	Chr1B	6198056	6198106	21.41	9.41	2.28	18.55	7.12	2.61
TraesCS1B03G0020100	FD1B_gamma5_functional_Deletion	Chr1B	6258719	6258769	25.25	3.31	7.62	25.25	3.00	8.42
TraesCS1B03G0020100	FD1B_gamma5_functional_Deletion	Chr1B	6258769	6258819	23.39	5.04	4.64	23.39	2.98	7.85
TraesCS1B03G0020100	FD1B_gamma5_functional_Deletion	Chr1B	6258819	6258869	21.53	7.37	2.92	21.20	4.37	4.85
TraesCS1B03G0020100	FD1B_gamma5_functional_Deletion	Chr1B	6258869	6258919	21.22	8.59	2.47	19.12	5.65	3.39
TraesCS1B03G0020100	FD1B_gamma5_functional_Deletion	Chr1B	6258919	6258969	22.53	6.80	3.31	18.96	4.57	4.15
TraesCS1B03G0020100	FD1B_gamma5_functional_Deletion	Chr1B	6258969	6259019	24.57	7.06	3.48	18.71	5.06	3.70
TraesCS1B03G0020100	FD1B_gamma5_functional_Deletion	Chr1B	6259019	6259069	20.20	6.37	3.17	14.33	3.90	3.67
TraesCS1B03G0020100	FD1B_gamma5_functional_Deletion	Chr1B	6259069	6259119	20.76	5.69	3.65	15.16	4.35	3.48
TraesCS1B03G0020100	FD1B_gamma5_functional_Deletion	Chr1B	6259119	6259169	25.41	6.90	3.68	20.69	5.94	3.48
TraesCS1B03G0020100	FD1B_gamma5_functional_Deletion	Chr1B	6259169	6259219	31.43	7.78	4.04	28.88	7.43	3.89
TraesCS1B03G0020100	FD1B_gamma5_functional_Deletion	Chr1B	6259219	6259269	31.78	12.73	2.50	31.35	10.84	2.89

TraesCS1B03G0020100	FD1B_gamma5_functional_Deletion	Chr1B	6259269	6259319	26.16	13.47	1.94	26.16	11.22	2.33
TraesCS1B03G0020100	FD1B_gamma5_functional_Deletion	Chr1B	6259319	6259369	24.04	17.45	1.38	24.04	15.18	1.58
TraesCS1B03G0020100	FD1B_gamma5_functional_Deletion	Chr1B	6259369	6259419	26.71	15.16	1.76	26.71	14.16	1.89
TraesCS1B03G0020100	FD1B_gamma5_functional_Deletion	Chr1B	6259419	6259469	27.24	11.22	2.43	27.24	10.59	2.57
TraesCS1B03G0020100	FD1B_gamma5_functional_Deletion	Chr1B	6259469	6259519	24.67	8.92	2.76	24.67	8.92	2.76
TraesCS1B03G0020100	FD1B_gamma5_functional_Deletion	Chr1B	6259519	6259569	19.63	12.90	1.52	19.63	11.35	1.73
TraesCS1B03G0020100	FD1B_gamma5_functional_Deletion	Chr1B	6259569	6259619	20.69	18.29	1.13	20.69	14.04	1.47
TraesCS1B03G0020100	FD1B_gamma5_functional_Deletion	Chr1B	6259619	6259669	22.92	17.55	1.31	22.92	11.16	2.05
TraesCS1B03G0020100	FD1B_gamma5_functional_Deletion	Chr1B	6259669	6259719	23.35	14.47	1.61	22.65	9.33	2.43
TraesCS1B03G0020100	FD1B_gamma5_functional_Deletion	Chr1B	6259719	6259769	18.76	12.63	1.49	16.76	9.47	1.77
TraesCS1B03G0020100	FD1B_gamma5_functional_Deletion	Chr1B	6259769	6259819	15.00	8.88	1.69	13.00	7.29	1.78
TraesCS1B03G0020500	FD1B_gamma6_functional_Intact	Chr1B	6296327	6296377	25.27	7.92	3.19	25.27	7.06	3.58
TraesCS1B03G0020500	FD1B_gamma6_functional_Intact	Chr1B	6296377	6296427	19.37	7.29	2.66	19.37	7.29	2.66
TraesCS1B03G0020500	FD1B_gamma6_functional_Intact	Chr1B	6296427	6296477	12.39	6.53	1.90	12.39	5.31	2.33
TraesCS1B03G0020500	FD1B_gamma6_functional_Intact	Chr1B	6296477	6296527	15.47	6.98	2.22	12.84	4.33	2.96
TraesCS1B03G0020500	FD1B_gamma6_functional_Intact	Chr1B	6296527	6296577	20.57	6.65	3.09	15.53	3.67	4.24
TraesCS1B03G0020500	FD1B_gamma6_functional_Intact	Chr1B	6296577	6296627	22.73	10.49	2.17	16.76	8.10	2.07
TraesCS1B03G0020500	FD1B_gamma6_functional_Intact	Chr1B	6296627	6296677	15.92	11.37	1.40	12.12	10.16	1.19
TraesCS1B03G0020500	FD1B_gamma6_functional_Intact	Chr1B	6296677	6296727	12.94	11.22	1.15	10.10	10.22	0.99
TraesCS1B03G0020500	FD1B_gamma6_functional_Intact	Chr1B	6296727	6296777	16.16	11.10	1.46	13.10	11.04	1.19
TraesCS1B03G0020500	FD1B_gamma6_functional_Intact	Chr1B	6296777	6296827	19.71	12.69	1.55	16.37	12.69	1.29
TraesCS1B03G0020500	FD1B_gamma6_functional_Intact	Chr1B	6296827	6296877	21.31	14.92	1.43	18.57	14.92	1.24
TraesCS1B03G0020500	FD1B_gamma6_functional_Intact	Chr1B	6296877	6296927	20.20	13.65	1.48	19.14	13.65	1.40
TraesCS1B03G0020500	FD1B_gamma6_functional_Intact	Chr1B	6296927	6296977	24.80	15.80	1.57	23.14	15.41	1.50
TraesCS1B03G0020500	FD1B_gamma6_functional_Intact	Chr1B	6296977	6297027	29.02	17.00	1.71	27.78	15.00	1.85
TraesCS1B03G0020500	FD1B_gamma6_functional_Intact	Chr1B	6297027	6297077	32.53	15.80	2.06	31.53	13.80	2.28
TraesCS1B03G0020500	FD1B_gamma6_functional_Intact	Chr1B	6297077	6297127	33.75	9.90	3.41	33.43	8.69	3.85
TraesCS1B03G0020500	FD1B_gamma6_functional_Intact	Chr1B	6297127	6297177	30.31	8.27	3.66	29.65	8.27	3.58
TraesCS1B03G0020500	FD1B_gamma6_functional_Intact	Chr1B	6297177	6297227	29.63	13.25	2.24	29.00	13.25	2.19
TraesCS1B03G0020500	FD1B_gamma6_functional_Intact	Chr1B	6297227	6297277	30.37	14.61	2.08	30.37	14.61	2.08
TraesCS1B03G0020500	FD1B_gamma6_functional_Intact	Chr1B	6297277	6297327	37.12	13.10	2.83	36.63	13.04	2.81
TraesCS1B03G0020600	FD1B_gamma7_functional_Intact	Chr1B	6301249	6301299	11.08	8.92	1.24	9.24	8.31	1.11
TraesCS1B03G0020600	FD1B_gamma7_functional_Intact	Chr1B	6301299	6301349	14.16	9.31	1.52	12.29	7.22	1.70
TraesCS1B03G0020600	FD1B_gamma7_functional_Intact	Chr1B	6301349	6301399	16.16	13.53	1.19	14.16	9.37	1.51
TraesCS1B03G0020600	FD1B_gamma7_functional_Intact	Chr1B	6301399	6301449	17.47	12.90	1.35	15.57	8.12	1.92
TraesCS1B03G0020600	FD1B_gamma7_functional_Intact	Chr1B	6301449	6301499	17.08	8.08	2.11	15.61	6.06	2.58
TraesCS1B03G0020600	FD1B_gamma7_functional_Intact	Chr1B	6301499	6301549	21.06	10.14	2.08	19.41	8.02	2.42
TraesCS1B03G0020600	FD1B_gamma7_functional_Intact	Chr1B	6301549	6301599	19.61	11.61	1.69	18.47	10.27	1.80
TraesCS1B03G0020600	FD1B_gamma7_functional_Intact	Chr1B	6301599	6301649	19.76	10.94	1.81	19.71	10.94	1.80
TraesCS1B03G0020600	FD1B_gamma7_functional_Intact	Chr1B	6301649	6301699	21.67	5.63	3.85	21.67	5.63	3.85
TraesCS1B03G0020600	FD1B_gamma7_functional_Intact	Chr1B	6301699	6301749	23.20	7.04	3.30	23.04	7.04	3.27
TraesCS1B03G0020600	FD1B_gamma7_functional_Intact	Chr1B	6301749	6301799	21.14	9.33	2.26	20.14	8.90	2.26
TraesCS1B03G0020600	FD1B_gamma7_functional_Intact	Chr1B	6301799	6301849	15.08	11.76	1.28	14.08	10.76	1.31
TraesCS1B03G0020600	FD1B_gamma7_functional_Intact	Chr1B	6301849	6301899	20.55	13.55	1.52	19.43	12.55	1.55
TraesCS1B03G0020600	FD1B_gamma7_functional_Intact	Chr1B	6301899	6301949	25.18	17.24	1.46	24.18	16.76	1.44
TraesCS1B03G0020600	FD1B_gamma7_functional_Intact	Chr1B	6301949	6301999	27.22	15.06	1.81	25.27	13.76	1.84
TraesCS1B03G0020600	FD1B_gamma7_functional_Intact	Chr1B	6301999	6302049	24.96	10.59	2.36	23.88	8.59	2.78
TraesCS1B03G0020600	FD1B_gamma7_functional_Intact	Chr1B	6302049	6302099	23.84	8.98	2.66	23.84	6.98	3.42
TraesCS1B03G0020600	FD1B_gamma7_functional_Intact	Chr1B	6302099	6302149	26.16	10.59	2.47	26.16	9.47	2.76
NCBI_LOC123104557	FD1B_gamma8_functional_Deletion	Chr1B	23863181	23863231	20.24	12.71	1.59	20.00	12.71	1.57
NCBI_LOC123104557	FD1B_gamma8_functional_Deletion	Chr1B	23863231	23863281	21.24	16.39	1.30	20.24	16.04	1.26

NCBI_LOC123104557	FD1B_gamma8_functional_Deletion	Chr1B	23863281	23863331	20.27	15.12	1.34	19.27	14.12	1.37
NCBI_LOC123104557	FD1B_gamma8_functional_Deletion	Chr1B	23863331	23863381	15.55	9.47	1.64	12.45	7.94	1.57
NCBI_LOC123104557	FD1B_gamma8_functional_Deletion	Chr1B	23863381	23863431	12.06	6.76	1.78	6.14	4.35	1.41
NCBI_LOC123104557	FD1B_gamma8_functional_Deletion	Chr1B	23863431	23863481	11.33	6.18	1.83	5.98	5.18	1.16
NCBI_LOC123104557	FD1B_gamma8_functional_Deletion	Chr1B	23863481	23863531	10.06	6.78	1.48	8.25	4.53	1.82
NCBI_LOC123104557	FD1B_gamma8_functional_Deletion	Chr1B	23863531	23863581	13.84	6.22	2.23	12.96	4.22	3.07
NCBI_LOC123104557	FD1B_gamma8_functional_Deletion	Chr1B	23863581	23863631	17.25	4.75	3.64	15.43	2.75	5.62
NCBI_LOC123104557	FD1B_gamma8_functional_Deletion	Chr1B	23863631	23863681	20.75	3.18	6.53	14.80	2.88	5.14
NCBI_LOC123104557	FD1B_gamma8_functional_Deletion	Chr1B	23863681	23863731	21.69	3.82	5.67	15.08	1.63	9.27
NCBI_LOC123104557	FD1B_gamma8_functional_Deletion	Chr1B	23863731	23863781	18.39	5.00	3.68	12.80	1.00	12.80
NCBI_LOC123104557	FD1B_gamma8_functional_Deletion	Chr1B	23863781	23863831	14.82	5.04	2.94	12.61	1.00	12.61
NCBI_LOC123104557	FD1B_gamma8_functional_Deletion	Chr1B	23863831	23863881	13.92	2.18	6.40	12.33	0.47	26.21
NCBI_LOC123104557	FD1B_gamma8_functional_Deletion	Chr1B	23863881	23863931	14.84	2.08	7.14	13.75	1.16	11.88
NCBI_LOC123104557	FD1B_gamma8_functional_Deletion	Chr1B	23863931	23863981	18.61	4.78	3.89	17.51	2.47	7.09
NCBI_LOC123104557	FD1B_gamma8_functional_Deletion	Chr1B	23863981	23864031	17.84	5.75	3.11	17.49	2.75	6.37
NCBI_LOC123104557	FD1B_gamma8_functional_Deletion	Chr1B	23864031	23864081	19.98	4.04	4.95	19.98	2.69	7.44
NCBI_LOC123104557	FD1B_gamma8_functional_Deletion	Chr1B	23864081	23864131	20.10	1.00	20.10	19.24	1.00	19.24
NCBI_LOC123104557	FD1B_gamma8_functional_Deletion	Chr1B	23864131	23864181	18.02	0.78	22.97	17.73	0.78	22.60
NCBI_LOC123104557	FD1B_gamma8_functional_Deletion	Chr1B	23864181	23864231	17.16	1.90	9.02	16.63	1.90	8.74
NCBI_LOC123104557	FD1B_gamma8_functional_Deletion	Chr1B	23864231	23864281	16.27	5.14	3.17	15.71	5.14	3.06
NCBI_LOC123104557	FD1B_gamma8_functional_Deletion	Chr1B	23864281	23864331	19.96	7.31	2.73	19.96	7.31	2.73
TraesCS1B03G0021300	FD1B_omega1_pseudogene_Deletion	Chr1B	6428880	6428930	18.14	6.37	2.85	18.06	6.37	2.83
TraesCS1B03G0021300	FD1B_omega1_pseudogene_Deletion	Chr1B	6428930	6428980	15.98	9.04	1.77	14.98	9.04	1.66
TraesCS1B03G0021300	FD1B_omega1_pseudogene_Deletion	Chr1B	6428980	6429030	13.53	9.76	1.39	12.53	9.10	1.38
TraesCS1B03G0021300	FD1B_omega1_pseudogene_Deletion	Chr1B	6429030	6429080	8.94	9.80	0.91	8.06	8.82	0.91
TraesCS1B03G0021300	FD1B_omega1_pseudogene_Deletion	Chr1B	6429080	6429130	4.43	6.45	0.69	4.43	4.43	1.00
TraesCS1B03G0021300	FD1B_omega1_pseudogene_Deletion	Chr1B	6429130	6429180	3.94	6.14	0.64	2.35	2.59	0.91
TraesCS1B03G0021300	FD1B_omega1_pseudogene_Deletion	Chr1B	6429180	6429230	7.47	7.12	1.05	3.65	1.86	1.96
TraesCS1B03G0021300	FD1B_omega1_pseudogene_Deletion	Chr1B	6429230	6429280	6.53	5.86	1.11	2.27	3.00	0.76
TraesCS1B03G0021300	FD1B_omega1_pseudogene_Deletion	Chr1B	6429280	6429330	1.88	4.22	0.45	1.12	2.57	0.44
TraesCS1B03G0021300	FD1B_omega1_pseudogene_Deletion	Chr1B	6429330	6429380	3.94	4.86	0.81	3.94	2.39	1.65
TraesCS1B03G0021300	FD1B_omega1_pseudogene_Deletion	Chr1B	6429380	6429430	7.31	8.04	0.91	6.88	3.20	2.15
TraesCS1B03G0021300	FD1B_omega1_pseudogene_Deletion	Chr1B	6429430	6429480	10.92	7.90	1.38	9.37	4.10	2.29
TraesCS1B03G0021300	FD1B_omega1_pseudogene_Deletion	Chr1B	6429480	6429530	9.88	7.94	1.24	8.25	4.35	1.90
TraesCS1B03G0021300	FD1B_omega1_pseudogene_Deletion	Chr1B	6429530	6429580	13.10	8.04	1.63	11.22	2.86	3.92
TraesCS1B03G0021300	FD1B_omega1_pseudogene_Deletion	Chr1B	6429580	6429630	12.18	3.04	4.01	9.57	0.29	32.53
TraesCS1B03G0021300	FD1B_omega1_pseudogene_Deletion	Chr1B	6429630	6429680	16.78	2.06	8.15	11.57	0.76	15.13
TraesCS1B03G0021300	FD1B_omega1_pseudogene_Deletion	Chr1B	6429680	6429730	15.96	3.63	4.40	10.84	1.63	6.66
TraesCS1B03G0021300	FD1B_omega1_pseudogene_Deletion	Chr1B	6429730	6429780	12.69	4.61	2.75	9.67	2.37	4.07
TraesCS1B03G0021300	FD1B_omega1_pseudogene_Deletion	Chr1B	6429780	6429830	13.14	3.92	3.35	8.14	2.12	3.84
TraesCS1B03G0021300	FD1B_omega1_pseudogene_Deletion	Chr1B	6429830	6429880	11.39	5.06	2.25	8.02	2.20	3.65
TraesCS1B03G0021300	FD1B_omega1_pseudogene_Deletion	Chr1B	6429880	6429930	8.88	4.61	1.93	6.69	1.84	3.63
TraesCS1B03G0021300	FD1B_omega1_pseudogene_Deletion	Chr1B	6429930	6429980	7.61	5.94	1.28	5.39	2.65	2.04
TraesCS1B03G0021300	FD1B_omega1_pseudogene_Deletion	Chr1B	6429980	6430030	5.88	5.84	1.01	5.02	3.16	1.59
TraesCS1B03G0021300	FD1B_omega1_pseudogene_Deletion	Chr1B	6430030	6430080	11.43	5.86	1.95	10.73	3.39	3.16
TraesCS1B03G0021300	FD1B_omega1_pseudogene_Deletion	Chr1B	6430080	6430130	17.57	8.57	2.05	17.00	6.31	2.69
TraesCS1B03G0021300	FD1B_omega1_pseudogene_Deletion	Chr1B	6430130	6430180	19.27	14.49	1.33	19.27	11.35	1.70
TraesCS1B03G0021300	FD1B_omega1_pseudogene_Deletion	Chr1B	6430180	6430230	21.24	15.90	1.34	20.75	11.90	1.74
TraesCS1B03G0021300	FD1B_omega1_pseudogene_Deletion	Chr1B	6430230	6430280	18.76	13.94	1.35	17.76	10.31	1.72
-	FD1B_omega5like_partial_Deletion	Chr1B	6470900	6470950	14.63	14.67	1.00	14.63	13.67	1.07
-	FD1B_omega5like_partial_Deletion	Chr1B	6470950	6471000	16.31	9.63	1.69	15.61	8.22	1.90

-	FD1B_omega5like_partial_Deletion	Chr1B	6471000	6471050	13.86	5.41	2.56	12.78	4.41	2.90
-	FD1B_omega5like_partial_Deletion	Chr1B	6471050	6471100	12.29	2.24	5.50	10.35	1.08	9.60
-	FD1B_omega5like_partial_Deletion	Chr1B	6471100	6471150	9.63	1.35	7.12	6.75	0.00	6.75
-	FD1B_omega5like_partial_Deletion	Chr1B	6471150	6471200	8.94	0.98	9.12	4.49	0.16	28.62
-	FD1B_omega5like_partial_Deletion	Chr1B	6471200	6471250	4.75	1.20	3.97	1.90	1.00	1.90
-	FD1B_omega5like_partial_Deletion	Chr1B	6471250	6471300	3.39	1.98	1.71	1.90	1.00	1.90
-	FD1B_omega5like_partial_Deletion	Chr1B	6471300	6471350	1.47	2.76	0.53	1.25	1.76	0.71
-	FD1B_omega5like_partial_Deletion	Chr1B	6471350	6471400	6.27	5.55	1.13	3.71	4.63	0.80
-	FD1B_omega5like_partial_Deletion	Chr1B	6471400	6471450	11.35	9.43	1.20	6.84	6.96	0.98
-	FD1B_omega5like_partial_Deletion	Chr1B	6471450	6471500	15.04	11.69	1.29	9.51	6.25	1.52
-	FD1B_omega5like_partial_Deletion	Chr1B	6471500	6471550	12.51	8.57	1.46	9.51	4.43	2.15
-	FD1B_omega5like_partial_Deletion	Chr1B	6471550	6471600	10.57	3.37	3.13	8.90	1.71	5.22
-	FD1B_omega5like_partial_Deletion	Chr1B	6471600	6471650	8.18	2.29	3.56	8.18	0.96	8.51
-	FD1B_omega5like_partial_Deletion	Chr1B	6471650	6471700	8.80	2.00	4.40	8.80	0.00	8.80
-	FD1B_omega5like_partial_Deletion	Chr1B	6471700	6471750	5.51	2.47	2.23	5.51	0.00	5.51
-	FD1B_omega5like_partial_Deletion	Chr1B	6471750	6471800	4.55	1.69	2.70	3.65	0.00	3.65
-	FD1B_omega5like_partial_Deletion	Chr1B	6471800	6471850	10.29	2.02	5.10	5.20	0.00	5.20
-	FD1B_omega5like_partial_Deletion	Chr1B	6471850	6471900	9.24	2.18	4.24	7.20	0.00	7.20
-	FD1B_omega5like_partial_Deletion	Chr1B	6471900	6471950	9.22	1.06	8.70	7.80	0.00	7.80
-	FD1B_omega5like_partial_Deletion	Chr1B	6471950	6472000	10.65	3.25	3.27	9.47	0.00	9.47
-	FD1B_omega5like_partial_Deletion	Chr1B	6472000	6472050	11.04	3.43	3.22	10.04	1.02	9.85
-	FD1B_omega5like_partial_Deletion	Chr1B	6472050	6472100	12.63	5.98	2.11	10.63	3.29	3.23
-	FD1B_omega5like_partial_Deletion	Chr1B	6472100	6472150	15.98	10.51	1.52	13.98	8.51	1.64
-	FD1B_omega5like_partial_Deletion	Chr1B	6472150	6472200	16.02	8.31	1.93	14.86	7.08	2.10
TraesCSU03G0200600	FD1B_omega2_partial_Deletion	Chr1B	6498351	6498401	25.27	18.00	1.40	21.63	12.20	1.77
TraesCSU03G0200600	FD1B_omega2_partial_Deletion	Chr1B	6498401	6498451	25.37	13.90	1.83	23.27	8.67	2.69
TraesCSU03G0200600	FD1B_omega2_partial_Deletion	Chr1B	6498451	6498501	24.22	9.84	2.46	20.00	5.65	3.54
TraesCSU03G0200600	FD1B_omega2_partial_Deletion	Chr1B	6498501	6498551	27.88	8.24	3.39	19.47	4.41	4.41
TraesCSU03G0200600	FD1B_omega2_partial_Deletion	Chr1B	6498551	6498601	25.78	7.31	3.53	18.88	1.39	13.56
TraesCSU03G0200600	FD1B_omega2_partial_Deletion	Chr1B	6498601	6498651	22.53	12.71	1.77	16.84	2.31	7.28
TraesCSU03G0200600	FD1B_omega2_partial_Deletion	Chr1B	6498651	6498701	14.75	10.43	1.41	11.63	2.06	5.65
TraesCSU03G0200600	FD1B_omega2_partial_Deletion	Chr1B	6498701	6498751	10.80	4.00	2.70	9.63	1.35	7.12
TraesCSU03G0200600	FD1B_omega2_partial_Deletion	Chr1B	6498751	6498801	12.61	2.94	4.29	8.18	0.45	18.13
TraesCSU03G0200600	FD1B_omega2_partial_Deletion	Chr1B	6498801	6498851	18.43	5.16	3.57	9.92	1.00	9.92
TraesCSU03G0200600	FD1B_omega2_partial_Deletion	Chr1B	6498851	6498901	16.33	7.86	2.08	9.04	0.69	13.17
TraesCSU03G0200600	FD1B_omega2_partial_Deletion	Chr1B	6498901	6498951	10.25	5.65	1.82	7.04	1.47	4.79
TraesCSU03G0200600	FD1B_omega2_partial_Deletion	Chr1B	6498951	6499001	6.10	3.27	1.86	3.39	2.00	1.70
TraesCSU03G0200600	FD1B_omega2_partial_Deletion	Chr1B	6499001	6499051	7.53	2.43	3.10	3.02	0.94	3.21
TraesCSU03G0200600	FD1B_omega2_partial_Deletion	Chr1B	6499051	6499101	10.71	5.20	2.06	3.41	0.00	3.41
TraesCSU03G0200600	FD1B_omega2_partial_Deletion	Chr1B	6499101	6499151	14.51	7.00	2.07	4.67	0.00	4.67
TraesCSU03G0200600	FD1B_omega2_partial_Deletion	Chr1B	6499151	6499201	15.04	6.35	2.37	5.45	0.00	5.45
TraesCSU03G0200600	FD1B_omega2_partial_Deletion	Chr1B	6499201	6499251	16.63	5.53	3.01	5.18	0.51	10.15
TraesCSU03G0200600	FD1B_omega2_partial_Deletion	Chr1B	6499251	6499301	10.75	1.88	5.71	5.92	1.04	5.70
TraesCSU03G0200600	FD1B_omega2_partial_Deletion	Chr1B	6499301	6499351	8.29	2.84	2.92	6.14	2.00	3.07
TraesCSU03G0200600	FD1B_omega2_partial_Deletion	Chr1B	6499351	6499401	12.76	4.49	2.84	9.08	2.25	4.03
TraesCSU03G0200600	FD1B_omega2_partial_Deletion	Chr1B	6499401	6499451	15.90	5.20	3.06	13.67	2.86	4.77
TraesCSU03G0200600	FD1B_omega2_partial_Deletion	Chr1B	6499451	6499501	18.16	5.31	3.42	14.35	2.00	7.18
TraesCSU03G0200600	FD1B_omega2_partial_Deletion	Chr1B	6499501	6499551	16.73	4.69	3.57	12.45	1.73	7.22
TraesCSU03G0200600	FD1B_omega2_partial_Deletion	Chr1B	6499551	6499601	24.35	11.27	2.16	13.82	4.57	3.03
TraesCSU03G0200600	FD1B_omega2_partial_Deletion	Chr1B	6499601	6499651	27.61	16.57	1.67	19.39	8.25	2.35
TraesCSU03G0200600	FD1B_omega2_partial_Deletion	Chr1B	6499651	6499701	34.16	19.73	1.73	21.20	8.08	2.62

TraesCS1B03G0021500	FD1B_omega3_functional_Deletion	Chr1B	6507784	6507834	21.04	7.69	2.74	20.10	4.20	4.79
TraesCS1B03G0021500	FD1B_omega3_functional_Deletion	Chr1B	6507834	6507884	18.76	7.63	2.46	16.31	5.14	3.18
TraesCS1B03G0021500	FD1B_omega3_functional_Deletion	Chr1B	6507884	6507934	14.43	6.33	2.28	11.43	3.49	3.28
TraesCS1B03G0021500	FD1B_omega3_functional_Deletion	Chr1B	6507934	6507984	10.43	6.24	1.67	7.18	2.88	2.49
TraesCS1B03G0021500	FD1B_omega3_functional_Deletion	Chr1B	6507984	6508034	10.49	6.10	1.72	5.86	1.96	2.99
TraesCS1B03G0021500	FD1B_omega3_functional_Deletion	Chr1B	6508034	6508084	19.53	9.14	2.14	8.69	2.43	3.57
TraesCS1B03G0021500	FD1B_omega3_functional_Deletion	Chr1B	6508084	6508134	19.84	11.12	1.78	7.90	3.84	2.06
TraesCS1B03G0021500	FD1B_omega3_functional_Deletion	Chr1B	6508134	6508184	17.14	7.92	2.16	5.49	3.25	1.69
TraesCS1B03G0021500	FD1B_omega3_functional_Deletion	Chr1B	6508184	6508234	12.98	5.43	2.39	4.61	4.35	1.06
TraesCS1B03G0021500	FD1B_omega3_functional_Deletion	Chr1B	6508234	6508284	13.69	8.25	1.66	4.90	4.00	1.23
TraesCS1B03G0021500	FD1B_omega3_functional_Deletion	Chr1B	6508284	6508334	11.82	8.12	1.46	4.65	3.49	1.33
TraesCS1B03G0021500	FD1B_omega3_functional_Deletion	Chr1B	6508334	6508384	12.67	6.51	1.95	6.12	3.18	1.93
TraesCS1B03G0021500	FD1B_omega3_functional_Deletion	Chr1B	6508384	6508434	10.06	6.04	1.67	6.18	3.86	1.60
TraesCS1B03G0021500	FD1B_omega3_functional_Deletion	Chr1B	6508434	6508484	7.24	7.00	1.03	2.94	3.25	0.90
TraesCS1B03G0021500	FD1B_omega3_functional_Deletion	Chr1B	6508484	6508534	6.20	3.55	1.75	1.57	0.37	4.21
TraesCS1B03G0021500	FD1B_omega3_functional_Deletion	Chr1B	6508534	6508584	3.45	0.20	17.60	1.55	0.00	1.55
TraesCS1B03G0021500	FD1B_omega3_functional_Deletion	Chr1B	6508584	6508634	2.67	0.20	13.60	2.35	0.00	2.35
TraesCS1B03G0021500	FD1B_omega3_functional_Deletion	Chr1B	6508634	6508684	6.10	1.22	5.02	4.55	0.00	4.55
TraesCS1B03G0021500	FD1B_omega3_functional_Deletion	Chr1B	6508684	6508734	10.02	2.10	4.78	5.18	1.10	4.71
TraesCS1B03G0021500	FD1B_omega3_functional_Deletion	Chr1B	6508734	6508784	12.33	5.18	2.38	5.53	2.45	2.26
TraesCS1B03G0021500	FD1B_omega3_functional_Deletion	Chr1B	6508784	6508834	11.37	7.63	1.49	3.20	3.22	0.99
TraesCS1B03G0021500	FD1B_omega3_functional_Deletion	Chr1B	6508834	6508884	14.96	8.61	1.74	4.45	4.16	1.07
TraesCS1B03G0021500	FD1B_omega3_functional_Deletion	Chr1B	6508884	6508934	16.92	8.67	1.95	7.73	4.84	1.60
TraesCS1B03G0021500	FD1B_omega3_functional_Deletion	Chr1B	6508934	6508984	14.94	10.39	1.44	9.33	7.12	1.31
TraesCS1B03G0021500	FD1B_omega3_functional_Deletion	Chr1B	6508984	6509034	20.37	13.29	1.53	14.49	7.71	1.88
TraesCS1B03G0021500	FD1B_omega3_functional_Deletion	Chr1B	6509034	6509084	19.94	16.43	1.21	15.86	10.37	1.53
TraesCS1B03G0021500	FD1B_omega3_functional_Deletion	Chr1B	6509084	6509134	21.39	19.18	1.12	18.78	13.16	1.43
TraesCSU03G0331200	FD1B_omega4_pseudogene_Deletion	Chr1B	6520158	6520208	30.78	20.82	1.48	26.49	16.18	1.64
TraesCSU03G0331200	FD1B_omega4_pseudogene_Deletion	Chr1B	6520208	6520258	24.73	14.63	1.69	20.73	10.22	2.03
TraesCSU03G0331200	FD1B_omega4_pseudogene_Deletion	Chr1B	6520258	6520308	25.29	8.43	3.00	21.12	3.78	5.58
TraesCSU03G0331200	FD1B_omega4_pseudogene_Deletion	Chr1B	6520308	6520358	21.69	7.20	3.01	16.94	0.41	41.14
TraesCSU03G0331200	FD1B_omega4_pseudogene_Deletion	Chr1B	6520358	6520408	18.31	6.06	3.02	13.82	0.00	13.82
TraesCSU03G0331200	FD1B_omega4_pseudogene_Deletion	Chr1B	6520408	6520458	19.04	5.47	3.48	13.00	0.00	13.00
TraesCSU03G0331200	FD1B_omega4_pseudogene_Deletion	Chr1B	6520458	6520508	15.84	2.92	5.42	11.96	0.00	11.96
TraesCSU03G0331200	FD1B_omega4_pseudogene_Deletion	Chr1B	6520508	6520558	11.75	2.47	4.75	7.39	0.00	7.39
TraesCSU03G0331200	FD1B_omega4_pseudogene_Deletion	Chr1B	6520558	6520608	8.37	1.49	5.62	4.04	0.00	4.04
TraesCSU03G0331200	FD1B_omega4_pseudogene_Deletion	Chr1B	6520608	6520658	10.86	0.14	79.14	3.78	0.00	3.78
TraesCSU03G0331200	FD1B_omega4_pseudogene_Deletion	Chr1B	6520658	6520708	14.84	2.37	6.26	6.65	0.29	22.60
TraesCSU03G0331200	FD1B_omega4_pseudogene_Deletion	Chr1B	6520708	6520758	13.02	3.10	4.20	7.76	1.00	7.76
TraesCSU03G0331200	FD1B_omega4_pseudogene_Deletion	Chr1B	6520758	6520808	6.65	3.88	1.71	4.65	1.80	2.58
TraesCSU03G0331200	FD1B_omega4_pseudogene_Deletion	Chr1B	6520808	6520858	5.98	2.45	2.44	3.31	1.61	2.06
TraesCSU03G0331200	FD1B_omega4_pseudogene_Deletion	Chr1B	6520858	6520908	9.10	1.80	5.04	2.20	0.80	2.73
TraesCSU03G0331200	FD1B_omega4_pseudogene_Deletion	Chr1B	6520908	6520958	9.06	1.76	5.13	1.71	0.00	1.71
TraesCSU03G0331200	FD1B_omega4_pseudogene_Deletion	Chr1B	6520958	6521008	6.16	2.94	2.09	2.45	0.04	62.50
TraesCSU03G0331200	FD1B_omega4_pseudogene_Deletion	Chr1B	6521008	6521058	8.22	6.43	1.28	4.41	2.51	1.76
TraesCSU03G0331200	FD1B_omega4_pseudogene_Deletion	Chr1B	6521058	6521108	12.10	7.20	1.68	9.10	3.57	2.55
TraesCSU03G0331200	FD1B_omega4_pseudogene_Deletion	Chr1B	6521108	6521158	14.47	6.04	2.40	11.57	4.71	2.46
TraesCSU03G0331200	FD1B_omega4_pseudogene_Deletion	Chr1B	6521158	6521208	13.65	3.76	3.63	12.33	3.53	3.49
TraesCSU03G0331200	FD1B_omega4_pseudogene_Deletion	Chr1B	6521208	6521258	15.78	4.27	3.69	13.41	3.37	3.98
TraesCSU03G0331200	FD1B_omega4_pseudogene_Deletion	Chr1B	6521258	6521308	13.33	7.92	1.68	11.04	6.47	1.71
TraesCSU03G0331200	FD1B_omega4_pseudogene_Deletion	Chr1B	6521308	6521358	17.27	11.90	1.45	16.27	9.45	1.72

TraesCSU03G0331200	FD1B_omega4_pseudogene_Deletion	Chr1B	6521358	6521408	20.61	18.55	1.11	20.08	15.37	1.31
TraesCSU03G0331200	FD1B_omega4_pseudogene_Deletion	Chr1B	6521408	6521458	21.08	18.22	1.16	19.10	14.39	1.33
TraesCSU03G0331200	FD1B_omega4_pseudogene_Deletion	Chr1B	6521458	6521508	17.43	17.31	1.01	13.63	13.00	1.05
TraesCSU03G0331200	FD1B_omega4_pseudogene_Deletion	Chr1B	6521508	6521558	13.00	16.45	0.79	8.45	10.57	0.80
TraesCS1B03G0021900	FD1B_omega5_functional_Deletion	Chr1B	6531518	6531568	29.02	20.18	1.44	18.41	9.96	1.85
TraesCS1B03G0021900	FD1B_omega5_functional_Deletion	Chr1B	6531568	6531618	34.49	18.06	1.91	20.02	10.49	1.91
TraesCS1B03G0021900	FD1B_omega5_functional_Deletion	Chr1B	6531618	6531668	28.16	14.76	1.91	16.35	8.47	1.93
TraesCS1B03G0021900	FD1B_omega5_functional_Deletion	Chr1B	6531668	6531718	18.78	7.14	2.63	10.02	2.90	3.45
TraesCS1B03G0021900	FD1B_omega5_functional_Deletion	Chr1B	6531718	6531768	18.47	7.31	2.53	8.63	2.71	3.19
TraesCS1B03G0021900	FD1B_omega5_functional_Deletion	Chr1B	6531768	6531818	24.10	14.84	1.62	8.69	8.29	1.05
TraesCS1B03G0021900	FD1B_omega5_functional_Deletion	Chr1B	6531818	6531868	24.98	19.06	1.31	9.06	9.82	0.92
TraesCS1B03G0021900	FD1B_omega5_functional_Deletion	Chr1B	6531868	6531918	23.02	16.33	1.41	6.84	6.65	1.03
TraesCS1B03G0021900	FD1B_omega5_functional_Deletion	Chr1B	6531918	6531968	20.57	11.88	1.73	7.61	4.45	1.71
TraesCS1B03G0021900	FD1B_omega5_functional_Deletion	Chr1B	6531968	6532018	26.51	12.10	2.19	12.65	6.37	1.98
TraesCS1B03G0021900	FD1B_omega5_functional_Deletion	Chr1B	6532018	6532068	28.16	14.35	1.96	12.94	5.67	2.28
TraesCS1B03G0021900	FD1B_omega5_functional_Deletion	Chr1B	6532068	6532118	28.65	17.82	1.61	14.80	5.67	2.61
TraesCS1B03G0021900	FD1B_omega5_functional_Deletion	Chr1B	6532118	6532168	26.61	19.14	1.39	16.14	5.00	3.23
TraesCS1B03G0021900	FD1B_omega5_functional_Deletion	Chr1B	6532168	6532218	19.33	14.06	1.38	12.45	2.82	4.41
TraesCS1B03G0021900	FD1B_omega5_functional_Deletion	Chr1B	6532218	6532268	17.63	6.02	2.93	7.27	0.16	46.37
TraesCS1B03G0021900	FD1B_omega5_functional_Deletion	Chr1B	6532268	6532318	9.71	3.00	3.24	2.53	0.00	2.53
TraesCS1B03G0021900	FD1B_omega5_functional_Deletion	Chr1B	6532318	6532368	10.84	2.43	4.46	4.80	0.00	4.80
TraesCS1B03G0021900	FD1B_omega5_functional_Deletion	Chr1B	6532368	6532418	20.39	1.47	13.87	9.14	0.16	58.25
TraesCS1B03G0021900	FD1B_omega5_functional_Deletion	Chr1B	6532418	6532468	20.78	3.04	6.84	12.10	1.00	12.10
TraesCS1B03G0021900	FD1B_omega5_functional_Deletion	Chr1B	6532468	6532518	16.94	3.41	4.97	13.20	2.37	5.56
TraesCS1B03G0021900	FD1B_omega5_functional_Deletion	Chr1B	6532518	6532568	13.94	2.88	4.84	8.98	2.00	4.49
TraesCS1B03G0021900	FD1B_omega5_functional_Deletion	Chr1B	6532568	6532618	17.94	4.78	3.75	7.08	2.00	3.54
TraesCS1B03G0021900	FD1B_omega5_functional_Deletion	Chr1B	6532618	6532668	21.84	3.35	6.51	8.75	0.20	44.60
TraesCS1B03G0021900	FD1B_omega5_functional_Deletion	Chr1B	6532668	6532718	14.78	5.27	2.80	6.06	1.35	4.48
TraesCS1B03G0021900	FD1B_omega5_functional_Deletion	Chr1B	6532718	6532768	20.57	6.04	3.41	11.02	2.22	4.97
TraesCS1B03G0021900	FD1B_omega5_functional_Deletion	Chr1B	6532768	6532818	26.53	8.88	2.99	14.33	4.59	3.12
TraesCS1B03G0021900	FD1B_omega5_functional_Deletion	Chr1B	6532818	6532868	29.00	8.06	3.60	14.43	3.45	4.18
TraesCSU03G0258100	FD1B_omega6_partial_Deletion	Chr1B	6554623	6554673	33.33	11.25	2.96	30.73	11.25	2.73
TraesCSU03G0258100	FD1B_omega6_partial_Deletion	Chr1B	6554673	6554723	30.75	8.22	3.74	27.12	7.88	3.44
TraesCSU03G0258100	FD1B_omega6_partial_Deletion	Chr1B	6554723	6554773	34.53	5.25	6.57	24.04	3.25	7.39
TraesCSU03G0258100	FD1B_omega6_partial_Deletion	Chr1B	6554773	6554823	30.39	5.12	5.94	17.63	2.78	6.33
TraesCSU03G0258100	FD1B_omega6_partial_Deletion	Chr1B	6554823	6554873	29.25	5.51	5.31	12.71	3.41	3.72
TraesCSU03G0258100	FD1B_omega6_partial_Deletion	Chr1B	6554873	6554923	24.92	4.86	5.13	9.12	3.00	3.04
TraesCSU03G0258100	FD1B_omega6_partial_Deletion	Chr1B	6554923	6554973	18.04	5.37	3.36	5.76	1.63	3.54
TraesCSU03G0258100	FD1B_omega6_partial_Deletion	Chr1B	6554973	6555023	12.96	2.35	5.51	6.71	0.00	6.71
TraesCSU03G0258100	FD1B_omega6_partial_Deletion	Chr1B	6555023	6555073	17.39	1.27	13.65	10.76	0.41	26.14
TraesCSU03G0258100	FD1B_omega6_partial_Deletion	Chr1B	6555073	6555123	24.65	1.67	14.79	14.20	1.00	14.20
TraesCSU03G0258100	FD1B_omega6_partial_Deletion	Chr1B	6555123	6555173	24.25	4.04	6.00	12.47	1.06	11.78
TraesCSU03G0258100	FD1B_omega6_partial_Deletion	Chr1B	6555173	6555223	16.63	9.94	1.67	7.73	3.31	2.33
TraesCSU03G0258100	FD1B_omega6_partial_Deletion	Chr1B	6555223	6555273	13.71	7.29	1.88	4.51	2.47	1.83
TraesCSU03G0258100	FD1B_omega6_partial_Deletion	Chr1B	6555273	6555323	12.33	3.67	3.36	3.12	0.00	3.12
TraesCSU03G0258100	FD1B_omega6_partial_Deletion	Chr1B	6555323	6555373	6.47	1.25	5.16	4.08	0.00	4.08
TraesCSU03G0258100	FD1B_omega6_partial_Deletion	Chr1B	6555373	6555423	9.80	2.25	4.35	6.06	0.59	10.30
TraesCSU03G0258100	FD1B_omega6_partial_Deletion	Chr1B	6555423	6555473	21.96	6.59	3.33	11.90	2.25	5.28
TraesCSU03G0258100	FD1B_omega6_partial_Deletion	Chr1B	6555473	6555523	24.20	8.24	2.94	14.22	3.35	4.24
TraesCSU03G0258100	FD1B_omega6_partial_Deletion	Chr1B	6555523	6555573	19.86	10.43	1.90	12.45	5.27	2.36
TraesCSU03G0258100	FD1B_omega6_partial_Deletion	Chr1B	6555573	6555623	16.53	9.02	1.83	8.82	6.47	1.36

TraesCSU03G0258100	FD1B_omega6_partial_Deletion	Chr1B	6555623	6555673	17.39	8.78	1.98	8.06	6.02	1.34
TraesCSU03G0258100	FD1B_omega6_partial_Deletion	Chr1B	6555673	6555723	19.90	7.43	2.68	11.35	4.31	2.63
TraesCSU03G0258100	FD1B_omega6_partial_Deletion	Chr1B	6555723	6555773	22.82	6.67	3.42	16.53	4.10	4.03
TraesCSU03G0258100	FD1B_omega6_partial_Deletion	Chr1B	6555773	6555823	42.51	19.18	2.22	31.67	10.45	3.03
TraesCSU03G0258100	FD1B_omega6_partial_Deletion	Chr1B	6555823	6555873	44.92	24.61	1.83	40.88	19.51	2.10
TraesCSU03G0258100	FD1B_omega6_partial_Deletion	Chr1B	6555873	6555923	43.47	18.94	2.30	41.76	17.80	2.35
-	FD1B_omega7_partial_Deletion	Chr1B	6597449	6597499	13.25	9.43	1.41	13.00	7.43	1.75
-	FD1B_omega7_partial_Deletion	Chr1B	6597499	6597549	13.39	8.06	1.66	13.00	5.43	2.39
-	FD1B_omega7_partial_Deletion	Chr1B	6597549	6597599	9.88	7.00	1.41	8.63	5.18	1.67
-	FD1B_omega7_partial_Deletion	Chr1B	6597599	6597649	7.63	6.14	1.24	5.61	5.14	1.09
-	FD1B_omega7_partial_Deletion	Chr1B	6597649	6597699	7.33	4.75	1.55	3.94	4.61	0.86
-	FD1B_omega7_partial_Deletion	Chr1B	6597699	6597749	7.33	3.49	2.10	3.55	3.00	1.18
-	FD1B_omega7_partial_Deletion	Chr1B	6597749	6597799	6.37	3.90	1.63	5.10	3.00	1.70
-	FD1B_omega7_partial_Deletion	Chr1B	6597799	6597849	6.73	2.84	2.37	5.27	1.90	2.77
-	FD1B_omega7_partial_Deletion	Chr1B	6597849	6597899	8.82	4.82	1.83	3.51	1.94	1.81
-	FD1B_omega7_partial_Deletion	Chr1B	6597899	6597949	9.33	4.24	2.20	3.24	2.00	1.62
-	FD1B_omega7_partial_Deletion	Chr1B	6597949	6597999	8.76	2.27	3.85	5.00	1.43	3.49
-	FD1B_omega7_partial_Deletion	Chr1B	6597999	6598049	8.41	1.86	4.52	4.84	1.16	4.19
-	FD1B_omega7_partial_Deletion	Chr1B	6598049	6598099	5.55	4.86	1.14	3.37	2.71	1.25
-	FD1B_omega7_partial_Deletion	Chr1B	6598099	6598149	6.06	7.96	0.76	5.80	5.20	1.12
-	FD1B_omega7_partial_Deletion	Chr1B	6598149	6598199	9.82	7.63	1.29	7.02	4.94	1.42
-	FD1B_omega7_partial_Deletion	Chr1B	6598199	6598249	10.75	3.59	2.99	6.51	3.10	2.10
-	FD1B_omega7_partial_Deletion	Chr1B	6598249	6598299	8.92	3.12	2.86	4.67	1.92	2.43
-	FD1B_omega7_partial_Deletion	Chr1B	6598299	6598349	5.41	1.90	2.85	3.22	1.14	2.83
-	FD1B_omega7_partial_Deletion	Chr1B	6598349	6598399	5.35	1.61	3.33	3.55	1.61	2.21
-	FD1B_omega7_partial_Deletion	Chr1B	6598399	6598449	9.61	5.57	1.73	6.53	3.16	2.07
-	FD1B_omega7_partial_Deletion	Chr1B	6598449	6598499	9.59	8.14	1.18	7.67	4.35	1.76
-	FD1B_omega7_partial_Deletion	Chr1B	6598499	6598549	8.04	9.59	0.84	6.94	7.43	0.93
-	FD1B_omega7_partial_Deletion	Chr1B	6598549	6598599	11.16	12.75	0.88	9.16	11.63	0.79
-	FD1B_omega7_partial_Deletion	Chr1B	6598599	6598649	13.10	12.18	1.08	12.88	12.08	1.07
-	FD1B_omega7_partial_Deletion	Chr1B	6598649	6598699	15.94	9.69	1.65	15.94	9.69	1.65
TraesCS1B03G0022200	FD1B_omega8_partial_Deletion	Chr1B	6607399	6607449	21.92	10.35	2.12	19.92	10.08	1.98
TraesCS1B03G0022200	FD1B_omega8_partial_Deletion	Chr1B	6607449	6607499	16.06	12.92	1.24	15.04	10.76	1.40
TraesCS1B03G0022200	FD1B_omega8_partial_Deletion	Chr1B	6607499	6607549	12.71	11.67	1.09	12.71	8.78	1.45
TraesCS1B03G0022200	FD1B_omega8_partial_Deletion	Chr1B	6607549	6607599	13.27	8.84	1.50	12.86	6.10	2.11
TraesCS1B03G0022200	FD1B_omega8_partial_Deletion	Chr1B	6607599	6607649	14.53	8.20	1.77	13.16	2.75	4.79
TraesCS1B03G0022200	FD1B_omega8_partial_Deletion	Chr1B	6607649	6607699	13.47	9.25	1.46	11.47	2.27	5.04
TraesCS1B03G0022200	FD1B_omega8_partial_Deletion	Chr1B	6607699	6607749	10.37	9.29	1.12	6.73	3.08	2.18
TraesCS1B03G0022200	FD1B_omega8_partial_Deletion	Chr1B	6607749	6607799	9.92	7.78	1.27	5.55	5.80	0.96
TraesCS1B03G0022200	FD1B_omega8_partial_Deletion	Chr1B	6607799	6607849	6.98	6.75	1.03	4.76	4.90	0.97
TraesCS1B03G0022200	FD1B_omega8_partial_Deletion	Chr1B	6607849	6607899	8.35	1.90	4.39	6.20	0.53	11.70
TraesCS1B03G0022200	FD1B_omega8_partial_Deletion	Chr1B	6607899	6607949	13.59	5.35	2.54	8.39	1.12	7.51
TraesCS1B03G0022200	FD1B_omega8_partial_Deletion	Chr1B	6607949	6607999	15.86	7.22	2.20	8.57	2.00	4.28
TraesCS1B03G0022200	FD1B_omega8_partial_Deletion	Chr1B	6607999	6608049	12.57	7.04	1.79	10.24	2.33	4.39
TraesCS1B03G0022200	FD1B_omega8_partial_Deletion	Chr1B	6608049	6608099	13.84	5.25	2.63	11.67	1.78	6.54
TraesCS1B03G0022200	FD1B_omega8_partial_Deletion	Chr1B	6608099	6608149	17.00	5.31	3.20	11.57	1.51	7.66
TraesCS1B03G0022200	FD1B_omega8_partial_Deletion	Chr1B	6608149	6608199	17.47	6.10	2.86	8.78	2.63	3.34
TraesCS1B03G0022200	FD1B_omega8_partial_Deletion	Chr1B	6608199	6608249	17.00	8.25	2.06	6.20	4.02	1.54
TraesCS1B03G0022200	FD1B_omega8_partial_Deletion	Chr1B	6608249	6608299	13.20	8.25	1.60	7.55	5.31	1.42
TraesCS1B03G0022200	FD1B_omega8_partial_Deletion	Chr1B	6608299	6608349	8.29	8.59	0.97	7.12	4.94	1.44
TraesCS1B03G0022200	FD1B_omega8_partial_Deletion	Chr1B	6608349	6608399	10.76	3.27	3.29	6.35	2.27	2.79

TraesCS1B03G0022200	FD1B_omega8_partial_Deletion	Chr1B	6608399	6608449	8.51	1.84	4.62	4.31	1.00	4.31
TraesCS1B03G0022200	FD1B_omega8_partial_Deletion	Chr1B	6608449	6608499	9.53	2.33	4.08	5.43	0.45	12.04
TraesCS1B03G0022200	FD1B_omega8_partial_Deletion	Chr1B	6608499	6608549	10.47	2.61	4.02	6.57	0.08	83.75
TraesCS1B03G0022200	FD1B_omega8_partial_Deletion	Chr1B	6608549	6608599	11.59	4.12	2.81	6.39	2.25	2.83
TraesCS1B03G0022200	FD1B_omega8_partial_Deletion	Chr1B	6608599	6608649	10.04	5.96	1.68	7.43	4.41	1.68
TraesCS1B03G0022200	FD1B_omega8_partial_Deletion	Chr1B	6608649	6608699	7.51	5.00	1.50	6.94	4.00	1.74
TraesCS1B03G0022200	FD1B_omega8_partial_Deletion	Chr1B	6608699	6608749	8.98	4.75	1.89	8.98	3.75	2.40
TraesCS1B03G0022200	FD1B_omega8_partial_Deletion	Chr1B	6608749	6608799	16.73	5.82	2.87	16.73	4.88	3.43
TraesCS1B03G0022200	FD1B_omega8_partial_Deletion	Chr1B	6608799	6608849	17.43	8.04	2.17	17.10	7.04	2.43
TraesCS1B03G0022200	FD1B_omega8_partial_Deletion	Chr1B	6608849	6608899	14.45	9.59	1.51	13.45	9.18	1.47
TraesCS1D03G0009800	FD1D_gamma1_functional_Deletion	Chr1D	2347407	2347457	20.41	8.86	2.30	20.41	7.59	2.69
TraesCS1D03G0009800	FD1D_gamma1_functional_Deletion	Chr1D	2347457	2347507	18.41	10.31	1.79	17.86	6.86	2.60
TraesCS1D03G0009800	FD1D_gamma1_functional_Deletion	Chr1D	2347507	2347557	15.02	10.33	1.45	13.02	5.76	2.26
TraesCS1D03G0009800	FD1D_gamma1_functional_Deletion	Chr1D	2347557	2347607	13.55	8.84	1.53	10.86	5.65	1.92
TraesCS1D03G0009800	FD1D_gamma1_functional_Deletion	Chr1D	2347607	2347657	16.78	8.98	1.87	14.29	5.22	2.74
TraesCS1D03G0009800	FD1D_gamma1_functional_Deletion	Chr1D	2347657	2347707	15.73	8.98	1.75	14.73	5.00	2.95
TraesCS1D03G0009800	FD1D_gamma1_functional_Deletion	Chr1D	2347707	2347757	20.82	8.71	2.39	20.49	3.12	6.57
TraesCS1D03G0009800	FD1D_gamma1_functional_Deletion	Chr1D	2347757	2347807	24.24	10.10	2.40	24.12	2.41	10.00
TraesCS1D03G0009800	FD1D_gamma1_functional_Deletion	Chr1D	2347807	2347857	26.96	9.31	2.89	25.96	2.90	8.95
TraesCS1D03G0009800	FD1D_gamma1_functional_Deletion	Chr1D	2347857	2347907	23.41	6.12	3.83	22.41	4.06	5.52
TraesCS1D03G0009800	FD1D_gamma1_functional_Deletion	Chr1D	2347907	2347957	25.08	5.39	4.65	24.18	5.06	4.78
TraesCS1D03G0009800	FD1D_gamma1_functional_Deletion	Chr1D	2347957	2348007	25.47	5.78	4.40	25.47	4.78	5.32
TraesCS1D03G0009800	FD1D_gamma1_functional_Deletion	Chr1D	2348007	2348057	26.96	6.73	4.01	26.96	5.73	4.71
TraesCS1D03G0009800	FD1D_gamma1_functional_Deletion	Chr1D	2348057	2348107	29.51	5.57	5.30	29.51	5.00	5.90
TraesCS1D03G0009800	FD1D_gamma1_functional_Deletion	Chr1D	2348107	2348157	37.88	7.02	5.40	37.88	6.80	5.57
TraesCS1D03G0009800	FD1D_gamma1_functional_Deletion	Chr1D	2348157	2348207	36.63	10.39	3.52	36.63	9.39	3.90
TraesCS1D03G0009800	FD1D_gamma1_functional_Deletion	Chr1D	2348207	2348257	32.12	11.98	2.68	31.20	10.98	2.84
TraesCS1D03G0009800	FD1D_gamma1_functional_Deletion	Chr1D	2348257	2348307	30.98	8.57	3.62	29.98	7.88	3.80
TraesCS1D03G0009900	FD1D_gamma2_functional_Intact	Chr1D	2359802	2359852	21.75	8.02	2.71	21.75	8.02	2.71
TraesCS1D03G0009900	FD1D_gamma2_functional_Intact	Chr1D	2359852	2359902	19.55	12.94	1.51	19.55	12.94	1.51
TraesCS1D03G0009900	FD1D_gamma2_functional_Intact	Chr1D	2359902	2359952	19.47	14.37	1.35	18.82	14.37	1.31
TraesCS1D03G0009900	FD1D_gamma2_functional_Intact	Chr1D	2359952	2360002	19.92	13.18	1.51	18.65	13.18	1.42
TraesCS1D03G0009900	FD1D_gamma2_functional_Intact	Chr1D	2360002	2360052	19.94	9.59	2.08	17.94	9.59	1.87
TraesCS1D03G0009900	FD1D_gamma2_functional_Intact	Chr1D	2360052	2360102	15.10	6.41	2.35	13.75	6.33	2.17
TraesCS1D03G0009900	FD1D_gamma2_functional_Intact	Chr1D	2360102	2360152	13.35	9.37	1.42	11.24	7.78	1.44
TraesCS1D03G0009900	FD1D_gamma2_functional_Intact	Chr1D	2360152	2360202	13.24	15.12	0.88	11.24	10.10	1.11
TraesCS1D03G0009900	FD1D_gamma2_functional_Intact	Chr1D	2360202	2360252	14.29	16.96	0.84	12.31	10.49	1.17
TraesCS1D03G0009900	FD1D_gamma2_functional_Intact	Chr1D	2360252	2360302	18.08	14.61	1.24	15.73	9.47	1.66
TraesCS1D03G0009900	FD1D_gamma2_functional_Intact	Chr1D	2360302	2360352	22.96	8.94	2.57	20.73	7.25	2.86
TraesCS1D03G0009900	FD1D_gamma2_functional_Intact	Chr1D	2360352	2360402	24.75	7.98	3.10	23.47	7.31	3.21
TraesCS1D03G0009900	FD1D_gamma2_functional_Intact	Chr1D	2360402	2360452	25.22	7.88	3.20	24.53	6.88	3.56
TraesCS1D03G0009900	FD1D_gamma2_functional_Intact	Chr1D	2360452	2360502	21.27	7.94	2.68	21.27	5.51	3.86
TraesCS1D03G0009900	FD1D_gamma2_functional_Intact	Chr1D	2360502	2360552	19.43	6.69	2.91	19.43	4.25	4.57
TraesCS1D03G0009900	FD1D_gamma2_functional_Intact	Chr1D	2360552	2360602	19.37	5.92	3.27	19.37	3.92	4.94
TraesCS1D03G0009900	FD1D_gamma2_functional_Intact	Chr1D	2360602	2360652	21.45	4.98	4.31	21.20	4.35	4.87
TraesCS1D03G0009900	FD1D_gamma2_functional_Intact	Chr1D	2360652	2360702	23.65	6.88	3.44	22.65	5.88	3.85
TraesCS1D03G0009900	FD1D_gamma2_functional_Intact	Chr1D	2360702	2360752	24.59	7.10	3.46	23.59	5.75	4.11
TraesCS1D03G0009900	FD1D_gamma2_functional_Intact	Chr1D	2360752	2360802	25.35	7.45	3.40	24.59	5.80	4.24
-	FD1D_gamma3_functional_Intact	Chr1D	2372563	2372613	18.27	11.33	1.61	17.88	10.33	1.73
-	FD1D_gamma3_functional_Intact	Chr1D	2372613	2372663	17.25	9.27	1.86	16.53	7.35	2.25
-	FD1D_gamma3_functional_Intact	Chr1D	2372663	2372713	19.47	7.16	2.72	18.47	5.98	3.09

-	FD1D_gamma3_functional_Intact	Chr1D	2372713	2372763	20.10	5.76	3.49	18.25	4.12	4.43
-	FD1D_gamma3_functional_Intact	Chr1D	2372763	2372813	21.06	9.29	2.27	19.88	7.29	2.73
-	FD1D_gamma3_functional_Intact	Chr1D	2372813	2372863	22.18	8.92	2.49	21.18	6.92	3.06
-	FD1D_gamma3_functional_Intact	Chr1D	2372863	2372913	23.49	9.18	2.56	23.31	8.57	2.72
-	FD1D_gamma3_functional_Intact	Chr1D	2372913	2372963	27.57	11.18	2.47	26.67	11.18	2.39
-	FD1D_gamma3_functional_Intact	Chr1D	2372963	2373013	28.57	12.45	2.29	27.57	12.45	2.21
-	FD1D_gamma3_functional_Intact	Chr1D	2373013	2373063	27.86	11.78	2.36	26.86	11.78	2.28
TraesCS1D03G0010000	FD1D_gamma4_functional_Deletion	Chr1D	2377468	2377518	17.51	7.92	2.21	17.51	5.51	3.18
TraesCS1D03G0010000	FD1D_gamma4_functional_Deletion	Chr1D	2377518	2377568	18.94	10.06	1.88	18.94	6.33	2.99
TraesCS1D03G0010000	FD1D_gamma4_functional_Deletion	Chr1D	2377568	2377618	17.59	11.71	1.50	17.59	7.88	2.23
TraesCS1D03G0010000	FD1D_gamma4_functional_Deletion	Chr1D	2377618	2377668	15.73	8.04	1.96	15.22	5.43	2.80
TraesCS1D03G0010000	FD1D_gamma4_functional_Deletion	Chr1D	2377668	2377718	12.57	5.76	2.18	10.57	2.35	4.49
TraesCS1D03G0010000	FD1D_gamma4_functional_Deletion	Chr1D	2377718	2377768	9.96	5.59	1.78	6.96	1.55	4.49
TraesCS1D03G0010000	FD1D_gamma4_functional_Deletion	Chr1D	2377768	2377818	13.18	9.35	1.41	9.02	5.25	1.72
TraesCS1D03G0010000	FD1D_gamma4_functional_Deletion	Chr1D	2377818	2377868	15.92	11.61	1.37	13.14	8.27	1.59
TraesCS1D03G0010000	FD1D_gamma4_functional_Deletion	Chr1D	2377868	2377918	17.22	11.49	1.50	16.88	9.49	1.78
TraesCS1D03G0010000	FD1D_gamma4_functional_Deletion	Chr1D	2377918	2377968	16.43	8.47	1.94	16.43	7.04	2.33
TraesCS1D03G0010000	FD1D_gamma4_functional_Deletion	Chr1D	2377968	2378018	14.90	7.35	2.03	14.90	7.35	2.03
TraesCS1D03G0010000	FD1D_gamma4_functional_Deletion	Chr1D	2378018	2378068	13.94	8.73	1.60	13.94	8.73	1.60
TraesCS1D03G0010000	FD1D_gamma4_functional_Deletion	Chr1D	2378068	2378118	17.02	12.86	1.32	17.02	12.22	1.39
TraesCS1D03G0010000	FD1D_gamma4_functional_Deletion	Chr1D	2378118	2378168	20.53	14.39	1.43	20.10	12.86	1.56
TraesCS1D03G0010000	FD1D_gamma4_functional_Deletion	Chr1D	2378168	2378218	24.88	12.94	1.92	23.88	10.94	2.18
TraesCS1D03G0010000	FD1D_gamma4_functional_Deletion	Chr1D	2378218	2378268	23.55	7.12	3.31	22.55	5.92	3.81
TraesCS1D03G0010000	FD1D_gamma4_functional_Deletion	Chr1D	2378268	2378318	23.75	5.25	4.52	23.14	4.98	4.65
TraesCS1D03G0010000	FD1D_gamma4_functional_Deletion	Chr1D	2378318	2378368	22.59	7.47	3.02	21.59	7.47	2.89
TraesCS1D03G0010000	FD1D_gamma4_functional_Deletion	Chr1D	2378368	2378418	22.14	9.14	2.42	20.14	8.73	2.31
TraesCS1D03G0010000	FD1D_gamma4_functional_Deletion	Chr1D	2378418	2378468	26.92	7.12	3.78	24.00	6.12	3.92
TraesCS1D03G0010400	FD1D_gamma5_functional_Intact	Chr1D	2453767	2453817	16.04	10.22	1.57	16.04	6.92	2.32
TraesCS1D03G0010400	FD1D_gamma5_functional_Intact	Chr1D	2453817	2453867	14.35	12.37	1.16	14.35	8.29	1.73
TraesCS1D03G0010400	FD1D_gamma5_functional_Intact	Chr1D	2453867	2453917	13.96	13.24	1.05	13.96	9.96	1.40
TraesCS1D03G0010400	FD1D_gamma5_functional_Intact	Chr1D	2453917	2453967	18.20	11.73	1.55	17.41	9.49	1.83
TraesCS1D03G0010400	FD1D_gamma5_functional_Intact	Chr1D	2453967	2454017	21.22	10.27	2.06	20.16	9.04	2.23
TraesCS1D03G0010400	FD1D_gamma5_functional_Intact	Chr1D	2454017	2454067	23.31	11.31	2.06	21.02	9.55	2.20
TraesCS1D03G0010400	FD1D_gamma5_functional_Intact	Chr1D	2454067	2454117	17.73	11.90	1.49	15.65	8.88	1.76
TraesCS1D03G0010400	FD1D_gamma5_functional_Intact	Chr1D	2454117	2454167	17.29	11.10	1.56	15.35	8.88	1.73
TraesCS1D03G0010400	FD1D_gamma5_functional_Intact	Chr1D	2454167	2454217	17.86	13.10	1.36	17.16	10.16	1.69
TraesCS1D03G0010400	FD1D_gamma5_functional_Intact	Chr1D	2454217	2454267	18.43	18.69	0.99	18.43	15.63	1.18
TraesCS1D03G0010400	FD1D_gamma5_functional_Intact	Chr1D	2454267	2454317	17.82	19.45	0.92	17.82	16.84	1.06
TraesCS1D03G0010400	FD1D_gamma5_functional_Intact	Chr1D	2454317	2454367	19.49	21.35	0.91	19.49	19.35	1.01
TraesCS1D03G0010400	FD1D_gamma5_functional_Intact	Chr1D	2454367	2454417	19.53	17.76	1.10	19.53	16.37	1.19
TraesCS1D03G0010400	FD1D_gamma5_functional_Intact	Chr1D	2454417	2454467	18.69	18.33	1.02	18.37	16.84	1.09
TraesCS1D03G0010400	FD1D_gamma5_functional_Intact	Chr1D	2454467	2454517	18.75	14.25	1.31	17.75	13.25	1.34
TraesCS1D03G0010400	FD1D_gamma5_functional_Intact	Chr1D	2454517	2454567	21.27	13.94	1.53	20.27	13.20	1.54
TraesCS1D03G0010400	FD1D_gamma5_functional_Intact	Chr1D	2454567	2454617	22.82	12.86	1.77	22.16	12.65	1.75
TraesCS1D03G0010400	FD1D_gamma5_functional_Intact	Chr1D	2454617	2454667	19.33	15.71	1.23	19.33	14.71	1.31
TraesCS1D03G0010500	FD1D_gamma6_functional_Intact	Chr1D	2462565	2462615	17.76	10.65	1.67	16.76	8.78	1.91
TraesCS1D03G0010500	FD1D_gamma6_functional_Intact	Chr1D	2462615	2462665	13.41	13.25	1.01	13.31	11.82	1.13
TraesCS1D03G0010500	FD1D_gamma6_functional_Intact	Chr1D	2462665	2462715	15.96	11.51	1.39	15.06	9.82	1.53
TraesCS1D03G0010500	FD1D_gamma6_functional_Intact	Chr1D	2462715	2462765	19.14	9.35	2.05	15.76	7.00	2.25
TraesCS1D03G0010500	FD1D_gamma6_functional_Intact	Chr1D	2462765	2462815	17.92	8.29	2.16	13.43	4.49	2.99
TraesCS1D03G0010500	FD1D_gamma6_functional_Intact	Chr1D	2462815	2462865	16.49	7.04	2.34	13.31	4.41	3.02

TraesCS1D03G0010500	FD1D_gamma6_functional_Intact	Chr1D	2462865	2462915	16.22	4.75	3.42	15.04	3.29	4.57
TraesCS1D03G0010500	FD1D_gamma6_functional_Intact	Chr1D	2462915	2462965	16.33	5.25	3.11	16.33	4.75	3.44
TraesCS1D03G0010500	FD1D_gamma6_functional_Intact	Chr1D	2462965	2463015	18.18	6.73	2.70	18.18	5.18	3.51
TraesCS1D03G0010500	FD1D_gamma6_functional_Intact	Chr1D	2463015	2463065	17.10	10.90	1.57	16.80	6.80	2.47
TraesCS1D03G0010500	FD1D_gamma6_functional_Intact	Chr1D	2463065	2463115	22.43	12.31	1.82	21.43	7.59	2.82
TraesCS1D03G0010500	FD1D_gamma6_functional_Intact	Chr1D	2463115	2463165	22.06	12.71	1.74	21.06	8.45	2.49
TraesCS1D03G0010500	FD1D_gamma6_functional_Intact	Chr1D	2463165	2463215	22.88	8.65	2.65	22.16	6.78	3.27
TraesCS1D03G0010500	FD1D_gamma6_functional_Intact	Chr1D	2463215	2463265	22.16	8.43	2.63	22.14	6.33	3.50
TraesCS1D03G0010500	FD1D_gamma6_functional_Intact	Chr1D	2463265	2463315	19.65	9.67	2.03	18.65	7.78	2.40
TraesCS1D03G0010500	FD1D_gamma6_functional_Intact	Chr1D	2463315	2463365	21.31	11.24	1.90	20.31	10.00	2.03
TraesCS1D03G0010500	FD1D_gamma6_functional_Intact	Chr1D	2463365	2463415	22.84	8.45	2.70	21.84	7.45	2.93
TraesCS1D03G0010500	FD1D_gamma6_functional_Intact	Chr1D	2463415	2463465	22.22	6.94	3.20	22.18	6.31	3.51
-	FD1D_gamma7_functional_Deletion	Chr1D	2524599	2524649	10.92	6.67	1.64	8.71	5.67	1.54
-	FD1D_gamma7_functional_Deletion	Chr1D	2524649	2524699	13.43	11.08	1.21	12.43	9.80	1.27
-	FD1D_gamma7_functional_Deletion	Chr1D	2524699	2524749	14.86	11.92	1.25	13.86	10.53	1.32
-	FD1D_gamma7_functional_Deletion	Chr1D	2524749	2524799	15.57	8.84	1.76	13.76	7.37	1.87
-	FD1D_gamma7_functional_Deletion	Chr1D	2524799	2524849	15.84	6.08	2.61	10.92	2.75	3.98
-	FD1D_gamma7_functional_Deletion	Chr1D	2524849	2524899	12.82	3.00	4.27	8.02	1.00	8.02
-	FD1D_gamma7_functional_Deletion	Chr1D	2524899	2524949	6.41	2.35	2.72	4.39	0.39	11.20
-	FD1D_gamma7_functional_Deletion	Chr1D	2524949	2524999	3.96	1.76	2.24	2.80	1.00	2.80
-	FD1D_gamma7_functional_Deletion	Chr1D	2524999	2525049	8.90	4.59	1.94	6.10	2.43	2.51
-	FD1D_gamma7_functional_Deletion	Chr1D	2525049	2525099	12.76	6.73	1.90	10.10	3.04	3.32
-	FD1D_gamma7_functional_Deletion	Chr1D	2525099	2525149	10.90	3.08	3.54	8.55	2.67	3.21
-	FD1D_gamma7_functional_Deletion	Chr1D	2525149	2525199	4.35	0.71	6.17	2.82	0.71	4.00
-	FD1D_gamma7_functional_Deletion	Chr1D	2525199	2525249	4.80	2.41	1.99	0.12	0.86	0.14
-	FD1D_gamma7_functional_Deletion	Chr1D	2525249	2525299	5.57	2.80	1.99	1.18	1.00	1.18
-	FD1D_gamma7_functional_Deletion	Chr1D	2525299	2525349	6.94	2.47	2.81	2.63	2.37	1.11
-	FD1D_gamma7_functional_Deletion	Chr1D	2525349	2525399	6.57	2.04	3.22	3.00	2.04	1.47
-	FD1D_gamma7_functional_Deletion	Chr1D	2525399	2525449	6.51	2.61	2.50	2.96	2.22	1.34
-	FD1D_gamma7_functional_Deletion	Chr1D	2525449	2525499	6.47	2.88	2.24	4.45	2.18	2.05
-	FD1D_gamma7_functional_Deletion	Chr1D	2525499	2525549	12.69	4.41	2.88	9.12	2.90	3.14
-	FD1D_gamma7_functional_Deletion	Chr1D	2525549	2525599	15.73	5.37	2.93	12.92	3.73	3.47
-	FD1D_gamma7_functional_Deletion	Chr1D	2525599	2525649	14.86	6.63	2.24	13.73	4.00	3.43
-	FD1D_gamma7_functional_Deletion	Chr1D	2525649	2525699	13.29	5.78	2.30	13.29	4.49	2.96
-	FD1D_gamma7_functional_Deletion	Chr1D	2525699	2525749	14.43	6.75	2.14	14.43	5.75	2.51
TraesCS1D03G0011600	FD1D_gamma8_functional_Deletion	Chr1D	2537250	2537300	3.90	2.86	1.36	2.69	1.14	2.36
TraesCS1D03G0011600	FD1D_gamma8_functional_Deletion	Chr1D	2537300	2537350	2.86	1.84	1.55	2.00	0.00	2.00
TraesCS1D03G0011600	FD1D_gamma8_functional_Deletion	Chr1D	2537350	2537400	4.63	1.41	3.28	2.16	0.00	2.16
TraesCS1D03G0011600	FD1D_gamma8_functional_Deletion	Chr1D	2537400	2537450	7.12	2.04	3.49	4.04	0.00	4.04
TraesCS1D03G0011600	FD1D_gamma8_functional_Deletion	Chr1D	2537450	2537500	9.22	3.49	2.64	5.06	0.00	5.06
TraesCS1D03G0011600	FD1D_gamma8_functional_Deletion	Chr1D	2537500	2537550	8.12	2.75	2.96	3.94	0.00	3.94
TraesCS1D03G0011600	FD1D_gamma8_functional_Deletion	Chr1D	2537550	2537600	9.45	1.31	7.19	5.35	0.14	39.00
TraesCS1D03G0011600	FD1D_gamma8_functional_Deletion	Chr1D	2537600	2537650	7.39	3.49	2.12	5.24	1.98	2.64
TraesCS1D03G0011600	FD1D_gamma8_functional_Deletion	Chr1D	2537650	2537700	5.27	4.10	1.29	4.37	2.00	2.19
TraesCS1D03G0011600	FD1D_gamma8_functional_Deletion	Chr1D	2537700	2537750	5.10	1.92	2.65	4.37	0.37	11.74
TraesCS1D03G0011600	FD1D_gamma8_functional_Deletion	Chr1D	2537750	2537800	4.96	1.31	3.78	2.45	0.00	2.45
TraesCS1D03G0011600	FD1D_gamma8_functional_Deletion	Chr1D	2537800	2537850	5.86	2.08	2.82	3.00	0.00	3.00
TraesCS1D03G0011600	FD1D_gamma8_functional_Deletion	Chr1D	2537850	2537900	5.37	3.33	1.61	2.16	2.14	1.01
TraesCS1D03G0011600	FD1D_gamma8_functional_Deletion	Chr1D	2537900	2537950	6.63	4.00	1.66	3.73	4.00	0.93
TraesCS1D03G0011600	FD1D_gamma8_functional_Deletion	Chr1D	2537950	2538000	9.51	4.92	1.93	9.20	4.92	1.87
TraesCS1D03G0011600	FD1D_gamma8_functional_Deletion	Chr1D	2538000	2538050	12.43	5.96	2.09	12.43	5.29	2.35

TraesCS1D03G0011600	FD1D_gamma8_functional_Deletion	Chr1D	2538050	2538100	16.39	8.08	2.03	14.51	5.76	2.52
TraesCS1D03G0011600	FD1D_gamma8_functional_Deletion	Chr1D	2538100	2538150	20.63	14.22	1.45	16.63	9.14	1.82
TraesCS1D03G0011600	FD1D_gamma8_functional_Deletion	Chr1D	2538150	2538200	23.88	12.65	1.89	21.25	9.02	2.36
TraesCS1D03G0011600	FD1D_gamma8_functional_Deletion	Chr1D	2538200	2538250	27.73	11.88	2.33	26.24	10.39	2.52
TraesCS1D03G0011700	FD1D_gamma9_functional_Deletion	Chr1D	2568276	2568326	13.69	11.16	1.23	12.90	9.43	1.37
TraesCS1D03G0011700	FD1D_gamma9_functional_Deletion	Chr1D	2568326	2568376	13.69	8.08	1.69	11.67	5.59	2.09
TraesCS1D03G0011700	FD1D_gamma9_functional_Deletion	Chr1D	2568376	2568426	13.25	6.12	2.17	10.08	1.65	6.12
TraesCS1D03G0011700	FD1D_gamma9_functional_Deletion	Chr1D	2568426	2568476	16.73	5.33	3.14	11.02	2.06	5.35
TraesCS1D03G0011700	FD1D_gamma9_functional_Deletion	Chr1D	2568476	2568526	17.31	8.65	2.00	12.18	3.90	3.12
TraesCS1D03G0011700	FD1D_gamma9_functional_Deletion	Chr1D	2568526	2568576	15.96	7.20	2.22	10.10	3.18	3.18
TraesCS1D03G0011700	FD1D_gamma9_functional_Deletion	Chr1D	2568576	2568626	10.27	6.75	1.52	6.75	2.24	3.02
TraesCS1D03G0011700	FD1D_gamma9_functional_Deletion	Chr1D	2568626	2568676	9.04	6.61	1.37	6.10	2.00	3.05
TraesCS1D03G0011700	FD1D_gamma9_functional_Deletion	Chr1D	2568676	2568726	11.98	6.24	1.92	7.45	1.45	5.14
TraesCS1D03G0011700	FD1D_gamma9_functional_Deletion	Chr1D	2568726	2568776	10.57	2.82	3.74	6.47	0.53	12.22
TraesCS1D03G0011700	FD1D_gamma9_functional_Deletion	Chr1D	2568776	2568826	12.24	2.16	5.67	5.75	0.90	6.37
TraesCS1D03G0011700	FD1D_gamma9_functional_Deletion	Chr1D	2568826	2568876	12.24	1.86	6.57	3.18	1.00	3.18
TraesCS1D03G0011700	FD1D_gamma9_functional_Deletion	Chr1D	2568876	2568926	6.76	1.92	3.52	3.00	0.98	3.06
TraesCS1D03G0011700	FD1D_gamma9_functional_Deletion	Chr1D	2568926	2568976	5.22	1.14	4.59	3.73	0.00	3.73
TraesCS1D03G0011700	FD1D_gamma9_functional_Deletion	Chr1D	2568976	2569026	8.76	0.55	15.96	3.59	0.00	3.59
TraesCS1D03G0011700	FD1D_gamma9_functional_Deletion	Chr1D	2569026	2569076	13.35	1.33	10.01	2.75	0.76	3.59
TraesCS1D03G0011700	FD1D_gamma9_functional_Deletion	Chr1D	2569076	2569126	11.80	2.31	5.10	3.69	1.00	3.69
TraesCS1D03G0011700	FD1D_gamma9_functional_Deletion	Chr1D	2569126	2569176	10.06	2.47	4.07	2.18	0.02	111.00
TraesCS1D03G0011700	FD1D_gamma9_functional_Deletion	Chr1D	2569176	2569226	9.88	3.00	3.29	2.22	0.00	2.22
TraesCS1D03G0011700	FD1D_gamma9_functional_Deletion	Chr1D	2569226	2569276	8.57	1.75	4.91	3.00	0.00	3.00
TraesCS1D03G0011700	FD1D_gamma9_functional_Deletion	Chr1D	2569276	2569326	8.27	0.43	19.18	1.59	0.00	1.59
TraesCS1D03G0011700	FD1D_gamma9_functional_Deletion	Chr1D	2569326	2569376	8.61	0.65	13.30	2.41	0.00	2.41
TraesCS1D03G0011700	FD1D_gamma9_functional_Deletion	Chr1D	2569376	2569426	6.29	1.86	3.38	2.96	1.65	1.80
TraesCS1D03G0011700	FD1D_gamma9_functional_Deletion	Chr1D	2569426	2569476	9.53	4.71	2.03	3.98	4.00	1.00
TraesCS1D03G0011700	FD1D_gamma9_functional_Deletion	Chr1D	2569476	2569526	11.02	7.02	1.57	5.31	4.16	1.28
TraesCS1D03G0011700	FD1D_gamma9_functional_Deletion	Chr1D	2569526	2569576	13.41	7.51	1.79	9.18	4.47	2.05
TraesCS1D03G0011700	FD1D_gamma9_functional_Deletion	Chr1D	2569576	2569626	21.65	8.86	2.44	15.18	5.65	2.69
TraesCS1D03G0011700	FD1D_gamma9_functional_Deletion	Chr1D	2569626	2569676	23.76	14.78	1.61	22.65	11.63	1.95
TraesCS1D03G0011700	FD1D_gamma9_functional_Deletion	Chr1D	2569676	2569726	24.59	13.24	1.86	24.59	11.33	2.17
TraesCS1D03G0011700	FD1D_gamma9_functional_Deletion	Chr1D	2569726	2569776	22.08	13.41	1.65	22.08	11.41	1.93
TraesCS1A03G0015500	FD1D_omega1_functional_Deletion	Chr1D	2111630	2111680	20.35	15.94	1.28	20.35	14.94	1.36
TraesCS1A03G0015500	FD1D_omega1_functional_Deletion	Chr1D	2111680	2111730	20.25	10.47	1.93	20.25	9.51	2.13
TraesCS1A03G0015500	FD1D_omega1_functional_Deletion	Chr1D	2111730	2111780	24.12	8.24	2.93	24.12	7.27	3.32
TraesCS1A03G0015500	FD1D_omega1_functional_Deletion	Chr1D	2111780	2111830	19.78	10.92	1.81	19.78	8.61	2.30
TraesCS1A03G0015500	FD1D_omega1_functional_Deletion	Chr1D	2111830	2111880	18.98	11.22	1.69	18.02	8.37	2.15
TraesCS1A03G0015500	FD1D_omega1_functional_Deletion	Chr1D	2111880	2111930	16.67	9.43	1.77	14.67	6.82	2.15
TraesCS1A03G0015500	FD1D_omega1_functional_Deletion	Chr1D	2111930	2111980	15.10	6.80	2.22	13.76	4.39	3.13
TraesCS1A03G0015500	FD1D_omega1_functional_Deletion	Chr1D	2111980	2112030	17.02	4.22	4.04	15.84	2.65	5.99
TraesCS1A03G0015500	FD1D_omega1_functional_Deletion	Chr1D	2112030	2112080	16.73	0.73	23.05	14.71	0.10	150.00
TraesCS1A03G0015500	FD1D_omega1_functional_Deletion	Chr1D	2112080	2112130	12.67	0.00	12.67	10.37	0.00	10.37
TraesCS1A03G0015500	FD1D_omega1_functional_Deletion	Chr1D	2112130	2112180	9.04	0.73	12.46	6.25	0.45	13.87
TraesCS1A03G0015500	FD1D_omega1_functional_Deletion	Chr1D	2112180	2112230	8.76	2.39	3.66	5.90	1.20	4.93
TraesCS1A03G0015500	FD1D_omega1_functional_Deletion	Chr1D	2112230	2112280	8.00	5.18	1.55	6.47	2.00	3.24
TraesCS1A03G0015500	FD1D_omega1_functional_Deletion	Chr1D	2112280	2112330	13.55	5.80	2.33	8.39	1.29	6.48
TraesCS1A03G0015500	FD1D_omega1_functional_Deletion	Chr1D	2112330	2112380	13.20	4.04	3.27	6.55	0.71	9.28
TraesCS1A03G0015500	FD1D_omega1_functional_Deletion	Chr1D	2112380	2112430	6.96	1.57	4.44	3.35	0.00	3.35
TraesCS1A03G0015500	FD1D_omega1_functional_Deletion	Chr1D	2112430	2112480	8.92	0.00	8.92	4.04	0.00	4.04

TraesCS1A03G0015500	FD1D_omega1_functional_Deletion	Chr1D	2112480	2112530	11.96	0.00	11.96	7.82	0.00	7.82
TraesCS1A03G0015500	FD1D_omega1_functional_Deletion	Chr1D	2112530	2112580	10.75	0.80	13.37	8.86	0.80	11.02
TraesCS1A03G0015500	FD1D_omega1_functional_Deletion	Chr1D	2112580	2112630	12.65	4.61	2.74	10.12	4.41	2.29
TraesCS1A03G0015500	FD1D_omega1_functional_Deletion	Chr1D	2112630	2112680	16.61	7.86	2.11	15.00	6.00	2.50
TraesCS1A03G0015500	FD1D_omega1_functional_Deletion	Chr1D	2112680	2112730	20.53	7.29	2.81	19.45	5.29	3.67
TraesCS1A03G0015500	FD1D_omega1_functional_Deletion	Chr1D	2112730	2112780	22.71	6.78	3.35	21.71	5.08	4.27
TraesCS1D03G0008900	FD1D_omega2_functional_Deletion	Chr1D	2132327	2132377	25.27	3.08	8.21	0.00	0.00	0.00
TraesCS1D03G0008900	FD1D_omega2_functional_Deletion	Chr1D	2132377	2132427	22.49	3.88	5.79	0.00	0.00	0.00
TraesCS1D03G0008900	FD1D_omega2_functional_Deletion	Chr1D	2132427	2132477	17.29	5.08	3.41	0.00	0.00	0.00
TraesCS1D03G0008900	FD1D_omega2_functional_Deletion	Chr1D	2132477	2132527	11.18	5.53	2.02	0.00	0.00	0.00
TraesCS1D03G0008900	FD1D_omega2_functional_Deletion	Chr1D	2132527	2132577	10.94	5.51	1.99	0.00	0.00	0.00
TraesCS1D03G0008900	FD1D_omega2_functional_Deletion	Chr1D	2132577	2132627	8.71	5.08	1.71	0.00	0.00	0.00
TraesCS1D03G0008900	FD1D_omega2_functional_Deletion	Chr1D	2132627	2132677	10.71	6.00	1.78	0.00	0.00	0.00
TraesCS1D03G0008900	FD1D_omega2_functional_Deletion	Chr1D	2132677	2132727	10.59	5.41	1.96	0.00	0.00	0.00
TraesCS1D03G0008900	FD1D_omega2_functional_Deletion	Chr1D	2132727	2132777	13.25	1.20	11.08	0.00	0.00	0.00
TraesCS1D03G0008900	FD1D_omega2_functional_Deletion	Chr1D	2132777	2132827	10.90	0.71	15.44	0.00	0.00	0.00
TraesCS1D03G0008900	FD1D_omega2_functional_Deletion	Chr1D	2132827	2132877	6.78	1.45	4.68	0.00	0.00	0.00
TraesCS1D03G0008900	FD1D_omega2_functional_Deletion	Chr1D	2132877	2132927	6.16	4.94	1.25	0.00	0.00	0.00
TraesCS1D03G0008900	FD1D_omega2_functional_Deletion	Chr1D	2132927	2132977	5.75	3.08	1.87	0.00	0.00	0.00
TraesCS1D03G0008900	FD1D_omega2_functional_Deletion	Chr1D	2132977	2133027	6.33	1.43	4.42	0.00	0.00	0.00
TraesCS1D03G0008900	FD1D_omega2_functional_Deletion	Chr1D	2133027	2133077	5.12	1.86	2.75	0.00	0.00	0.00
TraesCS1D03G0008900	FD1D_omega2_functional_Deletion	Chr1D	2133077	2133127	5.55	2.88	1.93	0.00	0.00	0.00
TraesCS1D03G0008900	FD1D_omega2_functional_Deletion	Chr1D	2133127	2133177	11.96	5.73	2.09	0.00	0.00	0.00
TraesCS1D03G0008900	FD1D_omega2_functional_Deletion	Chr1D	2133177	2133227	13.98	7.61	1.84	0.00	0.00	0.00
TraesCS1D03G0008900	FD1D_omega2_functional_Deletion	Chr1D	2133227	2133277	12.88	5.51	2.34	0.00	0.00	0.00
TraesCS1D03G0008900	FD1D_omega2_functional_Deletion	Chr1D	2133277	2133327	7.45	4.84	1.54	0.00	0.00	0.00
TraesCS1D03G0008900	FD1D_omega2_functional_Deletion	Chr1D	2133327	2133377	4.78	3.47	1.38	0.00	0.00	0.00
TraesCS1D03G0008900	FD1D_omega2_functional_Deletion	Chr1D	2133377	2133427	6.25	7.80	0.80	0.00	0.00	0.00
TraesCS1D03G0008900	FD1D_omega2_functional_Deletion	Chr1D	2133427	2133477	11.78	10.76	1.09	0.00	0.00	0.00
TraesCS1D03G0008900	FD1D_omega2_functional_Deletion	Chr1D	2133477	2133527	17.75	13.90	1.28	0.00	0.00	0.00
TraesCSU03G0557400_1	FD1D_omega3_functional_Deletion	Chr1D	2146244	2146294	20.90	5.94	3.52	0.00	0.00	0.00
TraesCSU03G0557400_1	FD1D_omega3_functional_Deletion	Chr1D	2146294	2146344	18.92	4.06	4.66	0.00	0.00	0.00
TraesCSU03G0557400_1	FD1D_omega3_functional_Deletion	Chr1D	2146344	2146394	16.88	3.20	5.28	0.00	0.00	0.00
TraesCSU03G0557400_1	FD1D_omega3_functional_Deletion	Chr1D	2146394	2146444	19.78	2.86	6.91	0.00	0.00	0.00
TraesCSU03G0557400_1	FD1D_omega3_functional_Deletion	Chr1D	2146444	2146494	19.61	1.24	15.87	0.00	0.00	0.00
TraesCSU03G0557400_1	FD1D_omega3_functional_Deletion	Chr1D	2146494	2146544	14.88	1.00	14.88	0.00	0.00	0.00
TraesCSU03G0557400_1	FD1D_omega3_functional_Deletion	Chr1D	2146544	2146594	9.08	0.12	77.17	0.00	0.00	0.00
TraesCSU03G0557400_1	FD1D_omega3_functional_Deletion	Chr1D	2146594	2146644	9.53	0.67	14.29	0.00	0.00	0.00
TraesCSU03G0557400_1	FD1D_omega3_functional_Deletion	Chr1D	2146644	2146694	13.61	1.14	11.97	0.00	0.00	0.00
TraesCSU03G0557400_1	FD1D_omega3_functional_Deletion	Chr1D	2146694	2146744	13.75	0.73	18.95	0.00	0.00	0.00
TraesCSU03G0557400_1	FD1D_omega3_functional_Deletion	Chr1D	2146744	2146794	11.84	0.45	26.26	0.00	0.00	0.00
TraesCSU03G0557400_1	FD1D_omega3_functional_Deletion	Chr1D	2146794	2146844	10.57	1.00	10.57	0.00	0.00	0.00
TraesCSU03G0557400_1	FD1D_omega3_functional_Deletion	Chr1D	2146844	2146894	11.69	0.76	15.28	0.00	0.00	0.00
TraesCSU03G0557400_1	FD1D_omega3_functional_Deletion	Chr1D	2146894	2146944	9.94	1.67	5.96	0.00	0.00	0.00
TraesCSU03G0557400_1	FD1D_omega3_functional_Deletion	Chr1D	2146944	2146994	7.51	1.31	5.72	0.00	0.00	0.00
TraesCSU03G0557400_1	FD1D_omega3_functional_Deletion	Chr1D	2146994	2147044	12.49	3.00	4.16	0.00	0.00	0.00
TraesCSU03G0557400_1	FD1D_omega3_functional_Deletion	Chr1D	2147044	2147094	20.12	1.98	10.16	0.00	0.00	0.00
TraesCSU03G0557400_1	FD1D_omega3_functional_Deletion	Chr1D	2147094	2147144	19.80	1.00	19.80	0.00	0.00	0.00
TraesCSU03G0557400_1	FD1D_omega3_functional_Deletion	Chr1D	2147144	2147194	14.94	0.08	190.50	0.00	0.00	0.00
TraesCSU03G0557400_1	FD1D_omega3_functional_Deletion	Chr1D	2147194	2147244	14.25	3.12	4.57	0.00	0.00	0.00
TraesCSU03G0557400_1	FD1D_omega3_functional_Deletion	Chr1D	2147244	2147294	15.59	7.47	2.09	0.00	0.00	0.00

TraesCSU03G0557400_1	FD1D_omega3_functional_Deletion	Chr1D	2147294	2147344	16.43	8.57	1.92	0.00	0.00	0.00
TraesCSU03G0557400_1	FD1D_omega3_functional_Deletion	Chr1D	2147344	2147394	15.80	5.12	3.09	0.00	0.00	0.00
TraesCSU03G0557400_1	FD1D_omega3_functional_Deletion	Chr1D	2147394	2147444	15.47	4.37	3.54	0.00	0.00	0.00
TraesCSU03G0557400	FD1D_omega4_functional_Deletion	Chr1D	2169056	2169106	13.84	6.47	2.14	0.00	0.00	0.00
TraesCSU03G0557400	FD1D_omega4_functional_Deletion	Chr1D	2169106	2169156	11.55	5.06	2.28	0.00	0.00	0.00
TraesCSU03G0557400	FD1D_omega4_functional_Deletion	Chr1D	2169156	2169206	14.35	2.31	6.20	0.00	0.00	0.00
TraesCSU03G0557400	FD1D_omega4_functional_Deletion	Chr1D	2169206	2169256	14.12	2.55	5.54	0.00	0.00	0.00
TraesCSU03G0557400	FD1D_omega4_functional_Deletion	Chr1D	2169256	2169306	13.88	3.12	4.45	0.00	0.00	0.00
TraesCSU03G0557400	FD1D_omega4_functional_Deletion	Chr1D	2169306	2169356	9.24	4.65	1.99	0.00	0.00	0.00
TraesCSU03G0557400	FD1D_omega4_functional_Deletion	Chr1D	2169356	2169406	7.82	4.45	1.76	0.00	0.00	0.00
TraesCSU03G0557400	FD1D_omega4_functional_Deletion	Chr1D	2169406	2169456	12.37	3.24	3.82	0.00	0.00	0.00
TraesCSU03G0557400	FD1D_omega4_functional_Deletion	Chr1D	2169456	2169506	13.86	2.37	5.84	0.00	0.00	0.00
TraesCSU03G0557400	FD1D_omega4_functional_Deletion	Chr1D	2169506	2169556	14.90	0.08	190.00	0.00	0.00	0.00
TraesCSU03G0557400	FD1D_omega4_functional_Deletion	Chr1D	2169556	2169606	11.98	0.92	13.00	0.00	0.00	0.00
TraesCSU03G0557400	FD1D_omega4_functional_Deletion	Chr1D	2169606	2169656	11.20	3.59	3.12	0.00	0.00	0.00
TraesCSU03G0557400	FD1D_omega4_functional_Deletion	Chr1D	2169656	2169706	9.71	4.94	1.96	0.00	0.00	0.00
TraesCSU03G0557400	FD1D_omega4_functional_Deletion	Chr1D	2169706	2169756	11.10	3.00	3.70	0.00	0.00	0.00
TraesCSU03G0557400	FD1D_omega4_functional_Deletion	Chr1D	2169756	2169806	9.80	2.18	4.50	0.00	0.00	0.00
TraesCSU03G0557400	FD1D_omega4_functional_Deletion	Chr1D	2169806	2169856	9.80	4.06	2.42	0.00	0.00	0.00
TraesCSU03G0557400	FD1D_omega4_functional_Deletion	Chr1D	2169856	2169906	10.06	6.16	1.63	0.00	0.00	0.00
TraesCSU03G0557400	FD1D_omega4_functional_Deletion	Chr1D	2169906	2169956	9.53	4.59	2.08	0.00	0.00	0.00
TraesCSU03G0557400	FD1D_omega4_functional_Deletion	Chr1D	2169956	2170006	12.04	5.98	2.01	0.00	0.00	0.00
TraesCSU03G0557400	FD1D_omega4_functional_Deletion	Chr1D	2170006	2170056	15.27	5.61	2.72	0.00	0.00	0.00
TraesCSU03G0557400	FD1D_omega4_functional_Deletion	Chr1D	2170056	2170106	15.12	5.84	2.59	0.00	0.00	0.00
TraesCSU03G0557400	FD1D_omega4_functional_Deletion	Chr1D	2170106	2170156	15.92	5.61	2.84	0.00	0.00	0.00
TraesCSU03G0557400	FD1D_omega4_functional_Deletion	Chr1D	2170156	2170206	15.29	5.92	2.58	0.00	0.00	0.00
TraesCSU03G0557400	FD1D_omega4_functional_Deletion	Chr1D	2170206	2170256	19.88	5.75	3.46	0.00	0.00	0.00
-	FD1D_omega5_functional_Deletion	Chr1D	2182973	2183023	17.00	0.20	86.70	1.80	0.12	15.33
-	FD1D_omega5_functional_Deletion	Chr1D	2183023	2183073	17.37	4.43	3.92	5.73	4.43	1.29
-	FD1D_omega5_functional_Deletion	Chr1D	2183073	2183123	17.65	7.43	2.37	11.65	7.43	1.57
-	FD1D_omega5_functional_Deletion	Chr1D	2183123	2183173	15.76	10.06	1.57	12.25	8.78	1.40
-	FD1D_omega5_functional_Deletion	Chr1D	2183173	2183223	20.45	8.24	2.48	9.53	3.96	2.41
-	FD1D_omega5_functional_Deletion	Chr1D	2183223	2183273	17.24	3.63	4.75	3.73	0.86	4.32
-	FD1D_omega5_functional_Deletion	Chr1D	2183273	2183323	14.33	2.49	5.76	4.18	0.00	4.18
-	FD1D_omega5_functional_Deletion	Chr1D	2183323	2183373	15.00	3.41	4.40	5.84	0.00	5.84
-	FD1D_omega5_functional_Deletion	Chr1D	2183373	2183423	14.69	1.00	14.69	6.63	0.00	6.63
-	FD1D_omega5_functional_Deletion	Chr1D	2183423	2183473	15.86	0.82	19.26	4.82	0.00	4.82
-	FD1D_omega5_functional_Deletion	Chr1D	2183473	2183523	15.76	1.47	10.72	1.88	0.00	1.88
-	FD1D_omega5_functional_Deletion	Chr1D	2183523	2183573	12.61	2.00	6.30	0.00	0.00	0.00
-	FD1D_omega5_functional_Deletion	Chr1D	2183573	2183623	9.78	1.84	5.31	0.00	0.00	0.00
-	FD1D_omega5_functional_Deletion	Chr1D	2183623	2183673	9.49	3.02	3.14	0.27	0.00	0.27
-	FD1D_omega5_functional_Deletion	Chr1D	2183673	2183723	13.02	3.90	3.34	3.10	0.00	3.10
-	FD1D_omega5_functional_Deletion	Chr1D	2183723	2183773	14.76	4.45	3.32	4.59	0.00	4.59
-	FD1D_omega5_functional_Deletion	Chr1D	2183773	2183823	14.80	1.94	7.63	4.73	0.00	4.73
-	FD1D_omega5_functional_Deletion	Chr1D	2183823	2183873	12.45	1.86	6.68	2.51	0.00	2.51
-	FD1D_omega5_functional_Deletion	Chr1D	2183873	2183923	14.76	3.12	4.74	1.51	0.00	1.51
-	FD1D_omega5_functional_Deletion	Chr1D	2183923	2183973	15.31	3.71	4.13	4.24	0.00	4.24
-	FD1D_omega5_functional_Deletion	Chr1D	2183973	2184023	18.37	1.29	14.20	10.67	0.00	10.67
-	FD1D_omega5_functional_Deletion	Chr1D	2184023	2184073	26.75	0.37	71.79	15.45	0.00	15.45
-	FD1D_omega5_functional_Deletion	Chr1D	2184073	2184123	26.98	1.00	26.98	13.37	0.00	13.37

* The null distribution of the depth of read coverage ratios was calculated in 50 bp windows for all genes of Chr1A, Chr1B, and Chr1D liftoffed from CS2.1 to the Fielder genome. The 95th percentile of ratio distribution was 4.78 for reads mapped using default settings, and 5.55 for reads filtered using criteria described in the Methods. The ratio values exceeding these thresholds are highlighted in gray.

** Reads filtered by the samtools using the following parameters -q 20 [AS]>80 [NM]<2, that exclude ones with mapping quality < 20, overlapping length < 80 bp, and mismatches > 2.
