# Supplementary material for: CRISPR‐based editing of the ω‐ and γ‐gliadin gene clusters reduces wheat immunoreactivity without affecting grain protein quality
Source: Plant Biotechnol J. 2023 Nov 17;22(4):892–903. doi: 10.1111/pbi.14231 (PMC10955484; doi:10.1111/pbi.14231)
Supplement: Supplementary file 1 — Figure S1 The distributions of the 17 toxic epitopes including 11 binding to R5 mAb and six binding to G12 mAb across all three gliadin subtypes of cultivars Kariega, Chinese Spring and LongReach Lancer. Figure S2 PCR‐based screening of fragment deletions in five functional ω‐gliadin gene copies in T1 generation plants. Figure S3 PCR‐based screening of fragment deletions in five functional ω‐gliadin gene copies in T2 generation plants. Figure S4 NGS‐based detection for fragment deletions in the ω‐gliadin genes of edited line 387‐3‐6 using PCR amplicons. Figure S5 Protein profiles of the non‐edited transgenic line and cultivar Fielder. Figure S6 Mixograph curves of dough developed from the flours of non‐edited transgenic line and edited line 387‐3‐6. Table S1 Number of R5 and G12 mAbs binding toxic epitopes detected within the gliadin‐encoding genes from four wheat cultivars including Fielder, Kariega, Chinese Spring and LongReach Lancer. Table S2 Potential gRNA target sites within the gliadin genes (external Excel file). Table S6 Summary of gene editing events detected by whole genome sequencing in the ω‐ and γ‐gliadin gene clusters and the coordinates of gliadin genes in published Fielder genome (external Excel file). Table S7 The ratio of read coverage calculated by dividing the depth of read coverage in non‐edited line 387‐1‐8 to the depth of read coverage of edited line 387‐3‐6. The depth of read coverage was calculated in the 50 bp windows within each gliadin gene models (External file). Table S8 Raw data for generating figures to show the impacts of gene editing on the content of each gliadin subtype, parameters of protein extracts correlated with grain protein quality for breadmaking and immunoreactivity in Figure 3, and the table to show the impacts of gene editing on dough quality in Table S9 (external Excel file). Table S3 List of PCR primers used in the study. Table S4 Primers for PCR‐based screening of fragment deletions. Table S5 NGS‐based detection for [file PBI-22-892-s001.zip › pbi14231-sup-0005-AppendixS1.docx]

**Supplementary Information**

**Supplementary Figures**


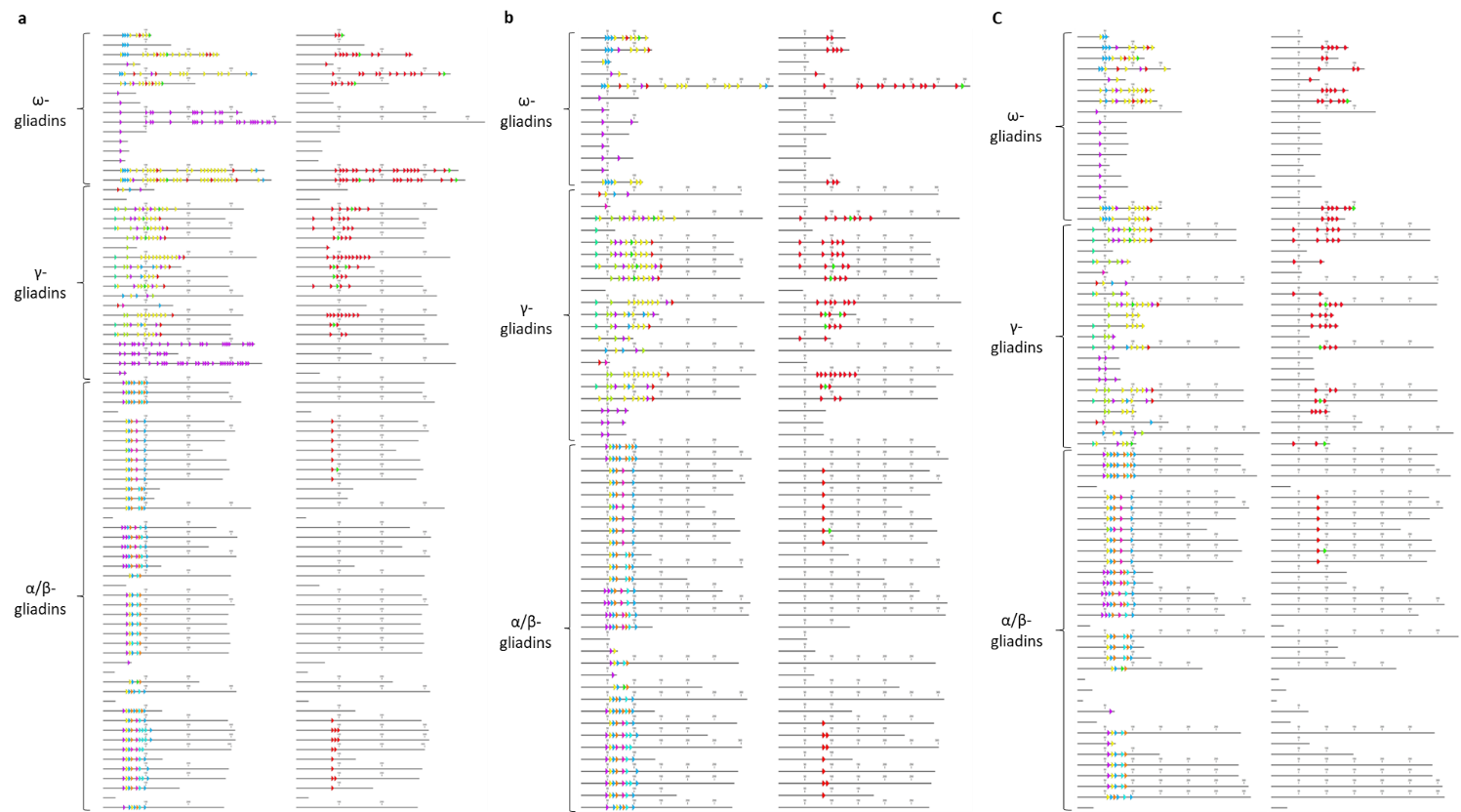


**Supplementary Figure 1.** The distributions of the 17 toxic epitopes including 11 binding to R5 mAb and six binding to G12 mAb across all three gliadin subtypes of cultivars Kariega, Chinese Spring and LongReach Lancer. The 17 toxic epitopes were indicated by the arrows with corresponding color shown in Fig. 1a. The left and right graphs in the panels **a, b** and **c** were, respectively, referred to the distributions of the toxic epitopes binding to R5 and G12 mAbs for cv. Karriega (**a**), cv. Chinese Spring (**b**), and cv. LongReach Lancer (**c**). The number of toxic epitopes binding to R5 and G12 mAbs across all three gliadin subtypes of each cultivar are shown in Supplementary Table 1.

**
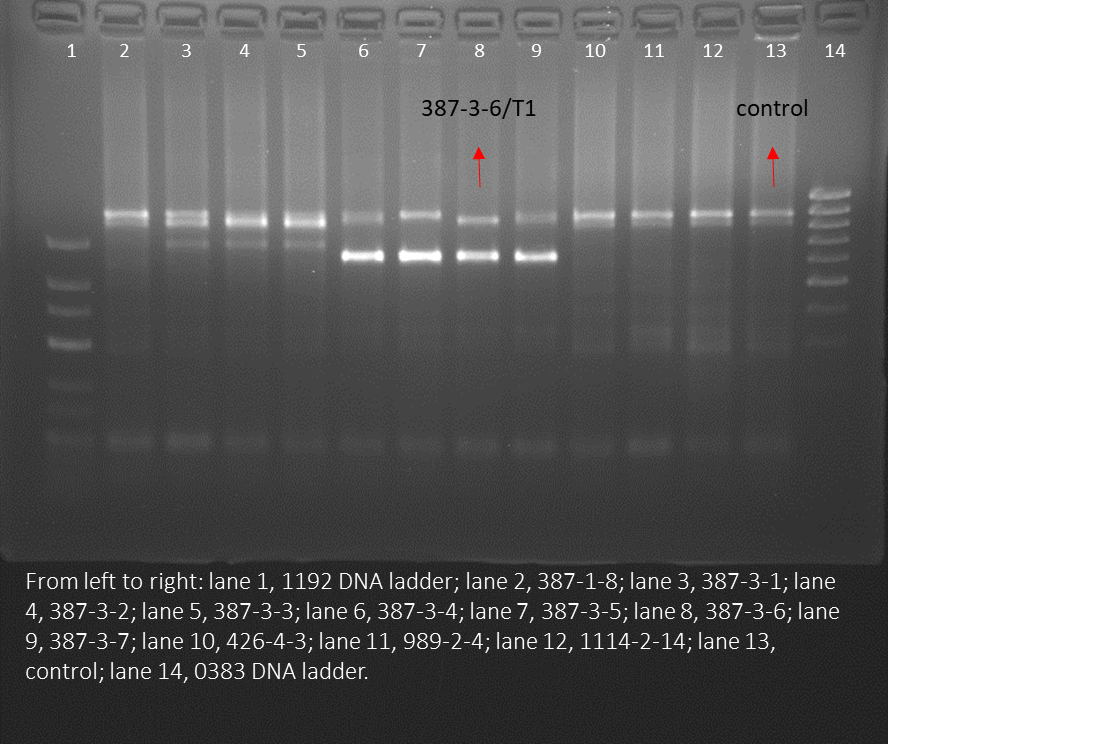
**

**Supplementary Figure 2.** PCR-based screening of fragment deletions in five functional ω-gliadin gene copies from chromosomes 1A and 1D in the T_1_ population using primer pair 1A1DmiseqF-1&1A1DmiseqR-4. lane 1: 1192 DNA ladder; lane 2: 387-1-8; lane 3: 387-3-1; lane 4: 387-3-2; lane 5: 387-3-3; lane 6: 387-3-4; lane 7: 387-3-5; lane 8: 387-3-6; lane 9: 387-3-7; lane 10: 426-4-3; lane 11: 989-2-4; lane 12: 1114-2-14; lane 13: control (wild-type cv. Fielder); lane 14: 0383 DNA ladder; the line 387-3-6/T_1_ and control were indicated by red arrow in lane 8 and 13, respectively. The expected size of PCR products for the control are 791-bp fragment from FD1A_omega1, 872-bp fragment from FD1A_omega3, 830-bp fragment from FD1D_omega1, and 854-bp fragment from other four gene copies in chromosome 1D. The amplified products from seven T_1_ plants derived from T_0_ line 387-3 including plants 387-3-1, 387-3-2, 387-3-3, 387-3-4, 387-3-5, 387-3-6, 387-3-7 had fragments with smaller size than the control, indicating there were fragment deletions in the ω-gliadin gene copies located on chromosomes 1A and 1D of theses seven T_1_ plants. Among them, the amplified fragments from T_1_ plant 387-3-6 in lane 8 had two bands, which were both smaller than the control, indicating that both were amplified from the edited gene copies.

**Supplementary Figure 3.** PCR-based screening of fragment deletions in five functional ω-gliadin gene copies located on chromosomes 1A and 1D in the T_2_ population including 183 lines derived from plant 387-3-6 (A-D). PCR was performed using primer pair 1A1DmiseqF-1&1A1DmiseqR-4. The 0383 DNA ladder was loaded into the twenty-fifth lane of each row. The control represented by wild-type cv. Fielder was marked by the red arrow. The amplified fragments from T_2_ plants 1-48, 49-95, 97-144, 145-183 and 96 were shown in the four rows from top to bottom. The amplified fragments from each T_2_ plant had two bands of smaller size than the control, indicating both were amplified from the edited gene copies. There was no segregation in the fragment deletions observed among the analyzed 183 lines, revealing that the T_1_ line 387-3-6 is homozygous for the editing events.


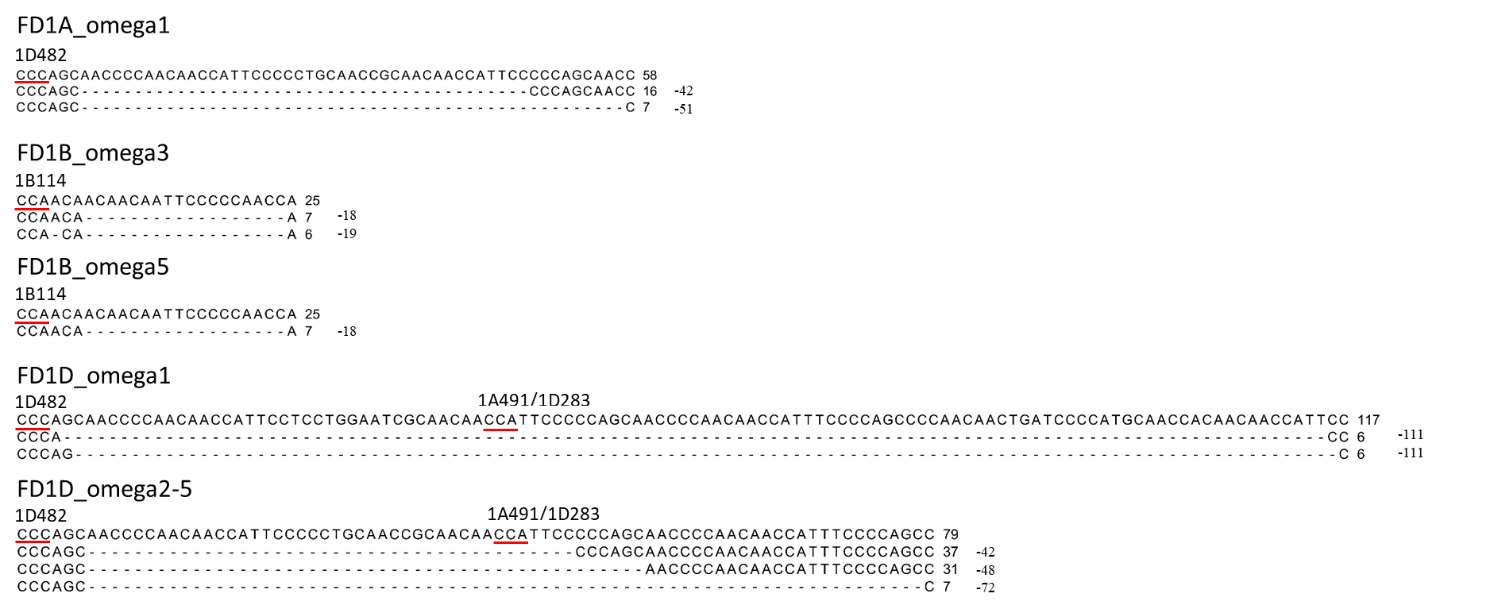
**Supplementary Figure 4.** NGS-based detection for fragment deletions in the ω-gliadin genes of edited line 387-3-6 using PCR amplicons produced by primer pairs 1A1DmiseqF-1&1A1DmiseqR-1 and 1BmiseqF-2&1BmiseqR-2. The PAM site NGG is underlined. The deleted bases were shown by black dots. The sizes of the deleted fragments are shown as negative number at the end of sequence. The sizes of the deleted fragments in FD1A_omega3 were not detected within a total of 17 reads using the PCR amplicons based NGS, mostly due to the occurrence of the long fragment deletion resulting in the targeted amplified region being deleted (Fig. 2, Supplementary Table 6). A summary of the editing events detected by PCR amplicons based NGS in each copy was shown in Supplementary Table 5.


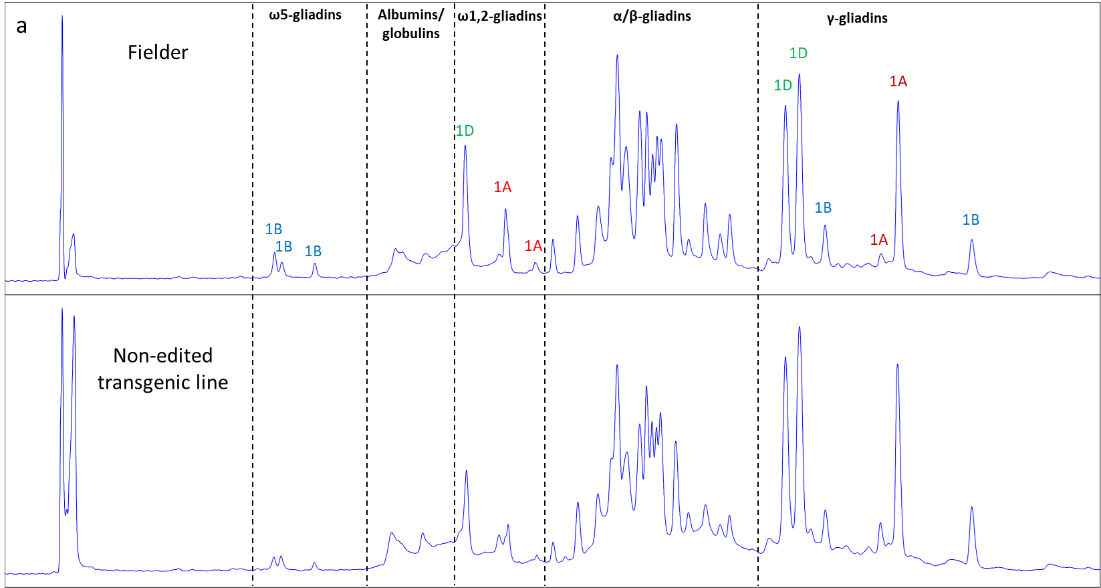


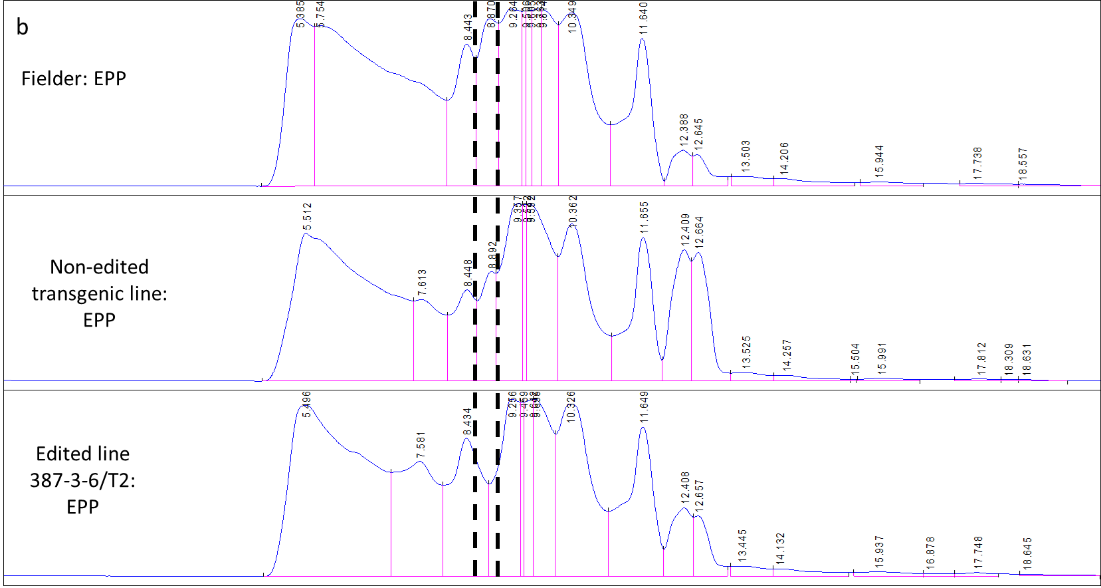


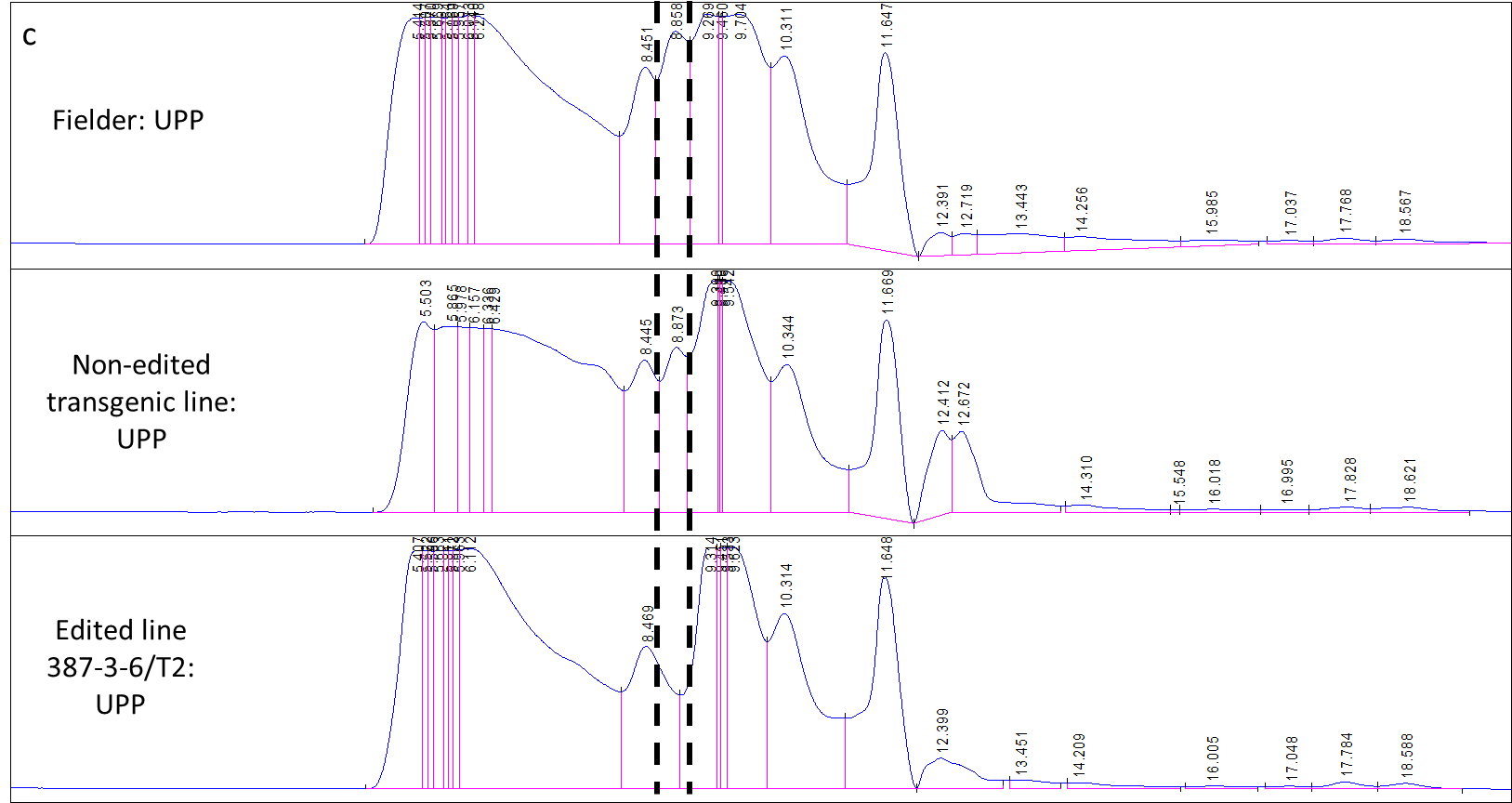


**Supplementary Figure 5.** Protein profiles of the non-edited transgenic segregant and cultivar Fielder. The comparison in protein profiles generated by RP-HPLC assay for three gliadin subtypes between a non-edited transgenic segregant and cultivar Fielder shows that they are completely identical in the number of peaks (**a**), indicating the tissue culture did not induce any changes in gliadin profile, which is also reflected by the results of comparisons in protein profiles generated by SE-HPLC assay for SDS-extractable (EPP; **b**) and un-extractable (UPP; **c**) polymeric proteins between non-edited transgenic segregants and cultivar Fielder, which are as the same as each other in both profiles of EPP and UPP. Therefore, the process of plant regeneration after plant transformation did not have any impact on gliadin profiles.


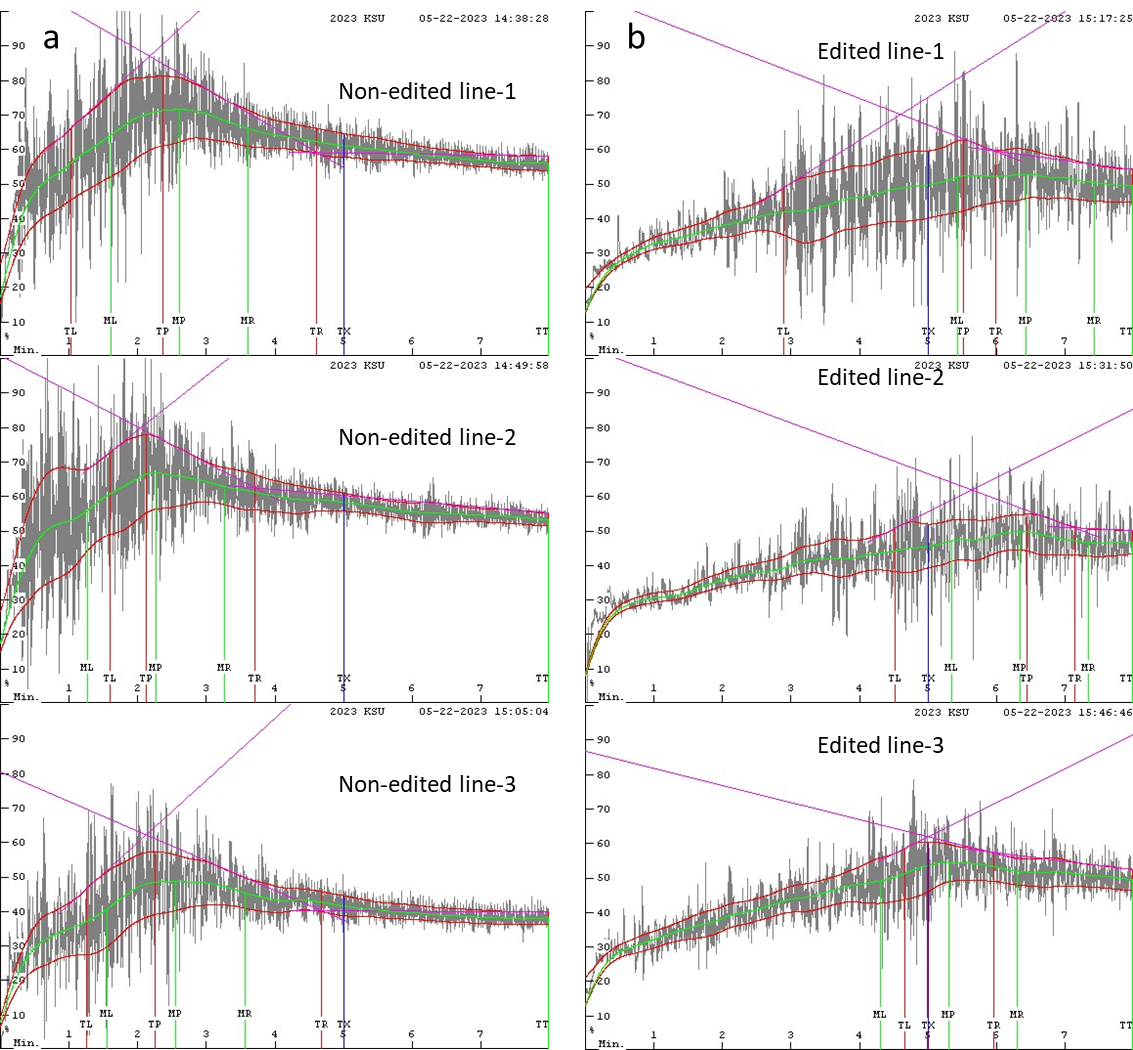


**Supplementary Figure 6.** Mixograph curves of dough developed from the flours of non-edited transgenic line and edited line 387-3-6. The MP is the short name of midline peak time, indicating the dough development time from the moment water is mixed with flour to the timepoint wheat dough reaches maximum consistency. The MPs for three biological replications of the edited line (**b**) are significantly higher than those for the three biological replications of non-edited transgenic line (**a**), indicating the amount and/or the size of gluten macropolymer in edited line are higher than those in the non-edited transgenic line, as shown in Supplementary Table 9.

**Supplementary Tables**

**Supplementary Table 1.** Number of R5 and G12 mAbs binding toxic epitopes detected within the gliadin-encoding genes from different wheat cultivars.

| **Sequence** | **Cultivar** | **Gliadin subtype** | **R5** | **G12** |
| --- | --- | --- | --- | --- |
| Complete sequence | Fielder | ω-gliadins | 253 | 128 |
|  |  | α/β-gliadins | 263 | 26 |
|  |  | γ-gliadins | 234 | 92 |
|  | Kariega | ω-gliadins | 163 | 72 |
|  |  | α/β-gliadins | 245 | 22 |
|  |  | γ-gliadins | 195 | 64 |
| Incomplete sequence | Chinese Spring | ω-gliadins | 54 | 23 |
|  |  | α/β-gliadins | 185 | 18 |
|  |  | γ-gliadins | 140 | 61 |
|  | LongReach Lancer | ω-gliadins | 85 | 33 |
|  |  | α/β-gliadins | 156 | 8 |
|  |  | γ-gliadins | 113 | 45 |

**Supplementary Table 2.** Potential gRNA target sites within the gliadin genes (external Excel file).

**Supplementary Table 3.** List of PCR primers used in the study.

| **Purpose** | **Primer** | **Sequence** | **Amplified fragment length** |
| --- | --- | --- | --- |
| Fragment deletions | 1A1DmiseqF-1 | CTCTTTCCCTACACGACGCTCTTCCGATCTCGCTTCCCAGACCCAACAATCGT | 791, 830, 854, 872-bp |
|  | 1A1DmiseqR-4 | CTGGAGTTCAGACGTGTGCTCTTCCGATCTGCTTACCACCGATGCTTGTAAGACTA |  |
|  | FD1B114miseqF-3 | CTCTTTCCCTACACGACGCTCTTCCGATCTCGCTTGGAATTGCATACTCCACAAGA | 1303-bp |
|  | FD1B1216miseqR-1 | CTGGAGTTCAGACGTGTGCTCTTCCGATCTGCTTAAGGCCACTGATACTTATAACGT |  |
|  | FD1B114miseqF-3 | CTCTTTCCCTACACGACGCTCTTCCGATCTCGCTTGGAATTGCATACTCCACAAGA | 1322-bp |
|  | FD1B1216miseqR-2 | CTGGAGTTCAGACGTGTGCTCTTCCGATCTGCTTATCATAGGCCACAGATACTTA |  |
| Cas9 fragment | SpCas9F2 | agattacgaaggctccgctc | 296-bp |
|  | SpCas9R2 | gctgccgttatcgaatgtcc |  |
| gRNA fragment | Pvintron1F1 | TACCAATGATGACCTTATCTCTC | 198-bp |
|  | sgScaffoldR1 | TTCAAGTTGATAACGGACTAGC |  |
| NGS | 1A1DmiseqF-1 | CTCTTTCCCTACACGACGCTCTTCCGATCTCTGTACCCAGACCCAACAATCGT | 301, 325-bp |
|  | 1A1DmiseqR-1 | CTGGAGTTCAGACGTGTGCTCTTCCGATCTGCTTAGGTTGTTGGAGTTCAGGAAATAA |  |
|  | 1BmiseqF-2 | CTCTTTCCCTACACGACGCTCTTCCGATCTCTGTACAAGGAATTGCATACTCCACAA | 306-bp |
|  | 1BmiseqR-2 | CTGGAGTTCAGACGTGTGCTCTTCCGATCTGCTTAAATTCCTGTTGCGGCAATT |  |
|  | PCR_Truseq_Amp_F | AATGATACGGCGACCACCGAGATCTACACTCTTTCCCTACACGAC |  |
|  | PCR_Truseq_Amp_R_21 | CAAGCAGAAGACGGCATACGAGATCGAAACGTGACTGGAGTTCAGACG |  |
|  | PCR_Truseq_Amp_R_23 | CAAGCAGAAGACGGCATACGAGATCCACTCGTGACTGGAGTTCAGACG |  |

**Supplementary Table 4.** Primers for PCR-based screening of fragment deletions.

| Primer | Gene | Chromosome | Amplified fragment length | Number of targeted region |
| --- | --- | --- | --- | --- |
| 1A1DmiseqF-1&1A1DmiseqR-4 | FD1A_omega1 | 1A | 791-bp | 2 |
|  | FD1A_omega3 | 1A | 872-bp | 2 |
|  | FD1D_omega1 | 1D | 830-bp | 7 |
|  | FD1D_omega2 | 1D | 854-bp | 6 |
|  | FD1D_omega3 | 1D | 854-bp | 6 |
|  | FD1D_omega4 | 1D | 854-bp | 6 |
|  | FD1D_omega5 | 1D | 854-bp | 6 |
| FD1B114miseqF-3&FD1B1216miseqR-1 | FD1B_omega3 | 1B | 1303-bp | 4 |
| FD1B114miseqF-3&FD1B1216miseqR-2 | FD1B_omega5 | 1B | 1322-bp | 4 |

**Supplementary Table 5.** NGS based detection of editing events by sequencing PCR amplicons.

| Primer | Gene | Chromosome | Number of targeted regions | Type of editing event | Size of event |
| --- | --- | --- | --- | --- | --- |
| 1A1DmiseqF-1&1A1DmiseqR-1 | FD1A_omega1 | 1A | 2 | Deletion | 42- and 51-bp |
|  | FD1A_omega3 | 1A | 1 | Deletion | N/A* |
|  | FD1D_omega1 | 1D | 3 | Deletion | 111-bp |
|  | FD1D_omega2 | 1D | 3 | Deletion | 42-, 48-, and 72-bp |
|  | FD1D_omega3 | 1D | 3 | Deletion | 42-, 48-, and 72-bp |
|  | FD1D_omega4 | 1D | 3 | Deletion | 42-, 48-, and 72-bp |
|  | FD1D_omega5 | 1D | 3 | Deletion | 42-, 48-, and 72-bp |
| 1BmiseqF-2&1BmiseqR-2 | FD1B_omega3 | 1B | 1 | Deletion | 18- and 19-bp |
|  | FD1B_omega5 | 1B | 1 | Deletion | 18-bp |

N/A*: The size of the deleted fragments in FD1A_omega3 were not detected within a total of 17 reads using the PCR amplicons based NGS, mostly due to the occurrence of the long fragment deletion resulting in the targeted amplified region being deleted (Fig. 2, Supplementary Table 6).

**Supplementary Table 6.** Summary of gene editing events detected by whole genome sequencing in the ω- and γ-gliadin gene clusters and the coordinates of gliadin genes in published Fielder genome (external Excel file).

**Supplementary Table 7.** The ratio of read coverage calculated by dividing the depth of read coverage in non-edited line 387-1-8 to the depth of read coverage of edited line 387-3-6. The depth of read coverage was calculate in the 50 bp windows within each gliadin gene models (External file).

**Supplementary Table 8.** Raw data for generating figures to show the impacts of gene editing on the content of each gliadin subtype, parameters of protein extracts correlated with grain protein quality for breadmaking and immunoreactivity in Figure 3, and the table to show the impacts of gene editing on dough quality in Supplementary Table 9 (external Excel file).

**Supplementary Table 9.** The comparison of the dough quality between the non-edited transgenic segregants and the edited line (387-3-6).

| Sample | Mixograph | | Micro-Farinograph | | | |
| --- | --- | --- | --- | --- | --- | --- |
|  | Peak time (minutes) | Score (mixing tolerance) | Water absorption (14%) | Dough development time/peak time (minutes) | Stability time (minutes) | Mixing tolerance index (BU) |
| Non-edited line-1* | 2.61 | 3 | 59.0% | 1.7 | 2.2 | 187 |
| Non-edited line-2* | 2.26 | 4 | 56.8% | 1.2 | 3.2 | 87 |
| Non-edited line-3* | 2.56 | 3 | 61.2% | 1 | 2 | 167 |
| 387-3-6/T3-1 | 6.43 | 4 | 52.2% | 4.5 | 11 | 34 |
| 387-3-6/T3-2 | 6.35 | 2 | 53.0% | 2.3 | 9.9 | 45 |
| 387-3-6/T3-3 | 5.3 | 2 | 51.9% | 2 | 7.3 | 45 |
| t-test p-value | 0.01 | 0.44 | 0.03 | 0.17 | 0.02 | 0.07 |
| Average (Non-edited lines) | 2.48 | 3.33 | 59.0% | 1.30 | 2.47 | 147 |
| Average (387-3-6/T3) | 6.03 | 2.67 | 52.0% | 2.93 | 9.40 | 41.3 |

*Flour was prepared from the seeds obtained from T_2_ Fielder lines that underwent regeneration after transformation and were determined not to carry the Cas9 gene constructs and editing events in gliadin genes.

**Supplementary Table 10.** The differences in the functional storage protein gene copy number between wheat 1BS and rye 1RS.

| **Species** | **cultivar** | **ω-gliadins/secalins** | **γ-gliadins/secalins** | **LMW-GS** |
| --- | --- | --- | --- | --- |
| Wheat 1BS | Fielder | 2 | 8 | 4 |
| Rye 1RS | Weining | 3 | 5 | 0 |
