# Supplementary figures and images for: CRISPR‐based editing of the ω‐ and γ‐gliadin gene clusters reduces wheat immunoreactivity without affecting grain protein quality
Source: Plant Biotechnol J. 2023 Nov 17;22(4):892–903. doi: 10.1111/pbi.14231 (PMC10955484; doi:10.1111/pbi.14231)

## Slide 1
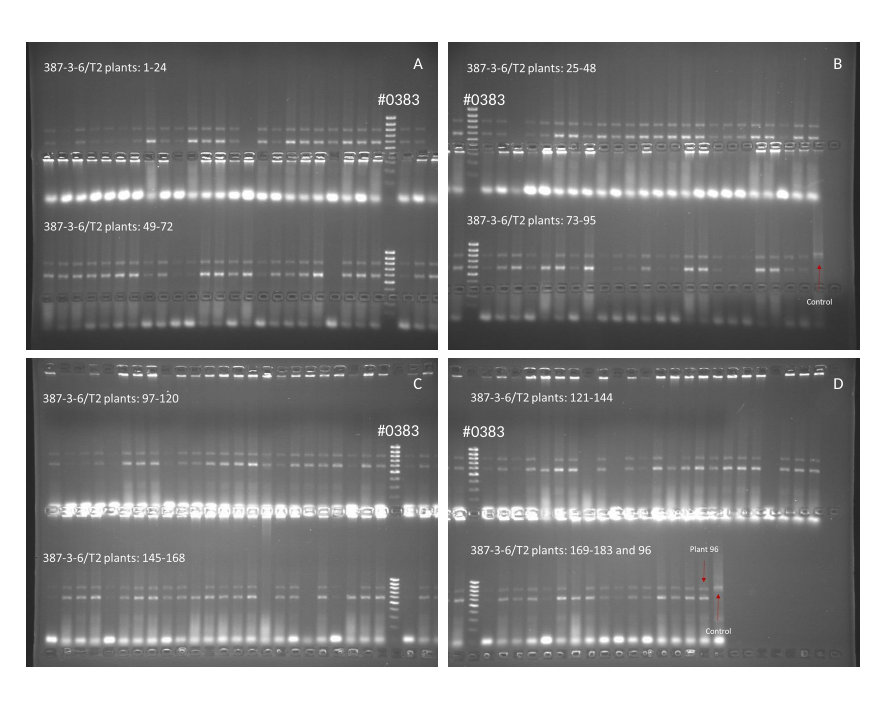

A
B
#0383
#0383
C
D
#0383
#0383

Supplement: Supplementary file 1 — Figure S1 The distributions of the 17 toxic epitopes including 11 binding to R5 mAb and six binding to G12 mAb across all three gliadin subtypes of cultivars Kariega, Chinese Spring and LongReach Lancer. Figure S2 PCR‐based screening of fragment deletions in five functional ω‐gliadin gene copies in T1 generation plants. Figure S3 PCR‐based screening of fragment deletions in five functional ω‐gliadin gene copies in T2 generation plants. Figure S4 NGS‐based detection for fragment deletions in the ω‐gliadin genes of edited line 387‐3‐6 using PCR amplicons. Figure S5 Protein profiles of the non‐edited transgenic line and cultivar Fielder. Figure S6 Mixograph curves of dough developed from the flours of non‐edited transgenic line and edited line 387‐3‐6. Table S1 Number of R5 and G12 mAbs binding toxic epitopes detected within the gliadin‐encoding genes from four wheat cultivars including Fielder, Kariega, Chinese Spring and LongReach Lancer. Table S2 Potential gRNA target sites within the gliadin genes (external Excel file). Table S6 Summary of gene editing events detected by whole genome sequencing in the ω‐ and γ‐gliadin gene clusters and the coordinates of gliadin genes in published Fielder genome (external Excel file). Table S7 The ratio of read coverage calculated by dividing the depth of read coverage in non‐edited line 387‐1‐8 to the depth of read coverage of edited line 387‐3‐6. The depth of read coverage was calculated in the 50 bp windows within each gliadin gene models (External file). Table S8 Raw data for generating figures to show the impacts of gene editing on the content of each gliadin subtype, parameters of protein extracts correlated with grain protein quality for breadmaking and immunoreactivity in Figure 3, and the table to show the impacts of gene editing on dough quality in Table S9 (external Excel file). Table S3 List of PCR primers used in the study. Table S4 Primers for PCR‐based screening of fragment deletions. Table S5 NGS‐based detection for [file PBI-22-892-s001.zip › Supplementary_Figure3_updated.pptx]
